# Fungal Frontiers in (Bio)sensing

**DOI:** 10.3390/bios16020131

**Published:** 2026-02-22

**Authors:** Gerardo Grasso

**Affiliations:** 1 Istituto per lo Studio dei Materiali Nanostrutturati, Sede Roma-Sapienza, Consiglio Nazionale delle Ricerche, P.le Aldo Moro 5, 00185 Rome, Italy; gerardo.grasso@cnr.it; 2 Nuova Micologia—Associazione di Studi Micologici—NPO, Viale dello Scalo S. Lorenzo, 16, 00185 Rome, Italy

**Keywords:** fungal secretome, mycosynthesis, mycelium-based materials, fungal electrophysiology, bio-inspired computing, living sensors, artificial intelligence, wood wide net

## Abstract

Filamentous fungi are increasingly recognized as versatile biological platforms for the development of advanced (bio)sensing technologies, owing to their extensive secretory capacity, material-forming ability, and intrinsic bioelectrical activity. This review critically surveys recent progress in fungal-based sensing within a multiscale framework spanning molecular, material, computational, and ecological domains, with particular emphasis on developments reported over the past five years. Key advances involving secretome-derived biomolecules, mycogenic nanomaterials, mycelium-based living materials, and fungal electrophysiology are discussed alongside emerging approaches for environmental monitoring that integrate sensor networks, imaging platforms, and data-driven analytics. Collectively, these works demonstrate that fungal systems can enhance biosensor sensitivity, selectivity, and sustainability, while enabling unconventional paradigms of signal transduction, material-integrated sensing, and biologically mediated computation. At larger spatial and temporal scales, mycelial growth dynamics and electrical activity provide measurable responses to mechanical, chemical, and environmental perturbations, supporting early applications in wearable devices, structural materials, and ecosystem monitoring. Despite significant progress, challenges remain in reproducibility, long-term stability, mechanistic understanding, and scalable device integration. Overall, the evidence reviewed highlights filamentous fungi as biologically adaptive and ecologically embedded systems with substantial potential to support next-generation (bio)sensing technologies, while underscoring the need for integrative approaches that combine biological insight with materials science, electronics, and artificial intelligence.

## 1. Introduction

Fungi are ubiquitous across nearly all habitats on Earth and have long played silent yet transformative roles in human civilization. Fungal fermentation represents one of the earliest and most influential biotechnological practices and continues to underpin diverse food, pharmaceutical, and industrial processes [1,2]. Beyond fermentation, filamentous fungi such as *Aspergillus*, *Penicillium*, and *Trichoderma* have become widely used as scalable and cost-effective biocatalytic systems across multiple industrial sectors [3]. Despite these profound contributions, the fungal kingdom remained, for much of scientific history, relatively understudied. Over the past decade, however, fungi have undergone an intellectual and cultural renaissance, driven by advances in biology, materials science, and biotechnology, reshaping both scientific inquiry and broader perspectives on their ecological and technological relevance [4,5,6,7,8,9,10,11,12,13,14,15,16]. In this context of renewed global interest, the harnessing of fungi in biosensing is re-emerging as a distinct frontier with deep historical roots. It is widely inferred that Clark and Lyons employed fungal glucose oxidase (GOx) in the development of the first biosensor. Although the original article did not specify the enzyme source, glucose oxidase at that time was produced exclusively through fungal fermentation, most notably from *Aspergillus niger* [17,18]. From early amperometric devices onward, fungal enzymes have continued to play a central role in biosensor development, later emerging as key biocatalysts in third-generation systems based on direct electron transfer (DET) mechanisms [19,20]. In addition to fungal enzyme-based biosensors, whole-cell fungal biosensors based on yeasts have evolved from early respiration-based systems to advanced synthetic biology platforms [21,22,23,24,25,26]. Despite significant progress, only a limited number have reached application readiness, primarily due to challenges related to robustness, immobilization, and standardization. Nanomaterials are widely used in biosensing because their high surface-to-volume ratio and size-dependent electronic and optical properties enable enhanced sensitivity and signal amplification [27,28,29]. In this context, growing evidence indicates that many filamentous fungi and yeast species are capable of converting metal ions or precursor compounds into well-defined nanostructures, including nanoparticles, nanowires, and quantum dots. This intersection, often referred to as *myconanotechnology*, enables low-energy, environmentally benign nanomaterial fabrication under mild and sustainable processing conditions. Fungal-derived nanomaterials frequently exhibit distinctive surface chemistries resulting from capping by fungal proteins, polysaccharides, or secondary metabolites, which is particularly advantageous for biosensing due to improved biocompatibility, stability, and functionalization. Consequently, myconanotechnology is attracting increasing attention, positioning fungi as versatile contributors to next-generation nanobiotechnology across diverse application domains, including biosensing [30,31,32]. This review consolidates recent advances by examining the scientific literature from the past five years on biosensing applications of filamentous fungi, beginning with fungal enzyme-based systems and extending to the fabrication of advanced nanomaterials. Filamentous fungi, including both mushrooms and molds, are composed of elongated filamentous structures called hyphae that grow and branch into complex networks known as mycelia. Compared with unicellular systems, filamentous fungi offer distinct advantages, including spatially distributed architectures, enhanced potential for bioelectronic integration, and emergent electrophysiological behaviors, making them particularly well suited for advanced (bio)sensing applications. Accordingly, this review highlights the emerging role of mycelium in (bio)sensing, positioning it as a versatile biomaterial whose structural, chemical, and physiological properties open new and largely unexplored avenues for technological innovation. Recent studies indicate that fungal mycelia can self-assemble into lightweight, biodegradable composites, providing sustainable alternatives to conventional foams, plastics, and fiberboards. Beyond serving as structural matrices, mycelial networks also exhibit dynamic responsiveness: experimental evidence shows that mycelial electrical activity can respond to environmental cues such as moisture, volatile compounds, and mechanical stress. These behaviors suggest their potential to function as living interfaces in sensing applications. Early work in fungal bioelectronics has further demonstrated that mycelial networks can propagate patterned electrical signals and modulate conductivity, supporting their promise as biologically derived platforms for biosensor signal transduction [33,34,35,36]. Collectively, these findings position mycelium and filamentous fungi as a compelling foundation for intelligent biomaterials and next-generation biohybrid sensing technologies with real-world relevance.

## 2. Methodology

**Figure 1:**
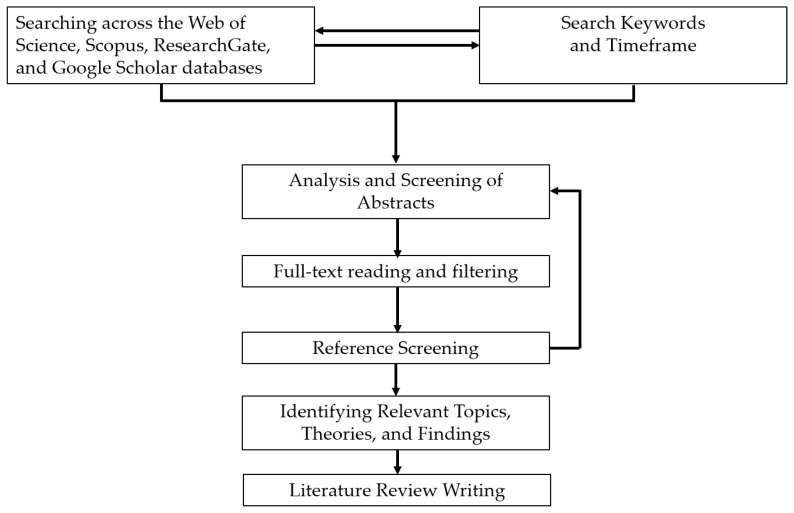
Methodological overview of the literature review process.

This review systematically examined the scientific literature published between 2019 and 2025. Major scientific databases, including Web of Science, Scopus, and Google Scholar, as well as literature-sharing platforms such as ResearchGate, were queried using defined combinations of search terms. Representative search strings included: (“fungal biosensing” OR “fungi biosensing”) AND (“laccase” OR “fungal enzymes” OR “fungal secretome”), (“fungal nanomaterials” OR “mycogenic nanomaterials” OR “fungal-derived nanoparticles”) AND “biosensing”, (“mycelium” AND “biosensing”), (“mycelium living material” OR “mycelium composite”), (“mycelium” AND “electrical spiking” OR “electrophysiology”), (“mycelium” AND “artificial intelligence” OR “unconventional computation”). To ensure comprehensive coverage, the bibliographies of key publications were manually screened to identify additional relevant references. Research on yeast-based biosensors, which is already extensively documented, was intentionally excluded to focus this review on filamentous fungi—an emerging and comparatively underexplored platform for (bio)sensing applications. All retrieved studies were critically assessed for relevance and methodological rigor. The selected literature was then organized into thematic categories, including enzymatic biosensing, nanomaterial-based sensing, and bioelectrical signal processing, to provide a structured synthesis aligned with the objectives of this review (Figure 1).

## 3. Fungal Secretome: A Valuable and Yet Underexplored Resource for Biosensing

Filamentous fungi are exceptionally versatile organisms with an extraordinary ability to colonize virtually every habitat. This extensive ecological success, along with their physiological and biochemical plasticity, is primarily supported by their capacity to secrete a broad range of biomolecules collectively known as the *fungal secretome*. The secretome comprises diverse components—including freely released proteins, cell wall-anchored proteins, signaling proteins, enzymes, and secondary metabolites—that collectively mediate nutrient acquisition, defense mechanisms, cell wall construction and remodeling, reproduction, and pathogenesis. Through these secreted molecules, fungi effectively interact with other organisms and modify the substrates within their environment. The remarkable metabolic flexibility of fungi is achieved through modulation of their secretome via diverse regulatory and secretory pathways. This adaptability enables them to respond efficiently to fluctuating environmental conditions, such as changes in available carbon and nitrogen sources, which are characteristic of the dynamic environments encountered by most soil fungi. The ubiquity of fungi across ecosystems, combined with their extensive evolutionary diversification, has resulted in a wide range of nutritional strategies—including saprophytic lifestyles (exploiting dead organic matter) and symbiotic or parasitic interactions with plants, insects, and animals. Each lifestyle requires a specialized suite of enzymes capable of degrading the complex biopolymers characteristic of the respective ecological niche. Through these capabilities, fungi serve as essential ecological regulators, driving organic matter decomposition and contributing substantially to nutrient cycling within ecosystems [37]. These same enzymatic capacities also enable fungi to biotransform a broad array of xenobiotic compounds, including dyes, agrochemicals, and per- and polyfluoroalkyl substances (PFASs) [38]. The field of mycology is being redefined by a wave of advanced technologies that move beyond traditional morphology-based methods of fungal identification. A key innovation is the adoption of molecular approaches—such as high-throughput sequencing and metagenomics—which have become essential for exploring the vast fungal diversity previously hidden from view. This includes the so-called “dark taxa,” groups of fungi that lack easily discernible physical characteristics and can now be resolved through their unique DNA signatures. A shift toward a more holistic understanding of fungal biology is further exemplified by the rise of multiomics. This integrated framework combines data from genomics, transcriptomics, proteomics, and related disciplines to provide a comprehensive systems-level view of fungal function. These approaches have opened new perspectives across diverse applications, from characterizing fungal communities in natural and engineered environments to developing innovative biotechnological solutions. Advanced omics tools are thus not only transforming fungal taxonomy and ecology but are also unlocking the potential of fungi to address critical global challenges in health, food security, and environmental management [39]. Although omics-based platforms—including metagenomics, genomics, transcriptomics, and proteomics—have significantly advanced the discovery and characterization of novel fungal enzymes, their full exploitation remains limited. These methodologies could be more systematically applied to expand databases of fungal-derived biomolecules and to stimulate further research in this field [40]. In particular, the evolution of unique enzymes in fungi adapted to extreme environments (e.g., deserts, acidic soils, or metal-rich habitats), along with the discovery of previously unknown enzymatic activities, represents a vast and largely untapped resource with substantial potential for both fundamental mycological research and future biotechnological innovation [41]. A deeper understanding of fungal responses to environmental fluctuations, their molecular expression profiles, and the mechanisms underlying metabolite production is therefore essential for advancing both basic mycology and its technological applications. As highlighted in this review, the diverse enzymatic repertoire and broad substrate-interaction capacity of the fungal secretome position fungi as promising contributors to emerging biosensing strategies.

### 3.1. Enzymes

Fungal enzymes are among the most prominent and functionally diverse components of the fungal secretome. Most fungal enzymes have been extensively characterized for their roles in industrial processes, such as those in the food and pharmaceutical sectors, as well as in white biotechnology more broadly [42]. These enzymes (Table 1) can catalyze a wide range of biochemical reactions, and their unique catalytic properties make them particularly well suited for advanced biosensing technologies. Their high specificity and sensitivity are essential for the accurate detection of target molecules in complex matrices. Incorporating fungal enzymes into biosensing platforms enables the detection of diverse analytes, including environmental pollutants, biomarkers, and food contaminants, thereby enhancing the performance and applicability of modern biosensing systems [43,44,45].

#### 3.1.1. Oxidoreductases

**Figure 2:**
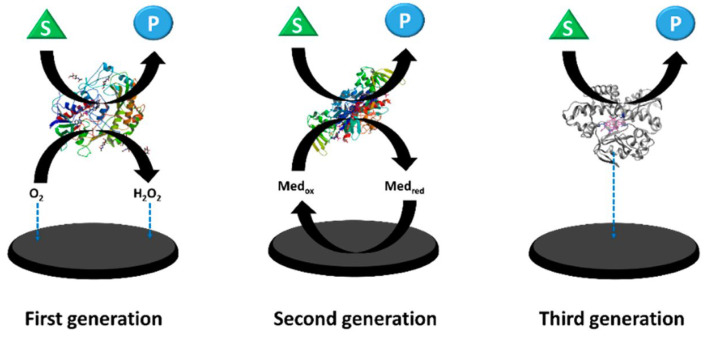
A schematic representation of first-, second-, and third-generation amperometric enzymatic biosensors. The first generation relies on the electroactivity of either the receptor substrate (S) or its product (P). The second generation utilizes artificial redox mediators (Med_red_/_ox_), while the third generation involves direct electron transfer between the redox-active biomolecule and the electrode. Reproduced from Ref. [44].

Over twenty distinct classes of fungal oxidoreductases have been identified to date. Among these, the most extensively studied and documented are dehydrogenases (which transfer hydrogen to an electron acceptor), oxygenases (in which oxygen serves as the final electron acceptor), and peroxidases (in which peroxides act as the final electron acceptors). Enzymes with commercial applications include monooxygenases, dioxygenases, and laccases. The most widely used enzyme in the development of first-generation electrochemical glucose biosensors is glucose oxidase (GOx). Initially isolated from *Aspergillus niger*, GOx is now also produced industrially by the filamentous fungus *Penicillium amagasakiense*. Additional fungal sources of GOx reported in the literature include *Aspergillus oryzae*, *Penicillium notatum*, *Penicillium glaucum*, *Phanerochaete chrysosporium*, and *Talaromyces flavus* [46,47]. Some fungal glucose oxidases exhibit noteworthy characteristics. For example, the safe applicability of GOx derived from *Penicillium chrysogenum* in commercial food processing has been reported by Konishi et al. [48]. More efficient glucose-catalyzing kinetics have been described for recombinant glucose oxidase from *Penicillium amagasakiense* expressed in *Pichia pastoris*, which exhibited a kcat/KM value of 93 µM^−1^ s^−1^, compared with a kcat/KM of 27 µM^−1^ s^−1^ for the native enzyme from *Aspergillus niger* [49]. Glucose oxidase (GOx) consumes oxygen during catalysis, and fluctuations in dissolved oxygen concentration can interfere with measurements, thereby reducing biosensor accuracy. To address this limitation, second-generation electrochemical sensors (Figure 2) primarily employed redox mediators to minimize oxygen interference; however, oxygen could still pose challenges.

An alternative approach involved the use of oxygen-insensitive glucose dehydrogenases (GDHs), although these introduced new challenges related to cofactor diffusion and substrate specificity. Flavin adenine dinucleotide-dependent glucose dehydrogenases (FADGDHs), which utilize FAD as a redox cofactor, represent promising candidates for third-generation electrochemical sensors (Figure 2) used in self-monitoring blood glucose applications. Nevertheless, achieving direct electron transfer with FADGDHs remains a significant obstacle. Direct electron transfer (DET) is essential for the development of advanced third-generation continuous glucose monitoring systems, as it eliminates the need for mediators and minimizes oxygen-related interference [50]. Ito et al. developed a genetic engineering strategy to enable DET by fusing *Aspergillus flavus* FADGDH with the heme b-binding cytochrome domain of *Phanerochaete chrysosporium* cellobiose dehydrogenase and expressing the resulting construct in *Pichia pastoris*. Spectroscopic analysis of the purified enzyme confirmed intramolecular FAD–heme electron transfer, which was enhanced at lower pH and in the presence of divalent cations. When immobilized on an electrode, the engineered FADGDH generated a high current density (≈400 µA cm^−2^ at 50 mM glucose) and exhibited a glucose concentration-dependent response up to 50 mM, thereby demonstrating direct electron transfer absent in the wild-type AfGDH. Importantly, the engineered enzyme retained the substrate specificity of AfGDH and showed no oxidase activity. The biosensor also displayed low interference from common electroactive species such as ascorbic acid and uric acid. These findings highlight a promising strategy for enhancing the direct electron transfer capabilities of FADGDH in future biosensing technologies [51]. *Talaromyces emersonii* (recently reclassified as *Rasamsonia emersonii*) is a thermophilic, aerobic fungus known for producing a range of hydrolytic enzymes, particularly cellulases and xylanases, which are widely applied in biofuel production, food processing, and the textile industry. Cohen et al. [52] developed an amperometric biosensor based on *T. emersonii* FAD-glucose dehydrogenase and evaluated its performance in both mediated and direct electron transfer modes. The study highlighted the enzyme’s capacity for oxygen-independent glucose detection, effectively overcoming the oxygen-related limitations of GOx-based sensors and challenges associated with other GDHs. Using a polydopamine encapsulation matrix and redox mediators, the biosensor achieved reliable operation with a linear response up to 20 mM glucose, minimal interference, and stable performance for over 20 h. These findings underscore the potential of *T. emersonii* FADGDH as a robust biocatalyst for next-generation biosensing and biofuel cell applications. Wijayanti et al. developed an innovative maltose biosensor based on an oxygen-independent FAD-glucose dehydrogenase from *Trichoderma virens*, a well-studied fungal biocontrol agent commercially used as a biopesticide, biofertilizer, and soil amendment. This enzyme displayed a unique substrate preference for maltose over glucose, providing a robust single-enzyme alternative to more complex multi-enzyme systems. For sensor fabrication, the FADGDH was incorporated into a third-generation electrode by entrapment and wiring with an osmium redox polymer on a graphite electrode to enable mediated electron transfer. The enzyme demonstrated negligible oxygen activity and a low redox potential (−0.268 ± 0.007 V vs. SHE), allowing efficient pairing with higher-potential redox mediators for effective electron transfer. The biosensor exhibited a sensitivity of 1.7 µA mM^−1^ cm^−2^ for maltose, a linear detection range of 0.5–15 mM, a detection limit of 0.45 mM, and an apparent Michaelis–Menten constant (Km) of 19.2 ± 1.5 mM for maltose. Although optimized for maltose, the sensor also responded to other saccharides, including glucose, maltotriose, and galactose, which may introduce interference [53]. Redox potential is a critical parameter for the effective application of oxidoreductases in biosensing, as it influences both the minimization of electroactive interference and the maximization of current density or cell voltage. Although FAD-dependent glucose dehydrogenases (FADGDHs) from the glucose-methanol-choline (GMC) oxidoreductase family are widely used in glucose biosensors, their redox potentials are often unreported. This is primarily because FAD centers are typically buried within the protein structure, making them inaccessible to direct electrochemical techniques such as cyclic voltammetry, and because identifying suitable diffusible mediators is challenging. Schachinger et al. addressed this gap by determining the redox potential of GDH from *Glomerella cingulata* (a fungal plant pathogen) using spectroelectrochemical methods in combination with a xanthine oxidase assay. The reported low redox potential (−0.265 ± 0.003 V vs. SHE), together with the enzyme’s high substrate specificity and oxygen insensitivity, provides essential guidance for its rational incorporation into biosensors employing redox mediators or polymers, optimizing both sensor performance and signal reliability [54].

Pyranose oxidase (POx, also referred to as pyranose 2-oxidase, P2Ox), an FAD-dependent oxidoreductase, was categorized within the glucose-methanol-choline (GMC) superfamily, similarly to GOx. Fungal POx offers complementary advantages as an FAD-dependent GMC oxidoreductase. Typically, extracellular and often membrane-associated, fungal POx participates in lignin degradation and antimicrobial hydrogen peroxide generation. Its primary activity is the oxidation of aldopyranoses, including D-glucose, D-galactose, and D-xylose, at the C2 position, producing 2-keto sugars and hydrogen peroxide. POx also utilizes alternative electron acceptors such as quinones and metal ions, broadening its biotechnological relevance. Kinetic analyses indicate high substrate affinity for D-glucose (Km 0.74–5.0 mM) and turnover numbers (kcat 1.48–111 s^−1^), surpassing GOx in several catalytic aspects. However, fungal POxs are tetrameric, potentially limiting active site accessibility, whereas bacterial POxs are monomeric or dimeric, favoring more efficient interactions with electrodes or mediators. Because of these limitations, POx has been less frequently applied in biosensors compared to FADGDHs [55]. A study from Abrera et al. [56] characterized engineered variants of POx from *Trametes ochracea* designed to enhance electron acceptor turnover and reduce oxygen reactivity, addressing the challenge of oxygen competition in bioelectrocatalytic applications. The study evaluated pre-steady-state kinetics of variants T166R, Q448H, L545C, and L547R with electron acceptors including 1,4-benzoquinone, 2,6-dichlorophenol indophenol, and ferrocenium ions. The engineered POx variants demonstrated increased electron acceptor turnover alongside diminished oxygen activity, highlighting their potential as efficient anode biocatalysts for biosensors and biofuel cells. These modifications enhance the enzyme’s suitability for electrochemical applications by minimizing oxygen interference, a common limitation in conventional oxidase-based systems. In a recent study, Punthong et al. provided the first comprehensive characterization of 2-keto-aldonic acid production by P2Ox from *Trametes multicolor*, elucidating the molecular mechanisms underlying its catalysis. P2Ox specifically oxidizes the C2 position of pyranose sugars, converting them to 2-keto sugars while reducing oxygen to hydrogen peroxide. The enzyme was shown to act on a variety of sugars—including D-glucose, D-xylose, D-galactose, and L-arabinose—producing 2,3-diketo-glucose, 2,3-diketo-xylose, 2-keto-galactonic acid, and 2-keto-arabinonic acid, respectively. This work represents the first detailed investigation of P2Ox’s secondary oxidation of 2-keto sugars and highlights its potential as an efficient chemo-enzymatic strategy for the synthesis of sugar acids [57]. These findings have significant implications for biosensing, providing a foundation for the development of robust, selective, and efficient sensors capable of detecting 2-keto sugars or their downstream oxidation products.

**Figure 3:**
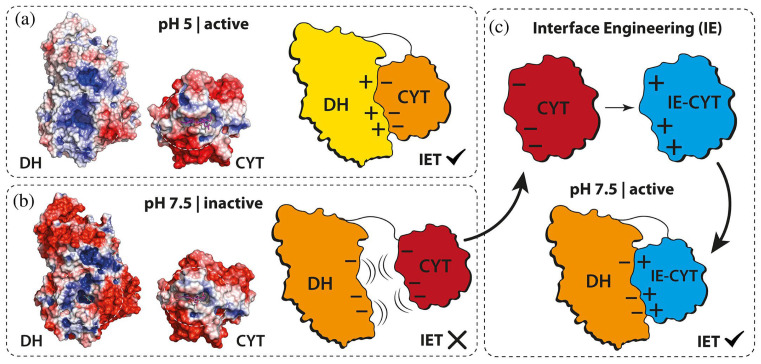
Surface electrostatics and interface engineering in cellobiose dehydrogenase (CDH). (**a**) Surface electrostatic profiles and schematic representation of pH-dependent interdomain interactions between the dehydrogenase (DH) and cytochrome (CYT) domains at pH 5 (active state), highlighting favorable electrostatic complementarity and efficient interdomain electron transfer (IET). Electrostatic surface potentials are shown as a red–blue color gradient, where red indicates negatively charged regions and blue indicates positively charged regions. The check mark (✓) denotes IET-active states. (**b**) Surface electrostatic profiles and schematic representation of DH–CYT interactions at pH 7.5 (inactive state), showing electrostatic repulsion and impaired IET due to altered surface charge distribution. Red and blue areas denote negatively and positively charged surface regions, respectively. The cross (X) indicates loss of IET activity. (**c**) Interface engineering (IE) strategy to modulate the CYT surface charge distribution at pH 7.5, generating a positively charged IE-CYT domain that restores productive DH–CYT interactions and enables IET.Reproduced from Ref. [61] (Reichhart et al.) under the Creative Commons Attribution License (CC BY 4.0).

Another auxiliary enzyme supporting lignin oxidation is cellobiose dehydrogenase (CDH), which also serves as a valuable fungal bioelectrocatalyst in biosensors. Its utility stems from a distinctive structural feature—a mobile cytochrome domain—that enables direct electron transfer to electrode surfaces, thereby eliminating the need for external electron mediators. [58]. A recent study introduced an oxygen-insensitive amperometric glucose biosensor using an engineered CDH from *Crassicarpon hotsonii* (a thermophilic ascomycete fungus), modified to enhance glucose specificity. The enzyme was integrated into sensors operating via direct and mediated electron transfer. The mediated electron transfer biosensor, incorporating an osmium-based redox polymer, outperformed direct electron transfer in terms of sensitivity (17.3 vs. 1.22 µA cm^−2^ mM^−1^), maximum current density (719 vs. 21.8 µA cm^−2^), and linear range (0–10 vs. 0–5 mM). However, the direct electron transfer system exhibited a lower apparent Michaelis-Menten constant (12.4 vs. 37.9 mM), indicating stronger substrate affinity. Both sensors showed low detection limits (~1.7–1.9 mM), stable operation over 12 h, and no response variation under ambient versus deoxygenated conditions, confirming oxygen insensitivity. Performance in artificial serum revealed reduced sensitivity (−43% for direct electron transfer, −28% for mediated electron transfer) due to protein adsorption and electrochemical interference, though stability was unaffected. Interference studies confirmed high specificity; no individual compound exceeded the 20% MARD threshold, though cumulative effects in complex media influenced signal response. These findings underscore the promise of engineered CDH for robust, oxygen-independent glucose sensing and highlight mediated electron transfer biosensors as superior in analytical performance under both standard and physiologically relevant conditions [59]. A critical challenge in developing implantable glucose biosensors for continuous monitoring in patients with diabetes mellitus is ensuring biosensor sterilization without compromising performance. Bennett et al. evaluated the effects of terminal sterilization—gamma irradiation (25 kGy, 260 Gy h^−1^) and ethylene oxide (EtO)—on glucose biosensors functionalized with either CDH from *Crassicarpon hotsonii* (syn. *Myriococcum thermophilum*) or GOx from *Aspergillus niger*. Electrodes were fabricated as carbon microarrays modified with osmium-complex redox polymers, with some incorporating a zwitterionic poly(2-methacryloyloxyethyl phosphorylcholine-co-glycidyl methacrylate) (MPC) coating to enhance biocompatibility. Cyclic voltammetry in 100 mM glucose revealed that gamma irradiation preserved sensor activity, with CDH-modified electrodes retaining 71% of their initial current, comparable to unsterilized controls. In contrast, EtO treatment caused a 70% signal loss, which was partially mitigated (~50% retention) by the MPC coating. GOx-based electrodes maintained function after gamma exposure. Mechanistic studies showed that gamma irradiation induced structural changes and cofactor loss in CDH, explaining its sensitivity, whereas EtO caused ~40% activity loss without altering protein conformation, likely through chemical modification of amino acids. GOx demonstrated greater structural stability under both treatments but lost ~40% activity after gamma exposure due to aggregation. No cytotoxic leachates were observed post-sterilization, except for minor effects from EtO on day one. Based on these findings, low-dose gamma irradiation emerged as the preferred sterilization method for maintaining biosensor integrity and function [60]. Despite its promising features, CDH faces limitations in practical physiological glucose measurements, such as in blood or other biofluids (e.g., sweat, tears), due to its acidic pH optimum and relatively slow interdomain electron transfer (IET) rates at physiological pH (~7.5) compared with its catalytic potential. To address these limitations, Reichhart et al. engineered CDH to enhance IET at physiological pH by rationally mutating acidic residues on the cytochrome (CYT) domain to reduce electrostatic repulsion (Figure 3).

**Figure 4:**
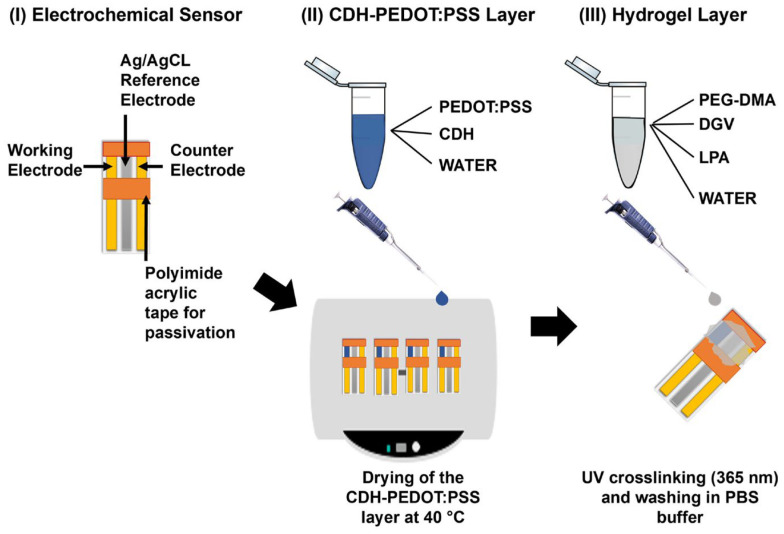
Schematic representation of the electrochemical sensor modification steps for glucose detection: (I) unmodified sensor, (II) application of the CDH–PEDOT:PSS layer, and (III) deposition of the hydrogel coating. Reproduced from Ref. [62].

Single and combinatorial mutations significantly increased IET rates; the most active multi-site variants achieved 1.24 s^−1^ at pH 7.5, compared with 0.1 s^−1^ for the wild-type enzyme. However, the accumulation of positive charges reduced direct electron transfer efficiency. IET rates were determined spectrophotometrically via cytochrome c reduction at 30 °C in McIlvaine buffer (pH 3.0–8.5) using 30 mM lactose and 50 µg mL^−1^ enzyme, while electrochemical characterization employed cyclic voltammetry and amperometry at 0.25 mV versus Ag/AgCl (0.1 M KCl) with 5 mM lactose for DET and 5 mM lactose plus 20 µM 1,4-benzoquinone for MET. Statistical analysis used one-way ANOVA (*p* < 0.05), and the limit of quantification for IET rates was 0.01 s^−1^. Notably, all experiments were conducted in vitro, without testing in real biological samples [61]. Despite its promising features, CDH faces limitations for physiological glucose measurements, such as in blood or other biofluids (e.g., sweat, tears), due to its acidic pH optimum and relatively slow interdomain electron transfer (IET) rates at physiological pH (~7.5) relative to its overall catalytic potential. Cihan et al. developed a third-generation glucose biosensor by immobilizing CDH within a conductive PEDOT:PSS layer covered by a PEG-DMA hydrogel on planar gold electrodes, enabling DET at low potentials. Their work did not involve protein engineering; instead, it focused on optimizing the immobilization strategy to improve enzyme stability, prevent leaching, and enhance electrochemical performance under physiological conditions (Figure 4). DET activity was evaluated electrochemically using cyclic voltammetry and chronoamperometry at potentials between −0.2 and 0.4 V versus Ag/AgCl in phosphate-buffered saline. The CDH-PEDOT:PSS–PEG-DMA layer produced a glucose-specific current response, exhibited minimal interference from common electroactive species at 0 V, and achieved a dynamic range of 0.1–20 mM with a limit of detection of 0.1 mM. All experiments were performed in vitro on modified gold or graphite electrodes, without evaluation in biological samples [62].

CDHs are naturally adapted to act on β-1,4-linked di- and oligosaccharides, such as lactose, which is a sugar of interest in both the dairy industry and food quality monitoring. Choi et al. developed an electrochemical lactose biosensor by immobilizing CDH within a chitosan composite incorporating Co-hemin metal–organic frameworks (MOFs), representing a novel immobilization strategy for this enzyme. The sensor exhibited high sensitivity (102.3 mM^−1^ cm^−2^) and a rapid response time of 5 s, with a limit of detection of 4 mM and a broad linear range from 10 to 100 mM, suggesting potential suitability for direct analysis of commercial dairy products. Electrochemical measurements were performed on glassy carbon electrodes using *Phanerochaete chrysosporium* CDH at 38 µg mL^−1^ in 100 mM sodium phosphate buffer (pH 7.0), yielding a linear calibration (y = 3.241x − 12.886; r = 0.998). While the results demonstrate the sensor’s potential, experiments were conducted in controlled buffer solutions rather than real biological samples, leaving practical validation in real dairy matrices untested [63]. Justyna et al. explored the use of natural microbial polysaccharides, both bacterial and fungal, including those from *Cerrena unicolor* and *Ganoderma applanatum*, to enhance the stability and catalytic properties of CDH from *Pycnoporus sanguineus*, a tropical and subtropical white rot fungus. Polysaccharide treatments significantly improved enzyme stability compared with controls, with Rh110EPS providing the greatest stabilization effect. The *C. unicolor* polysaccharide not only enhanced stability but also reduced CDH’s Km during storage over 15 and 30 days. Additionally, the antioxidative properties of CDH were evaluated in the presence of fungal polysaccharides for the first time in this context, with only the *C. unicolor* polysaccharide exhibiting strong free radical-scavenging activity, thereby boosting the enzyme’s antioxidant potential. Incubation with specific polysaccharide modifiers also altered CDH’s optimum pH. Electrochemical measurements using cyclic voltammetry revealed well-defined anodic and cathodic peaks for all polysaccharide variants, indicating improved stability under electrochemical conditions, with the highest peaks observed after 30 days at 4 °C; the *C. unicolor* polysaccharide produced the largest peak shifts. Enzyme activity was measured by lactose oxidation at 30 °C in 100 mM sodium acetate buffer (pH 4.5) using 2,6-dichloroindophenol (DCIP) or cytochrome c as electron acceptors. Kinetic parameters (Km, Vmax, kcat) were derived from Michaelis-Menten analysis using cellobiose (0.05–1 mM) and lactose (0.5–100 mM). Stability was assessed over 15, 30, and 60 days at 4 °C and 25 °C, and optimal pH was evaluated across 3.0–6.5. Antioxidant activity was measured via the DPPH assay. All experiments were conducted in vitro with purified components; real biological samples were not tested [64].

Pyranose dehydrogenase (PDH) is a flavin-dependent carbohydrate oxidoreductase that occurs relatively rarely, primarily in lignocellulolytic Basidiomycetes and Ascomycetes (including former Fungi Imperfecti). Unlike many oxidoreductases, PDH does not utilize oxygen as an electron acceptor, instead relying on substituted benzoquinones or (organo)metal ions. The enzyme exhibits broad substrate specificity and regioselectivity, catalyzing monooxidations at the C1, C2, or C3 positions and dioxidations at the C2,3 or C3,4 positions of various sugars. This combination of catalytic versatility and electron acceptor preference makes PDH a promising candidate for enzymatic sensors capable of detecting a wide range of sugars, as highlighted in a review by Peterbauer et al. [65]. However, the recent literature indicates that no studies on PDH-based biosensing applications have been published in the past five years. This apparent gap likely reflects practical limitations associated with PDH, including its restricted phylogenetic distribution, reliance on non-oxygen electron acceptors, limited availability and standardization compared to more established oxidoreductases, and challenges in achieving selective and operationally robust biosensor architectures. Together, these factors may have hindered the broader adoption of PDH in biosensing applications despite its attractive catalytic versatility, as discussed in detail by Peterbauer et al. [65].

Galactose oxidase (GOase) catalyzes the oxidation of D-galactose to 1,6-D-galactodialdose at a single copper redox site. Figueiredo et al. developed an oxygen-insensitive amperometric galactose biosensor using GOase derived from *Dactylium dendroides*, a fungus known as a primary causal agent of cobweb disease in *Agaricus bisporus*. The sensor employed a third-generation “wiring” strategy, co-immobilizing GOase with an Os-complex-modified redox polymer on screen-printed carbon electrodes to circumvent oxygen interference. Designed for accurate galactose determination in complex dairy products, the biosensor addressed a key need for reliable, cost-effective monitoring relevant to conditions such as galactosemia. Measurements performed in authentic dairy matrices at an applied potential of −0.15 V versus Ag/AgCl demonstrated a linear response from 0 to 100 µM galactose, with a sensitivity of 0.60 ± 0.05 A M^−1^ cm^−2^, a limit of detection of 1 µM, and a limit of quantification of 3 µM. Interference from common electroactive compounds in milk—including ascorbic acid, uric acid, and acetaminophen—was minimal, and no interference was observed from other sugars such as glucose or lactose. The sensor exhibited strong operational stability, retaining 90% of initial activity after 24 h, and storage stability, maintaining 85% activity after 7 days at 4 °C [66]. A highly sensitive and stable electrochemical nitrate biosensor was developed using nitrate reductase (NR) from the fungus *Neurospora crassa*. The NR was immobilized within a chitosan polymer matrix on a glassy carbon electrode, with anthraquinone sulfonate serving as an artificial electron mediator to facilitate efficient electron transfer. This configuration enabled high sensor performance, achieving a sensitivity of 25.4 A M^−1^ cm^−2^ via constant-potential amperometry. The biosensor exhibited a linear detection range up to 450 µM nitrate and a low detection limit of 1.2 µM. Remarkably, it retained over 70% of its activity after three months, demonstrating exceptional stability. Practical applicability was validated through reproducible measurements of nitrate in rainwater and river water samples [67].

Lignocellulose constitutes the primary structural component of plant biomass, comprising mainly cellulose (35–55% *w*/*w*), hemicellulose (20–40% *w*/*w*), and lignin (10–25% *w*/*w*). Its decomposition is crucial for carbon cycling in the biosphere. Fungi, particularly filamentous and wood-decaying species such as brown-rot and white-rot fungi, are among the most efficient lignocellulose degraders due to their robust extracellular enzymatic systems. Brown-rot fungi, which dominate wood decay in coniferous forests, primarily degrade cellulose and hemicellulose, leaving a modified lignin-rich residue. These organisms employ distinctive oxidative strategies, including non-enzymatic Fenton chemistry, to break down plant cell walls. In contrast to brown-rot and litter-decomposing fungi, white-rot fungi uniquely possess the ability to completely mineralize all lignocellulose components, including lignin [68]. White-rot fungi are major contributors to the global carbon cycle and include several species of economic importance. More than 50 mushroom species are cultivated commercially, and three of the four most widely produced edible mushrooms—*Lentinula edodes* (shiitake, 22%), *Pleurotus* spp. (oyster mushrooms, 19%), and *Auricularia* spp. (wood ear mushrooms, 18%)—are white-rot fungi valued for both their nutritional and medicinal properties [69]. White-rot fungi are the only known organisms capable of completely mineralizing lignin. To efficiently degrade such complex polymers, they have evolved sophisticated enzymatic systems collectively known as the lignin-degrading enzyme consortium. These ligninolytic enzymes are broadly classified into two groups: lignin-modifying enzymes (LMEs) and lignin-degrading auxiliary enzymes (LDAs). LMEs directly catalyze lignin breakdown and include laccases—phenol oxidases that specifically oxidize phenolic lignin subunits—as well as heme-containing peroxidases such as lignin peroxidase (LiP), manganese peroxidase (MnP), versatile peroxidase (VP), and dye-decolorizing peroxidase (DyP). LiP and VP can act on both phenolic and non-phenolic lignin structures, whereas MnP primarily targets phenolic components; all of these peroxidases utilize hydrogen peroxide (H_2_O_2_) to drive lignin oxidation. LDAs, in contrast, do not directly degrade lignin but support LMEs by generating reactive species, such as H_2_O_2_, that are essential for peroxidase activity. This group includes aryl-alcohol oxidase, glyoxal oxidase, pyranose 2-oxidase, glucose oxidase, and cellobiose dehydrogenase. Lignin degradation involves a complex enzymatic cascade, producing reactive intermediates such as aromatic radicals and oxidized metal ions, which can act as diffusible electron carriers. The precise mechanisms governing these synergistic interactions within the ligninolytic system remain an active area of research [70].

**Figure 5:**
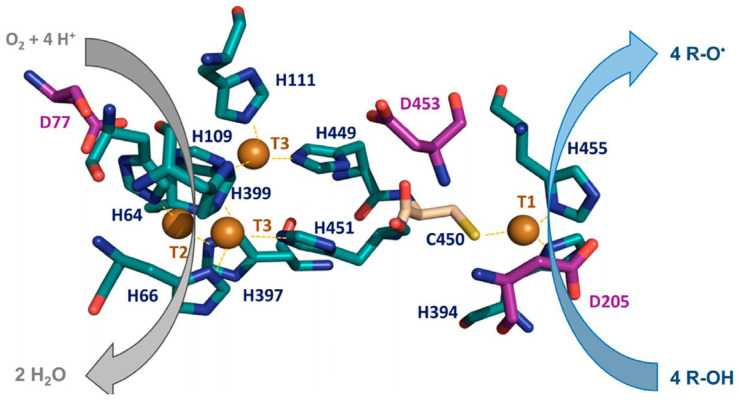
Molecular View of Electron Transfer from the Reducing Substrate at the T1 Site to the Tetranuclear Center (TNC) in PM1 laccase. The figure illustrates electron transfer from the reducing substrate to the tetranuclear center (TNC) of laccases. The coordination sphere of the four catalytic copper ions (spheres) is shown, highlighting the coordinating histidine (blue) and cysteine (wheat) residues, as well as the conserved acidic residues (purple) involved in electron-proton transfer. Reproduced from Ref. [71].

Laccases are among the most prominent members of the multicopper oxidase (MCO) family, characterized by their cupredoxin-like structural fold and ability to activate molecular oxygen. As the largest group within MCO family, laccases share a common catalytic mechanism involving oxygen activation. Fungal laccases contain four copper atoms arranged into a T1 mononuclear site for substrate oxidation and a T2/T3 trinuclear cluster for the reduction of molecular oxygen to water (Figure 5).

These enzymes exhibit high redox potentials (up to ~800 mV vs. SHE) and exceptional oxidative performance across diverse substrates, making them versatile biocatalysts. Fungal laccases, particularly those from white-rot basidiomycetes, have attracted significant attention due to their strong ability to transform a broad range of aromatic compounds, including lignin, and are typically extracellular enzymes. Their oxidation efficiency is often enhanced through redox mediators—small molecules that shuttle electrons between the enzyme and bulky or poorly accessible substrates. Through protein engineering, particularly via directed evolution, researchers have improved the adaptability of fungal laccases for targeted applications such as biosensing. Strategies such as rational design and random mutagenesis have enhanced catalytic efficiency, stability (across pH, temperature, and organic solvents), and substrate specificity. Engineered variants often display higher substrate affinity, increased activity, and improved resistance to extreme conditions [71]. Product recovery and purification remain major challenges in laccase production, as downstream processing can account for up to ~80% of total industrial enzyme manufacturing costs. Given this economic constraint and the expanding biotechnological applications of laccases, developing effective post-production purification strategies is essential. One approach to mitigate these costs involves using sustainable and low-cost agro-industrial by-products rich in lignin, cellulose, and hemicellulose as fungal substrates. These materials serve as carbon and nitrogen sources while also acting as inducers of laccase synthesis in white-rot fungi. Examples include wheat bran, rice bran, rice straw, corn straw, sugarcane bagasse, coffee husk, coconut shell, sawdust, pulp and paper mill wastes, spent mushroom substrate, and various fruit and vegetable residues. Leveraging such substrates not only reduces production costs but also supports the concept of “green catalysts,” promoting cleaner and more sustainable industrial processes [72,73].

In this context, laccases are considered primarily as fungal enzymatic biorecognition elements, while the nanomaterials discussed in this section act as exogenous electrode supports; nanomaterials biosynthesized or enabled by fungi themselves are addressed separately in Section 4. Electrochemical laccase-based biosensors have consistently demonstrated strong analytical performance across a wide range of targets, including phenolic compounds, catecholamines, endocrine disruptors, and environmental pollutants [74,75,76,77,78,79,80,81,82,83,84,85,86,87,88,89,90,91]. A common thread across the literature on the recent development of electrochemical laccase-based biosensors (Table 2) is that improvements in sensitivity, linear range, and operational stability are primarily driven by engineering of the electrode interface.

Across studies, sensor performance appears to be primarily governed by electrode architecture, enzyme source, and immobilization strategy rather than by the specific analyte. Carbon-based nanomaterials and two-dimensional supports—such as graphene, carbon nanotubes, carbon nanofibers, and MoS_2_-derived structures—are widely employed to increase electroactive surface area and conductivity, thereby enhancing enzyme loading and electron-transfer efficiency without altering the biological identity or catalytic role of the fungal enzyme itself [74,75,80,81,82,83,84]. Metallic and metal-oxide nanostructures provide a complementary design strategy by promoting oriented enzyme immobilization and improving catalytic kinetics. Approaches based on Au, ZnO, ZrO_2_, and magnetic nanocomposites illustrate how controlled nanostructuring can markedly improve sensitivity and, in some cases, reproducibility by increasing the effective active surface and stabilizing the enzyme–electrode interface [76,77,78,82]. Hybrid organic–inorganic matrices further extend this concept by combining conductive fillers with biocompatible stabilizers, resulting in improved operational stability and tolerance toward complex matrices such as river water, phytoproducts, and biological fluids [79,80,81,86,88].

An important design trade-off emerges when comparing MET and DET configurations. MET-based biosensors typically achieve lower detection limits and higher sensitivity but rely on additional redox components, increasing system complexity and the potential for interference [84,90]. In contrast, DET-oriented architectures offer simpler and more robust sensor designs with reduced background contributions, although they require precise control over enzyme orientation and electrode nanostructure to achieve efficient electron transfer [74,82].

Overall, fungal laccase-based biosensors benefit from high catalytic versatility and compatibility with nanostructured electrodes, particularly when laccases are derived from *Trametes* species, which provide robust activity and stability under immobilized conditions [74,79,82,87]. However, challenges remain in terms of long-term reproducibility, matrix-induced interference, and standardization of fabrication and validation protocols [88,89]. Addressing these limitations—through improved immobilization control, antifouling strategies, and reproducible electrode manufacturing—will be essential for translating laboratory-scale performance into reliable biosensing platforms for environmental, food, and biomedical applications.

Electrochemical biosensors based on fungal laccases represent the most extensively developed and analytically mature class of fungal enzyme-based sensing platforms [92]. For this reason, their analytical performance can be meaningfully contextualized within the broader landscape of electrochemical sensors developed for phenolic and related redox-active analytes. In parallel with enzymatic approaches, a substantial body of literature has focused on non-enzymatic electrochemical sensors based on nanostructured electrodes, including carbon nanomaterials, metal and metal-oxide catalysts, metal–organic frameworks, and hybrid composites, as summarized by Gu et al. [93]. In these systems, analyte detection is typically achieved through direct electrooxidation at the electrode surface, often facilitated by high electroactive surface area and enhanced charge-transfer kinetics. Non-enzymatic electrochemical sensors offer several practical advantages, including high chemical and thermal stability, long operational lifetimes, and reduced sensitivity to biological degradation, making them attractive for harsh environments and long-term deployment. However, direct electrooxidation mechanisms are inherently non-specific and frequently require higher operating potentials, which can increase susceptibility to interference and electrode fouling in complex matrices [93]. Although direct, one-to-one benchmarking remains intrinsically limited by the heterogeneity of experimental conditions, electrode architectures, and target analytes, a constrained quantitative comparison can be formulated when it is explicitly grounded in representative literature datasets. Based on the fungal laccase-based biosensors summarized in Table 2, limits of detection typically span from the nanomolar to micromolar range for environmentally relevant phenolic compounds, with reported values commonly between ~10^−9^ and 10^−6^ M, and linear dynamic ranges generally extending over two to four orders of magnitude. In these systems, enzymatic catalysis enables operation at relatively low working potentials, frequently at or below +0.4 V versus Ag/AgCl, which contributes to reduced background currents and mitigated electrode fouling in complex matrices. For non-enzymatic electrochemical sensors, recent comprehensive reviews focusing on advanced material-based platforms report comparable but not systematically superior analytical ranges. Carbon nanomaterial-, MOF-, COF-, MXene-, and TMD-based non-enzymatic sensors for phenolic contaminants predominantly achieve LOD in the nanomolar to micromolar domain, with representative values ranging from approximately 10^−9^ to 10^−6^ M across diverse analytes and electrode configurations. Linear dynamic ranges reported in these studies commonly extend over three to five orders of magnitude, reflecting the high electroactive surface area and catalytic properties of nanostructured electrodes. However, these non-enzymatic platforms typically rely on direct electrooxidation mechanisms and therefore operate at higher applied potentials, often exceeding +0.5 V versus Ag/AgCl, which increases susceptibility to interference and surface passivation in complex samples [93]. Taken together, the available literature does not support a systematic analytical performance advantage of non-enzymatic sensors over fungal laccase-based biosensors in terms of detection limits for phenolic compounds. Instead, both classes occupy largely overlapping sensitivity domains, while differing primarily in their transduction mechanisms and operational constraints. In this context, fungal laccase-based biosensors should not be viewed as direct competitors to non-enzymatic platforms, but rather as complementary analytical tools that combine biochemical selectivity with moderate operating potentials and compatibility with bio-derived and sustainable materials. While these platforms benefit from superior long-term stability and simplified fabrication, their reliance on direct electrooxidation increases susceptibility to matrix effects and electroactive interferents, particularly when sample pretreatment is limited. From a translational perspective, it is also important to distinguish laboratory-scale biosensors from established commercial electrochemical sensing solutions. Commercial platforms for phenolic detection and water-quality monitoring are predominantly non-enzymatic and prioritize robustness, standardization, and regulatory compliance, often relying on proprietary sensing chemistries and disposable electrode formats [93,94]. Non-enzymatic electrochemical sensors are actively developed to offer higher robustness and lower cost for on-site and in situ detection, especially when long shelf-life and minimal maintenance are required [93]. In contrast, biosensors employing enzymes—while highly selective—are often limited by poor operational stability, storage constraints, and fabrication complexity [93,95]. A recent review emphasized that biological recognition elements are particularly vulnerable to inactivation and degradation in real-world water samples [95]. Disposable, screen-printed electrodes (SPEs) and cartridge-based systems dominate the commercial landscape, as they allow simplified field operation and modular reagent replacement. These formats are compatible with quality assurance protocols and reproducible calibration, enabling their integration into regulatory workflows and industrial environmental monitoring [94]. In this context, fungal laccase-based biosensors should not be viewed as direct competitors, but rather as complementary platforms that feature biochemical selectivity, tunability, and compatibility with sustainable bio-derived materials. Their prominence in the literature and relative technological maturity justify their use as a reference case for positioning fungal enzyme-based biosensing within the broader electrochemical (bio)sensing landscape.

Photothermal and optical laccase-based biosensors (Table 3) represent a complementary class of enzymatic sensing platforms that extend laccase transduction beyond conventional electrochemical readouts by exploiting light–matter interactions, fluorescence modulation, and photothermally enhanced catalysis.

In these systems, nanostructured supports play a central role by acting simultaneously as enzyme carriers and optical or photothermal transducers. Photothermal strategies rely on nanomaterials capable of converting light into localized heat, thereby accelerating laccase kinetics and amplifying signal output. Nitrogen-doped carbon nanostructures and magnetic nanocomposites have been shown to enhance catalytic efficiency, shorten response times, and lower detection limits for phenolic compounds and neurotransmitters, while maintaining good performance in complex matrices such as environmental waters and synthetic urine [96,97]. In this context, photothermal activation provides a practical route to improve sensitivity without increasing system complexity or requiring additional labels. Optical laccase-based biosensors predominantly exploit fluorescence quenching, absorption changes, or enzyme-mediated biomineralization to enable label-free detection. Carbon-dot-based fluorescence probes integrated with fiber-optic or cellular imaging platforms offer high specificity, strong photostability, and excellent spatial resolution, making them particularly suitable for in vitro and cellular-level studies [98]. Colorimetric and microfluidic optical systems further demonstrate how laccase activity can be transduced into rapid, visually readable signals with good reproducibility and operational stability in food and beverage matrices [99,100]. Comparative analyses indicate that fluorescence-based optical biosensors excel in selectivity and spatial resolution but are less easily scalable, whereas photothermal and magnetically enhanced platforms provide faster kinetics, higher robustness, and broader applicability in real-sample analysis [96,97,98,99,100]. The integration of enzyme-compatible nanostructures with optical and photothermal readouts thus enables improved sensitivity and lower limits of detection while preserving biocompatibility. Looking forward, multi-modal platforms combining fluorescence, photothermal modulation, and microfluidic control—supported by rational sensor design and computational optimization—offer promising routes toward high-throughput, real-time enzymatic biosensing [101]. Across reported studies, fungal laccases derived primarily from *Trametes* species have proven particularly well suited to these photonic and microfluidic architectures due to their robust catalytic activity and stability under immobilized conditions [96,97,98,99,100].

Although laccases have been extensively studied in electrochemical biosensing, their optical properties have received comparatively less attention. Owing to their copper-containing active sites, laccases display well-defined and information-rich optical features, including absorption peaks around 600 nm corresponding to the T1 copper center in blue laccases, intrinsic fluorescence with emission near 440 nm upon excitation at approximately 330 nm, and Raman-active vibrations in the 350–450 cm^−1^ range. These spectral signatures reflect differences in enzyme structure and local microenvironment, which depend on molecular origin and surrounding conditions, thereby providing a measurable optical fingerprint of enzyme state. When properly controlled, such sensitivity enables optical transduction of enzyme–substrate interactions and functional state changes, supporting their application in optical biosensing. A recent review by Conigliaro et al. highlighted the exploitation of laccase optical properties in biosensors, particularly for the detection and quantification of phenolic compounds, and provided an overview of optical strategies applicable to environmental, food, and biomedical analysis [102].

Building on this conceptual framework, Wang et al. conducted a combined bioinformatics and enzymatic investigation to evaluate the suitability of a laccase from *Trametes* sp. SQ1 for optical biosensing applications. Rather than developing a complete sensing device, the study focused on characterizing molecular and functional properties relevant to signal generation and stability. The laccase exhibited exceptional robustness, retaining more than 150% of its initial activity after 96 h under various storage conditions, and its activity remained unaffected by repeated freeze–thaw cycles. Bioinformatic analysis predicted N-glycosylation sites and potential intermolecular disulfide bonds, features likely contributing to structural stability and oligomerization behavior. Optical characterization revealed distinct differences in absorbance at 400 nm and fluorescence intensity between oxidized and reduced states, indicating that redox-dependent optical modulation could serve as a viable sensing readout. In addition, optimization of fungal growth and enzyme-production media provided practical insights relevant to scalable biosensor development [103]. Another study by Wang et al. investigated the intrinsic optical properties of laccase from *Agaricus bisporus* during substrate-driven polymer formation. By combining theoretical modeling with real-time spectroscopic measurements, the authors monitored enzymatic polymerization and observed the emergence of a new absorption band at approximately 450 nm, which correlated directly with reaction progression. Concurrently, a decrease in fluorescence intensity was detected, reflecting changes in the enzyme’s optical behavior associated with enzyme–substrate complex formation and aggregation. These observations established a direct link between optical signal evolution and enzymatic activity, providing a mechanistic basis for real-time optical monitoring of laccase-catalyzed processes [104].

More recently, Biswas et al. reconstructed the molecular mechanism of catechol binding to laccase from *Trametes versicolor* using advanced computational approaches integrating crystallographic data and ligand structures from public databases. The study identified key residues and conformational dynamics governing substrate recognition and binding, offering atomistic insight into laccase–catechol interactions. Such mechanistic information is directly relevant to biosensing, as it can inform the rational engineering of laccase variants with improved binding efficiency, faster response kinetics, and enhanced stability. Moreover, elucidation of binding pathways and energetic landscapes provided a useful framework for optimizing enzyme immobilization strategies and signal reliability in optical biosensor platforms [101].

Collectively, these studies demonstrate that the optical properties of fungal laccases represent actionable transduction mechanisms rather than purely spectroscopic phenomena. By linking intrinsic optical signals to enzymatic function, molecular structure, and substrate interactions, optical laccase-based approaches expand the design space of fungal biosensors and complement established electrochemical paradigms. At the interface between enzyme-centric biosensing and fungal nanotechnology lie enzyme-based nanomaterials, such as self-assembled laccase nanoparticles, which are discussed in detail in Section 4. This body of work, together with the emerging enzyme-based nanomaterial strategies discussed in Section 4, highlights how fundamental enzymatic characterization, when aligned with sensing-oriented design principles, can support the development of optical biosensing strategies capable of real-time, label-free monitoring of phenolic compounds and related analytes.

At the same time, the sensitivity of these optical readouts to enzyme structure and microenvironment underscores the importance of further optimizing laccase performance for biosensing applications. Accordingly, future improvements to fungal laccases are expected to focus on both advanced enzyme engineering and the development of alternative catalytic materials. Protein and genetic engineering are regarded as the most effective strategies for enhancing properties such as catalytic efficiency, stability, and expression yields. Recent studies have explored ways to exploit the genetic diversity of host organisms, such as yeast, and have used genomic and proteomic analyses to improve laccase production [105,106]. Another promising avenue is the rational design of laccase mimics or nanozymes, which offer potentially greater stability and lower production costs than natural enzymes. Although these artificial catalysts show considerable promise, further work is still required to clarify their design principles and optimize their performance for reliable biosensing applications [107].

Beyond laccase, other fungal oxidoreductases have been integrated into biosensing platforms, most notably tyrosinases and ligninolytic peroxidases, where their catalytic activity directly enables electrochemical signal generation. In electrochemical biosensor designs, these enzymes primarily act as biorecognition elements, generating electroactive products suitable for amperometric or voltammetric detection.

Tyrosinase, a copper-containing polyphenol oxidase commonly extracted from fungi such as *Agaricus bisporus* and *Lentinula edodes*, catalyzes the oxidation of phenolic substrates to o-quinones, which can be readily electrochemically reduced at electrode surfaces. This mechanism allows direct, mediator-free signal transduction and underpins many tyrosinase-based biosensors. Sýs et al. systematically evaluated the influence of immobilization strategy on the analytical performance of tyrosinase amperometric biosensors using dopamine and catechol as model analytes. Three electrode configurations were compared, revealing that amperometric detection provided high sensitivity with reduced enzyme consumption compared with optical methods. Covalent immobilization on gold electrodes yielded the best performance, characterized by lower apparent Michaelis constants and higher reaction rates than polymer- or carbon-based configurations. These results demonstrate that electrode architecture and immobilization chemistry are critical determinants of biosensor sensitivity and efficiency [108].

Ligninolytic enzymes such as manganese peroxidase (MnP) have likewise been applied to electrochemical biosensing, particularly for environmental monitoring. MnP-based biosensors immobilized on carbon felt electrodes enabled sensitive voltammetric detection of textile azo dyes, achieving low detection limits (10 µg L^−1^), broad linear ranges, and good reproducibility. Importantly, differential electrochemical responses reflected dye-specific effects on enzymatic activity, allowing functional discrimination of pollutants beyond simple concentration measurements. The biosensors showed strong operational stability, retaining most of their activity over extended storage periods, supporting their suitability for long-term environmental applications [109].

Overall, tyrosinase- and MnP-based biosensors illustrate how fungal oxidoreductases can be effectively integrated into rationally designed biosensing configurations, where enzyme immobilization and electrode architecture critically govern analytical performance. These systems highlight the feasibility of mediator-free electrochemical transduction and demonstrate practical potential for environmental and biomedical sensing, while also underscoring the need for optimized immobilization strategies to ensure robustness and reproducibility.

#### 3.1.2. Other Fungal Enzymes

Fungal enzymes beyond laccases have been explored as biorecognition elements in biosensors, primarily in cases where their catalytic specificity enables selective signal modulation rather than high-throughput detection. Among these, lipases, cellulolytic enzymes, and esterases have been evaluated in a limited number of proof-of-concept sensing architectures. Fungal lipase-based biosensors have been mainly implemented using inhibition or product-formation strategies, particularly for the detection of environmental contaminants. De Moura Barboza et al. developed an inhibition-based electrochemical biosensor using a microbial lipase from *Ceratobasidium* sp. immobilized on lamellar zinc hydroxynitrate decorated with gold nanoparticles and integrated into a carbon paste electrode. The device enabled square-wave voltammetric detection of the fungicide carbendazim over 10–100 µg L^−1^, with a detection limit of 3.13 µg L^−1^ and accurate quantification in real water samples, showing good agreement with LC–MS analysis [110]. This study illustrates how fungal lipases can function as selective biochemical gates in electrochemical sensing, although their application remains largely restricted to inhibition-based formats. Optical lipase-based sensing has also been demonstrated, albeit less frequently. Hasanah et al. reported a reflectometric biosensor employing *Candida antarctica* lipase B immobilized in a pectin hydrogel for triglyceride determination. The sensor achieved a linear range of 1–5 mM with a detection limit of 0.05 mM and good stability over 10 days, confirming the feasibility of fungal lipases in optical transduction schemes for clinical and food-related analyses [111]. Cellulolytic enzymes have been explored primarily within cascade biosensor architectures, where signal amplification arises from multi-enzyme coupling rather than from the intrinsic sensitivity of a single bioreceptor. In this context, Liu et al. developed a colorimetric cascade biosensor integrating fungal β-glucosidase with glucose oxidase and horseradish peroxidase for the detection of amygdalin. The system exhibited rapid response (<4 min), a low detection limit (0.18 µM), and reliable performance for food safety applications, highlighting the utility of fungal enzymes in multi-step sensing architectures rather than standalone detection [112]. Biosensors based on fungal esterases remain comparatively rare, yet they demonstrate the potential of fungal enzymes as sustainable alternatives to animal-derived bioreceptors. Hafiz et al. reported an inhibition-based amperometric biosensor using an esterase from *Rhizopus oryzae* for the detection of the organophosphate pesticide methyl parathion. Immobilization on nanostructured carbon electrodes enabled detection down to 0.01 ng L^−1^, supporting the feasibility of fungal esterases for ultra-trace pesticide monitoring, albeit with moderate linearity and dependence on inhibition kinetics [113].

Collectively, these examples indicate that fungal lipases, cellulases, and esterases currently occupy niche roles in biosensor development, primarily as selective biorecognition elements within inhibition-based or cascade systems. Their limited adoption compared with laccases reflects both narrower analyte scope and greater dependence on complex reaction schemes. Nevertheless, these studies demonstrate that fungal enzymes beyond laccases can contribute meaningfully to biosensing when integrated into appropriately designed transduction architectures, particularly for environmental monitoring and food-safety applications.

#### 3.1.3. The Use of Crude Fungal Extract

Leveraging crude fungal extracts as biorecognition elements has emerged as a cost-effective and practically attractive strategy for biosensor fabrication (Table 4).

By avoiding enzyme purification, crude extracts preserve the native enzymatic microenvironment while substantially simplifying sensor preparation, reducing cost, and maintaining analytical robustness.

Across the studies summarized in Table 4, crude fungal extracts consistently enabled stable and reproducible signal generation, particularly in electrochemical biosensing formats. *Agaricus bisporus* crude extract retained more than 95% of its enzymatic activity after nine months at −20 °C while maintaining substrate specificity comparable to purified tyrosinase, demonstrating that long-term stability can be achieved without extensive biochemical processing [114]. This finding underscores a key advantage of crude extracts: functional durability without purification-related complexity.

Electrochemical transduction predominated in extract-based biosensors. Differential pulse voltammetry platforms employing *Marasmiellus colocasiae* extracts achieved low micromolar detection limits (0.12–0.14 µM) and reproducible linear ranges for catechin and gallic acid, confirming that analytical sensitivity is not necessarily compromised by biochemical heterogeneity [115,118]. Similarly, amperometric sensors based on *Trametes pubescens* laccase self-encapsulated within conductive polypyrrole matrices enabled reliable catechol detection, highlighting the importance of enzyme–matrix interactions in supporting efficient electron transfer and operational stability [116].

Crude tissue homogenates represent a further step toward fabrication simplicity by acting as ready-to-use sensing layers. A carbon-paste electrode modified with *Clitocybe nebularis* homogenate achieved sensitive L-DOPA detection (LOD = 0.76 µM) with minimal signal variability (0.82% CV) and good operational repeatability, demonstrating that minimal biological processing can still yield analytically robust biosensors when electrode composition and loading are optimized [117].

**Figure 6:**
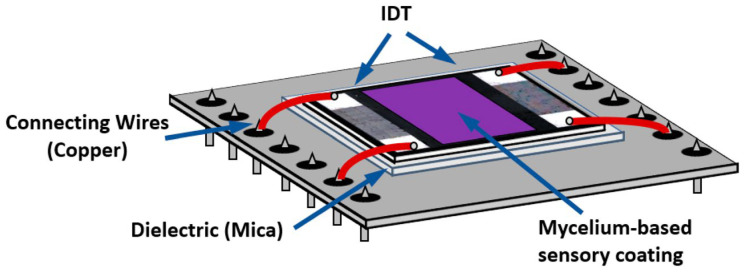
Components of a Mycelium Film-Based Acoustic Delay Line Sensor. Reproduced from Ref. [120].

Sensor performance was strongly influenced by fungal species selection and electrode architecture. Basidiomycetes rich in oxidative enzymes (e.g., *Trametes*, *Marasmiellus*, and *Ganoderma*) were particularly effective for phenolic detection in food and environmental matrices. Beyond electrochemical formats, crude extracts and mycelium-derived films from *Ganoderma* spp. integrated into surface and bulk acoustic wave devices (Figure 6) exhibited stable vapor responses over periods exceeding 60 days, extending the applicability of crude fungal materials to gas-phase sensing [119,120].

Analytical performance was further enhanced through coupling crude extracts with nanostructured conductive materials, including gold nanoparticles, ionic liquids, carbon nanotubes, and CNT/AuNP composites (Figure 6). These hybrid architectures consistently improved sensitivity and lowered detection limits, as demonstrated for bisphenol A (LOD = 0.03 µM) and aflatoxin M_1_ detection at ultratrace levels [121,122].

Remaining limitations primarily concern selectivity and batch-to-batch reproducibility, as crude extracts may contain multiple oxidative enzymes contributing to cross-reactivity. Nevertheless, studies employing controlled extraction protocols, enzymatic characterization, and optimized electrode formulations demonstrate that these challenges can be effectively managed [116,117].

Overall, crude fungal extracts and homogenates constitute analytically competitive, cost-effective, and application-ready biorecognition matrices. Their compatibility with diverse transduction mechanisms, robustness in real samples, and simplified fabrication align well with the performance and scalability requirements of contemporary biosensor platforms.

### 3.2. Non-Enzymatic Fungal Secretome Components in Biosensing

#### 3.2.1. Fungal Biosurfactants, Hydrophobins and Exopolysaccharides

Biosurfactants derived from bacteria and fungi are recognized as sustainable alternatives to synthetic surfactants in nanosynthesis. Owing to their amphiphilic nature and self-assembly properties, these molecules function as capping and reducing agents, facilitate encapsulation or templating processes, and serve as emulsifiers in nanoemulsion formation. Fungal biosurfactants often exhibit greater structural diversity than their bacterial counterparts, exemplified by the exclusive fungal production of several glycolipids—including sophorolipids, cellobiose lipids, and mannosylerythritol lipids, the most extensively studied yeast-derived biosurfactants—as well as unique surface-active proteins such as hydrophobins [123]. Although fungal biosurfactants can mediate nanoparticle synthesis with promising biomedical and environmental applications, their integration into sensor technologies remains comparatively underexplored [123,124]. Sophorolipids (SLs), for example, have been employed in the synthesis of copper nanowires, key components of transparent conducting electrodes (TCEs), which are essential in optoelectronics and increasingly relevant in multimodal biosensing [125]. Using an octadecylamine-mediated hydrothermal approach, Ranjana et al. demonstrated that adjusting the SL-to-ODA ratio precisely tuned copper nanostructure morphology—from ultra-long nanowires to micron-scale rods—highlighting the potential of sophorolipids for controlled nanomaterial engineering in sensing applications [126].

**Figure 7:**
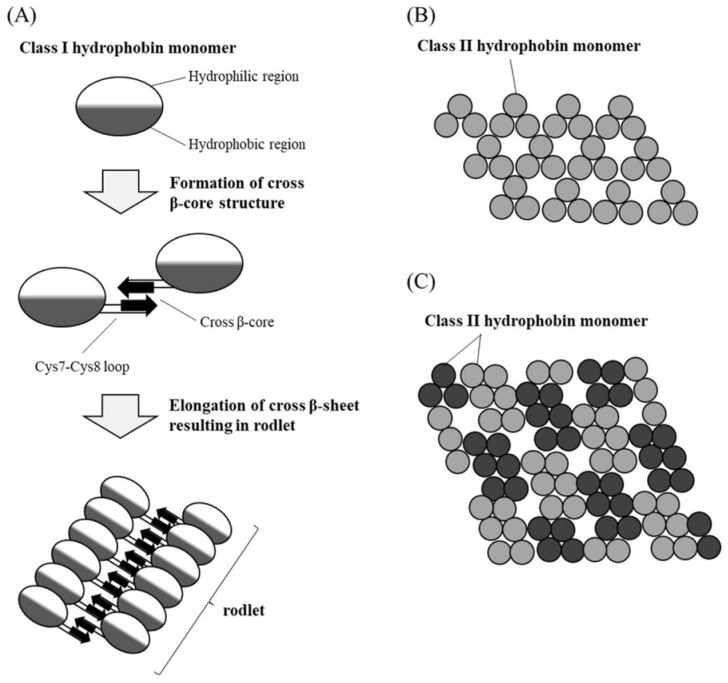
Schematic outlining Hydrophobin Class-Specific Self-Assembly. (**A**) Class I hydrophobins form rodlets. Light and dark regions indicate hydrophilic and hydrophobic surfaces, respectively. (**B**,**C**) Class II hydrophobins form defined oligomers consisting of either 3-molecule (**B**) or 6-molecule (**C**) units. Different shades represent distinct monomers within the oligomeric assembly. Reproduced from Ref. [128].

Among low-molecular-weight fungal biosurfactants, hydrophobins (HFBs) have attracted particular interest for biosensing because of their extraordinary surface-active behavior. These small, amphiphilic extracellular proteins spontaneously assemble at hydrophobic–hydrophilic interfaces, forming organized films on cell walls, air–liquid interfaces, or solid substrates. Predominantly produced by filamentous fungi, HFBs are among the most potent known biological surfactants and play essential roles in sporulation, aerial mycelium formation, spore protection, adhesion, host colonization, and morphogenesis. HFBs are traditionally classified into two groups—Class I and Class II—based on molecular size, amino acid composition, and aggregate solubility (Figure 7). Class I HFBs, produced by both Ascomycota and Basidiomycota, form highly stable amyloid-like rodlet films that require harsh acidic conditions for dissociation. Class II HFBs, mostly from Ascomycota, form less robust aggregates that readily dissolve in dilute solvents. Despite limited sequence identity, both classes share a conserved eight-cysteine motif [127].

Variants with altered disulfide-bond patterns and additional cysteine residues, produced by *Aspergillus* species, have been proposed as Class III hydrophobins [128], while atypical, pseudo-Class I-like HFBs identified in *Trichoderma* species define a new subclass with distinct structural and evolutionary features [129].

HFBs are characterized by their ability to form biocompatible, amphipathic films at diverse interfaces—including liquid–oil, liquid–liquid, air–liquid, and liquid–solid boundaries—where they stabilize emulsions and create protective surface layers. Among them, Class I HFBs have attracted particular interest for emulsion stabilization and surface modification, driving significant research and industrial engagement [130]. These distinctive surface-active properties have expanded the relevance of HFBs across multiple fields, including food biotechnology, surface engineering, and the development of fusion protein-based biosensors. Recent advances in the application of fungal HFBs to biosensing are summarized in Table 5.

**Figure 8:**
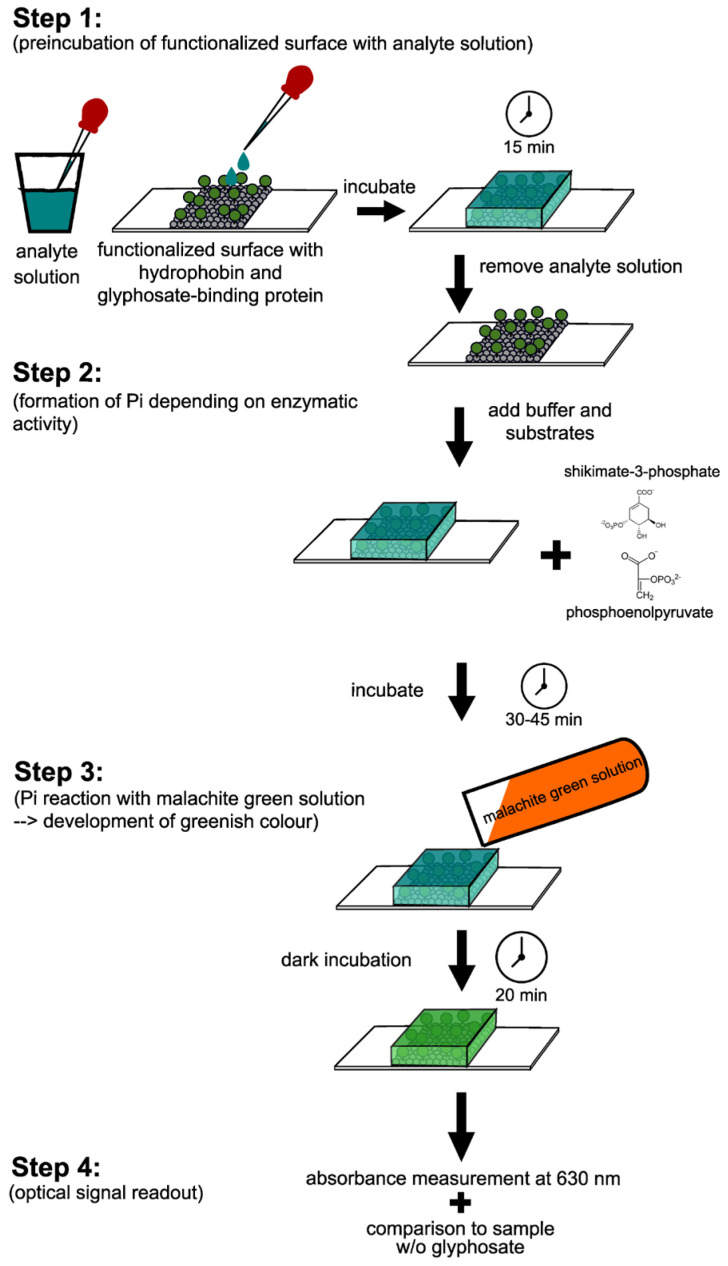
Workflow scheme of the proposed glyphosate detection assay based on functionalized surfaces and the malachite green assay. Reproduced from Ref. [132].

Collectively, these studies demonstrate that HFBs constitute versatile biointerface elements that operate at the critical junction between biorecognition and signal transduction. Their intrinsic self-assembly, amphiphilicity, and strong surface adhesion enable spontaneous, oriented immobilization of functional biomolecules on a wide range of substrates without chemical activation, supporting robust, reusable, and low-fouling sensing architectures. At the biorecognition–interface level, genetic fusion of HFBs with enzymes, antibodies, or binding peptides provides precise molecular orientation while preserving biological activity. For example, HGFI from *Grifola frondosa* enabled the construction of an ultrasensitive thrombin biosensor based on a rationally designed trifunctional fusion protein incorporating a far-red fluorescent reporter, achieving an outstanding limit of detection of 0.2 aM in serum while minimizing autofluorescence background [131]. Similarly, Ccg2 from *Neurospora crassa* was fused to 5-enolpyruvylshikimate-3-phosphate synthase to create glyphosate biosensors, yielding detection limits of 50 nM in surface inhibition assays [132] (Figure 8) and picomolar sensitivity in competitive optical particle-based platforms [133].

At the transduction level, HFB-functionalized surfaces have been successfully integrated into optical, electrochemical, and acoustic sensing platforms. HFBI from *Trichoderma reesei* was employed to functionalize film bulk acoustic wave resonators, enabling polarity-sensitive detection of volatile organic compounds with 2–8-fold signal enhancement compared to unmodified devices [134]. These results highlight how hydrophobin-driven film formation and surface charge distribution directly influence adsorption selectivity and transduction efficiency. In electrochemical biosensing, laccase–hydrophobin chimeras derived from *Pleurotus ostreatus* (POXA1b–Vmh2) and *Volvariella volvacea* supported efficient enzyme self-immobilization on graphene- and carbon-nanotube-based electrodes. These systems delivered broad linear ranges (µM–mM) and micromolar to sub-micromolar detection limits for phenolic compounds and neurotransmitters, while maintaining operational stability and reproducibility [132,133,134]. Here, the hydrophobin domain plays a central role in preserving enzyme orientation and facilitating effective electron transfer. Beyond enzymatic sensing, HFB-based fusion constructs have enabled selective detection of inorganic toxins and whole cells. Hydrophobins genetically fused to arsenate reductase or histidine-rich metal-binding peptides enabled fluorescence and electrochemical detection of arsenic and mercury at sub-nanomolar levels, combining enhanced sensitivity with reusability and storage stability [138,139]. Vmh2 from *P. ostreatus* has also been used to immobilize single-chain antibody fragments for marine neurotoxin sensing, achieving detection limits of 1.7 pg mL^−1^ for saxitoxin and 0.35 ng mL^−1^ for domoic acid [140].

More recently, HFB-enabled nano-biosensing platforms have been extended to user-friendly, data-driven detection schemes. Vmh2-based chimeras functionalized on gold nanoparticles enabled rapid colorimetric detection of *Escherichia coli* and *Staphylococcus epidermidis* down to 10 CFU mL^−1^. Importantly, integration with smartphone imaging and machine-learning algorithms allowed real-time quantification and on-site analysis, demonstrating how HFBs facilitate the transition from laboratory biosensors to deployable sensing systems [141]. In addition to their role in active sensing, hydrophobins show promise as anti-fouling and signal-stabilizing coatings. Klatt et al. demonstrated that recombinant H*Protein B coatings on cyclic olefin copolymer microfluidic chips achieved ~90% protein recovery over long channel lengths and maintained performance for up to eight weeks, significantly outperforming uncoated devices [142]. Such properties are directly relevant for biosensor longevity and reproducibility in complex matrices.

Overall, fungal hydrophobins function as molecular integrators that bridge biological recognition, surface functionalization, and signal transduction within coherent biosensor architectures. Their ability to promote controlled molecular orientation, suppress nonspecific adsorption, and stabilize active biomolecules enables reliable signal generation across diverse sensing modalities. Consequently, hydrophobins support the rational design of adaptable biosensors suitable for both environmental monitoring and biomedical applications.

At the same time, the diversity of sensing strategies demonstrated to date likely represents only a fraction of the functional potential offered by hydrophobins. While existing studies clearly establish their versatility in coupling molecular recognition to measurable outputs, further advances will depend on expanding the repertoire of available hydrophobins and systematically exploring structure–function relationships that govern selectivity, stability, and integration into portable, scalable, and high-throughput sensing platforms.

In this context, identifying novel hydrophobins and investigating hydrophobin families across a broader diversity of fungi represent key future directions. Advances in gene discovery enabled by next-generation sequencing, genomics, metagenomics, and bioinformatics have revealed substantial ecological, evolutionary, and functional diversity among hydrophobins. Notably, non-pathogenic fungi frequently harbor a greater number of HFB genes than pathogenic species, suggesting lifestyle-specific evolutionary trade-offs [143,144]. This functional plasticity spans both marine and terrestrial fungi, including the seaweed saprophyte *Paradendryphiella salina* [145], white-rot basidiomycetes such as *Rigidoporus microporus* [146] and *Coriolopsis trogii* [147], and widely cultivated edible mushrooms such as *Pleurotus ostreatus* [148,149] and *Pleurotus floridanus* [150]. Additional hydrophobins have been characterized in phytopathogenic fungi (*Fusarium graminearum* [151], *Penicillium expansum* [152]), the medicinal entomopathogen *Cordyceps militaris* [153], plant growth-promoting species such as *Trichoderma guizhouense* [154], endophytes including *Clonostachys solani* [155], saprophytes such as *Agrocybe cylindracea* [156], polypore mushrooms including *Grifola frondosa* [157,158] and *Funalia trogii* [159], and the alkaliphilic fungus *Sodiomyces alkalinus* [160]. Collectively, these examples highlight the widespread distribution and remarkable functional diversity of hydrophobins across fungal taxa, underscoring their largely untapped potential for next-generation biosensing applications.

Fungi secrete high-molecular-weight polymers known as exopolysaccharides (EPS), with sizes reaching up to 500 kDa. EPS may occur as loosely bound layers on the fungal cell wall—forming capsules or slimes—or as soluble molecules released into the surrounding growth medium. They exhibit considerable chemical heterogeneity, varying in constituent monosaccharides (e.g., glucose, galactose, mannose, fucose, xylose, and glucuronic acid), in the presence of non-carbohydrate modifications (such as acetyl, methyl, sulfate, and phosphate groups), and in overall molecular size, branching architecture, and glycosidic linkages. This inherent structural diversity underlies the broad range of physicochemical and biochemical properties exhibited by EPS [161]. As a consequence of this structural and chemical versatility, fungal exopolysaccharides have emerged as multifunctional biomaterials for biosensor engineering, offering intrinsic biocompatibility, biodegradability, and chemical tunability. Their diverse molecular architectures—from linear α-glucans to highly branched β-glucans—enable versatile roles in enzyme immobilization, nanoparticle stabilization, and signal transduction. These characteristics make EPS suitable for numerous applications, particularly in drug delivery and food processing. Among fungal EPS, pullulan represents a well-characterized example. Pullulan, a linear α-(1 → 6)-linked maltotriose polymer, is primarily produced by the black yeast-like fungus *Aureobasidium pullulans*. In addition to *Aureobasidium* species, commercial pullulan production also employs other Ascomycota fungi such as *Cytaria* spp., *Cryphonectria parasitica*, *Teloschistes flavicans* (the golden hair lichen), and the mitosporic fungus *Rhodotorula bacarum* [162]. Owing to its water solubility, oxygen-impermeable structure, and mechanical flexibility, pullulan is particularly well suited for visual and halochromic biosensors. Representative examples include β-lactoglobulin–pullulan composite films developed for food freshness monitoring, which provide a green, non-toxic sensing matrix capable of tracking pH variations during fish spoilage [163]. Although pure pullulan films can be brittle and costly to produce, blending with proteins or other polysaccharides markedly improves film-forming ability, durability, and optical responsiveness, thereby broadening its applicability in colorimetric and visual sensing formats.

Hyperbranched fungal EPS such as lentinan and schizophyllan offer additional functional advantages due to their β-(1,3)/(1,6) architectures. Lentinan, a β-glucan polysaccharide primarily derived from the shiitake mushroom (*Lentinula edodes*), consists of a β-(1,3)-glucan backbone with β-(1,6) branches and has recently attracted attention as a stabilizing scaffold for nanozyme-based biosensors. Over the past five years, lentinan-stabilized metallic nanostructures have been developed that exhibit enzyme-mimicking catalytic activity suitable for colorimetric sensing. Pd–LNT nanoparticles and Pt–LNT nanoclusters enabled peroxidase-like activity for glucose detection with low detection limits, while bimetallic PdPt_3_–LNT dendritic nanoparticles showed enhanced oxidase-like activity for L-cysteine sensing [164,165,166]. In these systems, lentinan acts not only as a green synthesis agent but also as a structural modulator that enhances nanoparticle dispersion, catalytic efficiency, and operational stability. Similarly, schizophyllan—a β-(1 → 3)-D-glucan with a triple-helix conformation isolated from *Schizophyllum commune*—has been proposed as a promising biomimetic matrix for biosensor interfaces, owing to its tunable mechanical properties and chemical functionality [167].

Botryosphaeran represents one of the most extensively investigated fungal EPS for biosensing applications. Secreted by fungi such as *Botryosphaeria*, this β-(1 → 3)(1 → 6)-D-glucan exhibits a gel-like, biocompatible structure that is particularly effective as an immobilization matrix for bioactive molecules. Its carboxymethylated derivative, carboxymethyl-botryosphaeran (CMB), further expands its utility by introducing tunable –CH_2_–COOH functionalities, primarily at the C-6 position. These carboxyl groups enhance hydrophilicity and solubility, provide anchoring sites for enzyme immobilization, and enable coordination with metal ions, nanomaterial stabilization, and hydrogel formation. As a result, both botryosphaeran and CMB have been widely employed in electrochemical biosensors, where they improve enzyme orientation, stability, and electron-transfer efficiency. Laccase–botryosphaeran platforms have achieved low micromolar to nanomolar detection limits for phenolic compounds, dopamine, and quercetin, while CMB-modified electrodes combined with carbon black or carbon nanotubes have enabled simultaneous multi-analyte detection with high reproducibility and long-term stability [79,168,169,170,171,172].

Research on the integration of fungal EPS into biosensor platforms is ongoing, with increasing interest in polysaccharides such as pullulan, lentinan, chitin/chitosan, and schizophyllan for biosensing applications. An overview of recent applications of fungal EPS in biosensing is provided in Table 6.

Overall, these studies demonstrate that the structural diversity of fungal EPS enables fine control over biosensor performance. Linear polymers such as pullulan are particularly effective in film-based visual and halochromic sensors, whereas branched β-glucans such as lentinan and botryosphaeran provide superior enzyme stabilization and catalytic enhancement in electrochemical and nanozyme-assisted platforms. Chemical modification strategies, including carboxymethylation and hybridization with conductive nanomaterials, further extend the functional range of fungal EPS, supporting the development of sensitive, selective, and robust biosensing systems for environmental monitoring, biomedical diagnostics, and food safety applications.

#### 3.2.2. Fungal Binding Proteins: Lectins and Aegerolysin

Lectins are non-catalytic, glycan-binding proteins that specifically recognize carbohydrate epitopes and play key roles in fungal ecology, including cell–cell interactions, adhesion processes, and defense mechanisms against predators [173]. Their ability to bind defined carbohydrate motifs with high affinity and specificity makes lectins particularly attractive as biorecognition elements for affinity-based biosensors. Fungal lectins display remarkable structural and functional diversity, encompassing proteins with distinct glycan specificities, oligomeric states, and environmental stability profiles.

Recent advances in computational analysis have substantially improved our understanding and exploitation of lectin–glycan interactions. Bojar et al. combined machine-learning approaches with expert annotation to systematically decode lectin binding specificities, overcoming long-standing challenges posed by glycan heterogeneity and non-linear recognition patterns. This strategy enabled the extraction of interpretable binding rules and provided a robust framework for rational lectin selection and biosensor design [174].

In parallel with carbohydrate-binding lectins, fungal aegerolysins constitute a distinct family of non-enzymatic binding proteins that selectively recognize specific membrane lipids or sterol-enriched lipid domains. Although non-toxic when expressed alone, aegerolysins exhibit high affinity for defined lipid species and have been widely employed as molecular probes for labeling and interrogating membrane microdomains. Their small size, intrinsic stability, and compatibility with immobilization or fusion strategies make them promising biorecognition elements for optical, electrochemical, and surface-based biosensing platforms [175].

Recent applications of fungal binding proteins in biosensing, encompassing both lectins and aegerolysins, are summarized in Table 7, which highlights their integration into electrochemical, optical, photoelectrochemical, and acoustic sensing formats.

**Figure 9:**
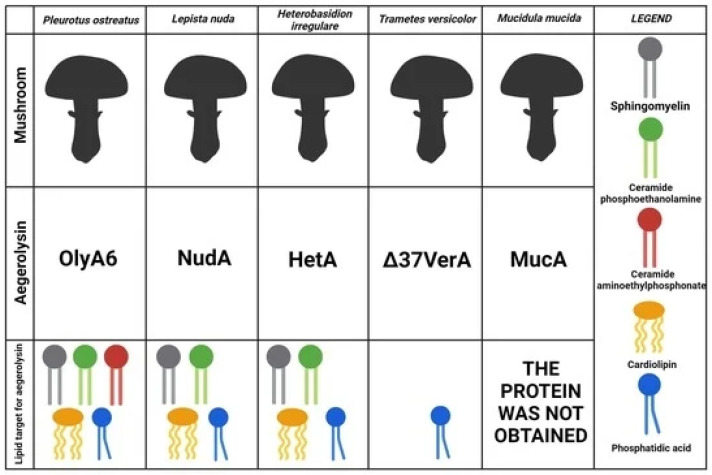
Overview of lipid-binding selectivity of fungal aegerolysins toward sphingolipids and glycerophospholipids. The figure illustrates the molecular recognition patterns that determine analyte specificity and form the basis for exploiting selected aegerolysins as lipid-specific biorecognition elements in biosensing and membrane-labeling applications. Reproduced from Ref. [177].

A representative example of lectin-based biosensing was reported by Abrantes-Coutinho et al., who developed an electrochemical glucose biosensor assembled with a lectin extracted from *Ganoderma applanatum*. In this work, machine-learning algorithms were employed to optimize immobilization parameters and improve signal interpretation. The resulting biosensor exhibited high sensitivity, strong operational stability across varying pH and temperature conditions, and accurate glucose quantification in pharmaceutical formulations. This study exemplifies how computational tools can be synergistically integrated with fungal lectins to enhance analytical performance and device robustness [176]. Beyond lectins, fungal aegerolysins have emerged as particularly powerful tools for lipid-focused biosensing. Aegerolysins selectively bind membrane lipids such as sphingomyelin, ceramide-phosphoethanolamine, and selected glycerophospholipids, enabling highly specific surface recognition. Importantly, recent studies have revealed that mushroom-derived aegerolysins bind not only to sphingolipids but also to glycerophospholipids—particularly phosphatidic acid and cardiolipin—without inducing pore formation. This non-lytic mode of interaction allows precise lipid recognition without membrane disruption, a key requirement for label-free and live-cell sensing applications. Aegerolysins derived from wood-decaying Basidiomycota fungi such as *Heterobasidion irregulare*, *Trametes versicolor*, *Mucidula mucida*, and *Lepista nuda* exhibit distinct and reproducible lipid-binding profiles. Figure 9 summarizes these lipid specificities, illustrating the molecular recognition patterns that underpin the use of selected aegerolysins as biorecognition elements for lipid-targeted biosensing.

Certain variants show pronounced specificity for phosphatidic acid under acidic conditions, functioning as molecular probes without causing membrane damage. This unique combination of selectivity, stability, and non-lytic behavior positions fungal aegerolysins as promising candidates for optical, surface-based, and electrochemical biosensors targeting membrane-associated biomarkers [177]. Taken together, lectins and aegerolysins represent complementary classes of fungal binding proteins that expand the scope of biosensing beyond enzyme-based catalysis. Lectins enable sensitive and robust detection of soluble carbohydrates, supporting applications in food quality control and pharmaceutical analysis. In contrast, aegerolysins extend fungal biosensing toward membrane lipid recognition, opening new opportunities in lipidomics, membrane biology, and live-cell labeling. Collectively, these proteins highlight the potential of fungal-derived affinity elements for designing biosensors based on precise molecular recognition rather than enzymatic signal amplification.

## 4. Myconanosynthesis: Fungal Biofabrication of Sustainable Nanomaterials for Biosensing

**Figure 10:**
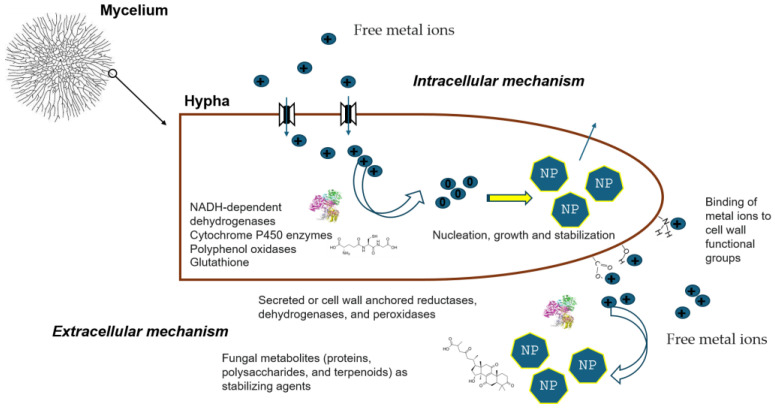
Schematic representation of extracellular and intracellular myconanosynthesis.

Nanomaterials, defined as materials with at least one dimension in the 1–100 nm range, possess unique size-dependent properties—including enhanced chemical, electrical, optical, thermal, mechanical, and surface characteristics—arising from their high surface-area-to-volume ratio. These features confer exceptional reactivity, sensitivity, stability, and strength, driving transformative advances in medicine, electronics, energy, and environmental science. Conventional synthesis methods, however, often require harsh chemicals, high energy input, and generate toxic byproducts, posing significant environmental and health risks. Biomediated synthesis, which employs microorganisms or biomolecules, offers a sustainable alternative by operating under mild conditions, reducing hazardous waste, and producing nanomaterials with improved biocompatibility and lower toxicity. Microbial-mediated approaches also enable scalable and cost-effective production with simplified purification, while reducing the use of hazardous reagents and energy-intensive conditions. By combining sustainability with high material performance, biomediated nanomaterials represent a critical pathway toward greener, safer, and more innovative nanomanufacturing processes. For further details, see recent reviews on green microbial nanobiosynthesis [30,178]. Among biogenic strategies, fungi have emerged as exceptionally effective agents for nanomaterial synthesis—a process often referred to as myconanosynthesis, mycosynthesis, or fungal-mediated nanoparticle fabrication. This method is widely recognized for its sustainability and scalability, providing a green and cost-efficient alternative to conventional chemical and physical techniques. Fungal systems are capable of producing a wide range of nanomaterials, including elemental nanoparticles (e.g., Ag, Au, Ag–Au bimetallic), metal oxide nanoparticles (e.g., ZnO, TiO_2_, Fe_3_O_4_), and carbon-based quantum dots (CQDs), each exhibiting distinct physicochemical and functional properties [31]. Biologically derived nanomaterials from fungi exhibit inherent catalytic, optical, and electronic functionalities, making them particularly attractive for integration into advanced biosensing platforms. Their natural surface coatings—resulting from proteins, polysaccharides, and metabolites in the fungal secretome—often enhance biocompatibility, stability, and selective reactivity, features essential for sensitive and robust analytical performance. Fungi mediate nanoparticle formation through a combination of biochemical and biomechanical processes. Although the precise molecular mechanisms remain incompletely understood, the fungal secretome plays a central role. Enzymes (e.g., reductases, oxidases), structural proteins, and secondary metabolites interact with metal-ion precursors, promoting their reduction and guiding nanoparticle nucleation. These secreted components also function as capping and stabilizing agents, preventing nanoparticle aggregation and imparting favorable physicochemical properties. Myconanosynthesis generally proceeds through two complementary “bottom-up” pathways: extracellular and intracellular biosynthesis (Figure 10). In extracellular biosynthesis, metal ions are reduced outside the fungal cell, within the culture medium. This reduction is facilitated by secreted enzymes such as nitrate reductases, laccases, and peroxidases, along with binding of metal ions to functional groups on the fungal cell wall—including hydroxyl, carboxyl, and amino groups. Extracellular proteins, polysaccharides, and other metabolites further stabilize the nanoparticles and help control their size and morphology.

Intracellular biosynthesis, by contrast, involves the uptake of metal ions into the fungal cytoplasm or vacuoles, where reduction is mediated by intracellular enzymes such as NADH-dependent dehydrogenases, cytochrome P450 enzymes, polyphenol oxidases, and glutathione. Glutathione, in particular, acts both as an electron donor and a stabilizing agent during the formation of metal nanoparticles such as silver and gold. Once reduced, metal ions nucleate and grow into nanoparticles that are subsequently stabilized by intracellular biomolecules. Several core biochemical mechanisms underpin both intracellular and extracellular mycosynthetic pathways. Reductive enzymatic activity, driven by NAD(P)H-dependent enzymes and nitrate/nitrite reductases, directly facilitates metal-ion reduction. Biosorption and complexation at the cell wall or within extracellular polymeric substances (e.g., EPS) provide nucleation sites through metal-binding functional groups. Metabolite-mediated processes, including the generation of carbonate or phosphate ligands via fungal metabolism (e.g., ureolysis), further influence nanoparticle formation. Reactive oxygen species (ROS) produced during fungal metabolic activity may also contribute to metal-ion reduction and modulate nanoparticle properties. Autolytic proteins, such as triosephosphate isomerase, have been shown to influence nanoparticle shape and stability. More broadly, proteins play multiple roles in mycosynthesis: they promote nucleation, prevent aggregation, stabilize nanoparticle surfaces, and facilitate metal coordination through functional groups such as thiols and carboxylates from amino acids like cysteine and glutamic acid. These extracellular and intracellular processes highlight fungal versatility in nanoparticle biosynthesis and underscore their potential for producing nanomaterials with controlled size, morphology, and functional properties suitable for biosensing and other high-value applications [31,32,178]. Filamentous fungi—particularly molds such as *Penicillium*, *Aspergillus*, and *Fusarium*—offer distinct advantages over bacterial systems for nanomaterial biosynthesis. These benefits include: (i) high tolerance to metal ions, (ii) an enhanced capacity for metal binding and accumulation, (iii) rapid biomass production under simple cultivation conditions, and (iv) efficient extracellular nanoparticle synthesis driven by secreted enzymes, reductive proteins, and secondary metabolites. Extracellular synthesis is especially advantageous because it avoids cell disruption, allows the fungal culture to be reused multiple times, and reduces the need for extensive downstream purification associated with intracellular nanoparticle recovery [30]. Myconanosynthesis by filamentous fungi—including species such as *Penicillium chrysogenum*, historically renowned for Alexander Fleming’s discovery of penicillin in 1928—has been extensively investigated over the past five years. The unique physicochemical characteristics of nanoparticles produced by these fungi indicate significant potential for future biomedical applications, ranging from drug delivery and imaging to biosensing and therapeutic interventions. Although relatively few studies have focused specifically on sensor-oriented applications, fungal-mediated nanomaterial synthesis has generated nanostructures with distinctive physicochemical and functional properties that pave the way for advanced biosensing and optoelectronic technologies (Table 8).

Filamentous fungi have been increasingly exploited as biofactories for the green synthesis of metallic and enzyme-based nanostructures with relevance to biosensing applications. In this context, fungi act as sustainable producers of functional nanomaterials rather than as active sensing elements. Species such as *Botrytis cinerea* and *Trichoderma* spp. have demonstrated the ability to generate gold and silver nanoparticles with controlled physicochemical properties, which can be integrated into electrochemical and optical sensing platforms. In these systems, fungal-derived nanostructures primarily function as signal-amplifying or catalytic interfaces within externally engineered sensing architectures.

Representative examples include plasmonic gold nanoparticles exhibiting strong SERS activity for optical biosensing [179] and enzyme-free electrochemical glucose sensing using biogenic Ag/Ag_2_O nanoparticles synthesized by *Fusarium oxysporum* [180]. Fungi have also enabled the production of bioinspired semiconductor and metal-oxide nanomaterials for fluorescence- and colorimetric-based detection [181,182], as well as enzyme-based nanomaterials, such as self-assembled laccase enzyme nanoparticles generated via fungal-derived biosynthetic routes and subsequently integrated onto electrochemical transducers for amperometric sensing [182,183]. Although largely proof-of-concept, these studies demonstrate the potential of fungal biosynthesis routes to yield nanomaterials with intrinsic sensing functionality while reducing reliance on complex chemical fabrication processes.

Collectively, fungal-mediated nanostructure synthesis provides a sustainable route to biosensor-relevant materials. However, this approach remains conceptually distinct from biosensing strategies that exploit the intrinsic electrical, optical, or adaptive behavior of living fungal materials, which constitute the primary focus of this review.

More recently, fungal biomass has been used to produce fluorescent carbon quantum dots (CQDs) with tunable photoluminescence, high aqueous stability, and abundant surface functional groups, enabling their use as optical sensing elements. In these systems, signal generation originates from the nanomaterial rather than from the living fungal matrix. A concise overview of representative fungal CQD-based sensing platforms is provided in Table 9.

**Figure 11:**
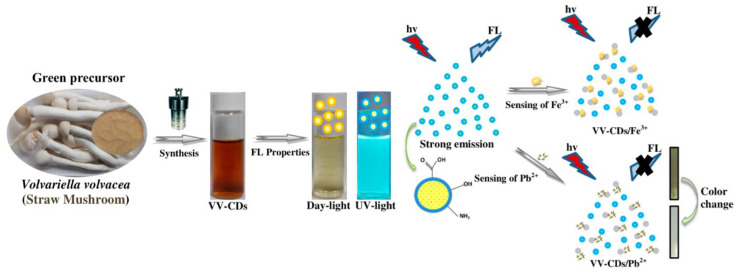
A schematic outlining of the preparation method of CDs synthesized from *Volvariella volvacea* (VV). The figure details the inherent fluorescence (FL) properties of the CDs and their practical application as a turn-off sensing platform for the sensitive detection of Fe^2+^ and Pb^2+^ ions. Reproduced from Ref. [187].

Within this framework, several representative examples illustrate how fungal CQDs can be integrated into functional biosensing platforms. A notable case involves the biosynthesis of ruthenium oxide quantum dots (RuO_2_ QDs) using *Fusarium oxysporum*, which enabled chromogen-free colorimetric detection of hydrogen peroxide with submicromolar sensitivity. Although based on an oxide quantum dot, this study provided an important proof of concept demonstrating that fungal systems can generate optically active nanodots suitable for direct sensing applications [185]. The majority of fungal CQD studies employ edible or medicinal mushrooms as carbon precursors, although yeasts and isolated fungal polysaccharides have also been explored. Hydrothermal synthesis remains the most widely adopted approach, enabling controlled formation of CQDs with tunable emission properties and surface chemistries dominated by oxygen- and nitrogen-containing functional groups, which strongly influence analyte recognition and fluorescence quenching mechanisms. Comparative studies on nitrophenol sensing highlight the critical role of precursor composition and heteroatom doping in determining analytical performance. CQDs derived from *Ganoderma lucidum* exhibited moderate quantum yields and detection limits for 2,4-dinitrophenol and 4-nitrophenol, whereas nitrogen and phosphorus co-doping significantly increased quantum yield and sensitivity, demonstrating that targeted chemical modification directly enhances fluorescence response and sensing capability [187]. Metal-ion detection represents another major application area for fungal CQDs. CQDs synthesized from *Volvariella volvacea* achieved one of the lowest reported detection limits for Pb^2+^ (LOD = 12 nM), while maintaining high selectivity and signal stability in aqueous systems (Figure 11) [187].

CQDs derived from *Pleurotus ostreatus* further demonstrated strong sensitivity toward multiple toxic metal ions, alongside antibacterial and anticancer activity, underscoring the multifunctional nature of fungal-derived carbon nanodots [188]. Highly selective Fe^3+^ sensing has also been achieved using mushroom-derived CQDs. Systems based on *Volvariella volvacea* and *Lentinus polychrous* provided low nanomolar detection limits, broad linear ranges, and robust photostability, enabling both solution-based and paper-supported sensing formats suitable for field-deployable applications [189]. Beyond whole fungal biomass, fungal polysaccharides have emerged as effective carbon sources for CQD synthesis. Carbon dots derived from *Poria cocos* polysaccharide enabled selective “on–off” fluorescence detection of Cr(VI), exhibiting strong blue emission, excellent tolerance to pH and ionic strength, and successful application in environmental and herbal samples [190]. Hybrid sensing platforms incorporating fungal CQDs have further expanded their analytical scope. Carbon dot–silver nanoparticle composites synthesized from *Pleurotus* species enabled simultaneous electrochemical detection of persistent organic pollutants, with CQDs contributing to nanoparticle stabilization and enhanced electron-transfer efficiency at the electrode interface [191]. These systems illustrate how fungal CQDs can function not only as optical probes but also as multifunctional components within integrated sensing architectures. Overall, these studies demonstrate that fungal-derived CQDs constitute a versatile, renewable, and highly tunable class of fluorescent nanomaterials for biosensing. By selecting appropriate fungal precursors and synthesis conditions, CQDs with tailored optical signatures, surface functionalities, and analyte affinities can be obtained. This intrinsic tunability, combined with low toxicity, biocompatibility, and environmental sustainability, positions fungal CQDs as promising candidates for next-generation sensing platforms in environmental monitoring, diagnostics, and multifunctional nanodevice engineering. Fungal nanosynthesis is not limited to metallic or carbon-based nanostructures but extends to a broad spectrum of biominerals, including oxides, carbonates, phosphates, sulfides, selenides, and tellurides composed of metals and metalloids such as Cu, Cd, Zn, Mn, Ni, Fe, Pb, Se, Te, and Ti. These nanominerals arise through interconnected biochemical and physicochemical mechanisms, including enzymatically mediated redox transformations, biomass-assisted nucleation, and metabolite-driven precipitation. For example, urea hydrolysis can generate carbonate ions that precipitate as metal carbonates (e.g., CdCO_3_, FeCO_3_, NiCO_3_), while secreted phosphates, sulfides, and amino acids act as ligands that promote mineral nucleation, growth, and stabilization processes. Two principal modes of fungal biomineralization have been described. Biologically induced mineralization (BIM) represents a passive process in which metabolic byproducts alter local chemical conditions, thereby triggering mineral precipitation. In contrast, biologically controlled mineralization (BCM) involves active regulation by the organism, enabling partial control over nucleation, particle size, morphology, and spatial localization [192]. In both cases, fungal cell-associated structures—particularly extracellular polymeric substances (EPS) and the cell wall—serve as physicochemical templates that concentrate ions and define nucleation sites. Enzymes such as nitrate and sulfate reductases further contribute by reducing metal ions to elemental or oxide forms, while protein conformational dynamics influence mineral architecture and stability. A wide range of mycogenic nanominerals has been documented, including metal oxides (e.g., TiO_2_, Fe_3_O_4_, Mn_2_O_3_, CeO_2_), carbonates (ZnCO_3_, NiCO_3_), phosphates (LiFePO_4_, Zn_3_(PO_4_)_2_), sulfides, selenides, tellurides, and complex composite materials. Fungal genera such as *Fusarium*, *Aspergillus*, *Penicillium*, *Neurospora*, *Rhizopus*, and *Phanerochaete* have been repeatedly implicated in these processes, underscoring fungi as versatile and sustainable biological platforms for nanomaterial fabrication. Although many of these nanominerals have been explored primarily from a materials-science or environmental perspective, their intrinsic catalytic and surface properties render them relevant to biosensing architectures. An emerging and complementary frontier within this context is the formation of nanozymes—nanomaterials that exhibit enzyme-like catalytic behavior. Nanozymes occupy an intermediate space between biological enzymes and inorganic catalysts, leveraging nanoscale dimensions, defect chemistry, and tunable surface states to mimic the activity of oxidoreductases such as peroxidases, oxidases, catalases, and superoxide dismutases. Compared with natural enzymes, nanozymes offer enhanced stability under harsh conditions, lower production costs, and the possibility of rationally tuning catalytic performance through structural or compositional control. Their catalytic repertoire has expanded beyond classical redox reactions to include hydrolytic and multifunctional activities, making them increasingly attractive for sensing applications [193]. Fungal biomineralization pathways provide a biologically driven route to nanozyme synthesis, enabling the generation of catalytically active nanostructures under mild and environmentally benign conditions. Although reports of fully integrated fungal-derived nanozyme biosensors remain limited, several studies have demonstrated catalytic behaviors that are directly relevant for signal generation and amplification in sensing systems. Yu et al. showed that *Trichoderma guizhouense* biotransformed hematite into ferrihydrite nanoparticles with pronounced peroxidase-like activity. Detailed surface analysis revealed that the catalytic behavior originated not from classical iron redox cycling but from an increased density of non-lattice oxygen species—likely hydroxyl groups associated with oxygen vacancies—which acted as catalytically active sites for hydrogen peroxide decomposition [194]. This surface-chemistry-driven mechanism enabled efficient generation of reactive intermediates and illustrates how fungal processing can endow inorganic nanomaterials with enzyme-like functionality. In a complementary approach, Mekonnen et al. developed a copper-based nanozyme stabilized by fungal-derived chitosan extracted from *Irpex* sp. In this system, the biopolymer scaffold prevented nanoparticle aggregation, facilitated electron transfer, and imparted laccase-like catalytic activity toward phenolic substrates. Although the primary application was environmental remediation, the combination of high catalytic efficiency, structural stability, and biopolymer-mediated control highlights clear potential for translation into electrochemical or colorimetric biosensing formats [195]. Collectively, these studies indicate that fungal biomineralization and biopolymer-assisted nanosynthesis can generate catalytically active nanomaterials that bridge biological function and inorganic robustness. Rather than representing mature biosensor platforms, fungal-derived nanominerals and nanozymes currently function as enabling materials that expand the design space of biosensors by providing stable, green, and tunable catalytic interfaces. Future progress will depend on coupling these materials with defined biorecognition elements, optimizing their integration with transducers, and systematically evaluating their analytical performance within complete sensing architectures. Nonetheless, the convergence of fungal biology, biomineralization, and nanozyme chemistry establishes a promising foundation for next-generation biosensor development. The structural complexity of fungal mycelia has been exploited as a living template for the controlled fabrication of hybrid nanomaterials with relevance to sensing technologies. The hierarchical porosity and three-dimensional organization of mycelial networks provide natural scaffolds that guide nanoparticle nucleation, growth, and spatial distribution. This bio-inspired, bottom-up strategy offers several advantages over conventional nanofabrication approaches, including reduced energy input, lower environmental impact, and intrinsic scalability. As a result, fungal–inorganic hybrids combine structural precision with functional versatility, positioning them as promising materials for advanced biosensing and spectroscopic detection. A seminal example was reported by Pal et al., who used *Trichoderma asperellum* and *Aspergillus sydowii* as biological templates to fabricate nanoparticle-assembled gold microtubes for surface-enhanced Raman scattering (SERS)-based molecular sensing [196]. In this approach, fungal spores were cultivated in colloidal gold solutions, enabling expanding mycelia to incorporate gold nanoparticles within their cell walls. Subsequent thermal treatment removed the organic matrix and partially fused the nanoparticles, yielding porous gold microtubes that faithfully replicated fungal morphology. These structures exhibited very high SERS enhancement factors, reaching ~1 × 10^10^ for methylene blue, and enabled ultrasensitive detection of model analytes including methylene blue, rhodamine 6G, methyl orange, and D-glucose. Although minor morphological heterogeneity resulted in local signal fluctuations, each individual microtube functioned as a robust and effective SERS substrate. Complementary insights into mycelium–nanoparticle interfaces were provided by Sadaf et al., who quantitatively investigated gold nanoparticle deposition on *Aspergillus niger* mycelia as a function of nanoparticle surface chemistry [197]. While this study did not directly implement sensing devices, it demonstrated that fungal viability, stress responses, and nanoparticle loading density are strongly modulated by surface functionalization. Glucose- and NaBH_4_-coated nanoparticles promoted dense deposition with minimal stress, whereas citrate-coated particles induced pronounced physiological responses. Importantly, this work established quantitative correlations between nanoparticle concentration and mycelial loading, providing design rules relevant for the rational engineering of reproducible mycelium-based sensing composites. Further advancing this concept, Maciel et al. employed multiple filamentous fungi—including *Aspergillus niger*, *Phialomyces macrosporus*, *Trichoderma* sp., *Penicillium* sp., and *Talaromyces pinophilus*—as biotemplates to fabricate gold microtubes via uniform nanoparticle deposition and high-temperature calcination [198]. The resulting structures preserved fungal morphology and exhibited substantially increased electrochemical surface area compared with flat gold electrodes. When evaluated as SERS substrates, these mycelium-derived microtubes enabled detection of rhodamine 6G at concentrations as low as 1 × 10^−8^ M, highlighting the role of hierarchical roughness and nanoscale curvature in signal amplification. Although tested with model analytes, these systems demonstrated the high sensitivity and structural fidelity achievable through fungal templating. The fungal-templating strategy has also been extended beyond noble metals. Malta et al. introduced the concept of “fungal cyborg cells” by integrating oxide nanoparticles into fungal cells to generate hollow transition-metal-oxide microtubes [199]. Using *Aspergillus niger* and *Phialomyces macrosporus* cultivated in suspensions containing TiO_2_ or WO_3_ particles, the authors produced dense inorganic coatings that retained fungal morphology after controlled calcination. Structural and spectroscopic analyses confirmed preservation of crystalline oxide phases and increased surface roughness. Although more fragile than gold replicas, these oxide microtubes demonstrated sufficient structural integrity to support potential sensing and related analytical applications. Collectively, these studies demonstrate that fungal mycelia can serve as versatile living templates for the fabrication of hierarchically structured inorganic micro- and nanomaterials with strong relevance to biosensing and spectroscopic detection. By exploiting the intrinsic architecture of fungal networks, it is possible to generate high-surface-area, porous structures that support efficient signal amplification without complex lithographic processing. While most demonstrations to date rely on model analytes, the robustness, scalability, and sensitivity of fungal-templated architectures underscore their potential as next-generation platforms for SERS-based sensing, electrochemical transduction, and hybrid analytical devices. Overall, these advances indicate that myconanosynthesis provides a sustainable and versatile route to nanomaterials with finely tunable physicochemical properties, directly enabling signal amplification, functional integration, and sensitivity enhancement in biosensing platforms. In this context, fungi emerge as powerful biofactories for next-generation biosensors, with additional relevance for catalytic and optoelectronic systems.

## 5. Mycelium- and Fungal-Derived Living Materials: Functional Properties and Applications

Fungi have gained increasing attention as biofabrication platforms for biosensing and bioelectronic applications through the development of mycelium-based composites (MBCs). Mycelium forms an interconnected, porous network that can be engineered to function as a selectively permeable, mechanically stable scaffold for sensor integration. When grown on lignocellulosic substrates, mycelium consolidates the material into lightweight composites whose microstructure can be tuned through strain selection, substrate composition, and post-growth processing. Importantly, these parameters directly influence material properties that are critical for biosensing, including analyte permeability, interfacial contact with electrodes, and signal transduction efficiency at the bio–electrode interface. Processing strategies such as controlled drying, hot pressing, or biochemical modulation of the growth medium enable tuning of mechanical stiffness, porosity, and surface morphology, thereby tailoring MBCs for integration with electrochemical and bioelectronic transducers. Recent studies have demonstrated that the hierarchical architecture of mycelial materials supports functional integration with conductive nanomaterials, enabling hybrid systems capable of transducing biological activity into measurable electrical signals [200,201,202]. Beyond serving as passive scaffolds, mycelial networks can exhibit stimulus-responsive electrical behaviors, supporting their use as active components in biosensing and bioelectronic systems.

### 5.1. Bioelectrical and Computational Properties of Mycelium Networks

Electrical signaling represents a fundamental mechanism by which living organisms integrate environmental information and coordinate internal processes. Although extensively characterized in plants and animals, its occurrence and functional significance in fungi have only recently begun to receive focused attention. Filamentous fungi expand via apical extension of tubular hyphae that interconnect to form modular and dynamic mycelial networks. These networks continuously reorganize in response to nutrient availability, interspecific interactions, and environmental stress. Their modular architecture confers resilience and adaptability but also necessitates coordination across spatially distributed regions—an integration that electrical signaling may help to mediate. Early studies provided evidence that filamentous fungi generate action-potential-like events and measurable electrical currents, particularly at actively growing hyphal tips. Although the precise biological functions of these signals—whether they facilitate intra- or intercellular communication—remain unresolved, advances in electrophysiology and renewed interest in mycorrhizal networks as potential mediators of interspecies information exchange have revitalized research in this area. Structurally, hyphae are well suited for electrical conduction: continuous plasma membranes maintain ionic gradients; cell walls enriched in hydrophobins, melanin, and polysaccharides provide partial insulation; and septal pores ensure cytoplasmic continuity while enabling rapid sealing following damage. These architectural features allow fungal networks to maintain conductivity even when hyphal segments are disrupted, supporting long-range signal propagation under variable environmental conditions. Owing to the presence of a rigid cell wall and the small diameter of hyphae (2–10 µm), intracellular electrophysiological recordings in fungi—such as patch-clamp and sharp microelectrode approaches—remain technically challenging and often require invasive strategies to access the plasma membrane. Early intracellular recordings using glass microelectrodes revealed fluctuating membrane potentials in species such as *Neurospora crassa* and *Armillaria bulbosa*, while subsequent studies employing extracellular vibrating electrodes detected oscillatory ion fluxes near the hyphal tips of *N. crassa* and *Schizophyllum commune*. Initially attributed to polarized growth, these electrical currents were later linked to nutrient transport and metabolic regulation. More recent experiments demonstrated that fungi respond to externally applied electric fields, exhibiting galvanotropism and electrotaxis in which hyphae reorient toward or away from electrodes depending on ionic composition and pH [203]. Over the past several years, studies have shown that fungi can generate action potential-like electrical currents, shifting the perception of mycelium from a passive structural matrix to a dynamic, signal-processing network. This electrophysiological activity provides an essential foundation for developing fungal-based sensing technologies. Research on the pink oyster mushroom (*Pleurotus djamor*) has established characteristic electrical profiles for fruiting bodies, revealing voltage fluctuations and rhythmic signaling patterns that likely coordinate growth and responses to environmental cues. Using subdermal needle electrodes, investigators identified two spontaneous modes of extracellular action potential-like impulses: a high-frequency mode with a periodicity of ~2.6 min, and a low-frequency mode recurring approximately every 14 min. The low-frequency spikes displayed higher amplitudes and repolarized at nearly twice the rate of high-frequency spikes. The fungi also responded to external stimuli, including ethanol, water, and heat. A brief thermal pulse elicited a delayed response in the directly stimulated sporocarp (~103 s), whereas neighboring, non-stimulated mushrooms within the same cluster reacted more rapidly—sometimes within 26 s—indicating coordinated intra-cluster signaling. Signal-processing analyses further quantified the electrical signatures of *P. djamor*, revealing spike durations averaging 402 s, amplitudes between 0.5 and 6 mV, and Kolmogorov complexity values ranging from 11 × 10^−4^ to 57 × 10^−4^. Such complexity metrics support the hypothesis that fungal electrical activity reflects purposeful information exchange rather than stochastic fluctuations [204,205]. Comparative studies in *Ganoderma resinaceum* revealed similar but distinct electrophysiological patterns. Extracellular recordings of its antler-like sporocarps showed singular spikes, compound spikes, and spike trains with amplitudes of 0.1–0.4 mV and spike widths of 300–500 s (5–8 min). These spike durations—roughly twice those observed in *P. djamor*—likely reflect the slower metabolic rate of *G. resinaceum*. Such species-specific differences demonstrate that electrical signaling is broadly conserved across fungal lineages, yet each species exhibits a unique electrophysiological “signature.” This variability underscores the potential for tailoring fungal electrical properties to specific sensing or computational applications [206]. Building on these findings, subsequent work began interpreting fungal electrical spiking as a language-like system. By analyzing the electrical activity of *Omphalotus nidiformis*, *Flammulina velutipes*, *Schizophyllum commune*, and *Cordyceps militaris*, researchers categorized individual spikes as “words” and sequences of spikes as “sentences,” suggesting that mycelial networks may encode and transmit information in structured, species-specific patterns. Linguistic and complexity analyses revealed that the distributions of fungal “word” lengths resembled those found in human languages in a statistical sense. For example, the average word length for *Cordyceps militaris* (4.7 spikes) was comparable to English (4.8 words), while *Schizophyllum commune* (4.4 spikes) closely matched Greek (4.45 words). Algorithmic and Lempel–Ziv complexity hierarchies further ruled out randomness, supporting the view that mycelial networks process information through interactions among electrical spikes in a manner analogous to neuronal systems, with species-specific spiking characteristics [207]. The computational potential of fungal electrical activity was investigated further using *Ganoderma lucidum* mycelium networks to evaluate frequency-modulated signal propagation. The mycelium reliably transmitted signals within the 100 Hz–10 kHz range, with recovered frequencies closely matching the inputs, and appropriate controls confirmed that conduction occurred through the mycelial network itself. Nonlinear autoregressive exogenous (NARX) modeling approximated signal transfer using a simple five-term first-order polynomial, accurately capturing the essential dynamics of the system. These results highlighted the potential of mycelium-based composites in analog electronics and unconventional computing, suggesting that input signal properties could be decoded and thereby providing a conceptual foundation for mycelium-based information-processing architectures [208]. Table 10 provides a concise overview of the studies described above, summarizing key findings on the electrical properties of fungi, including the species examined, recording methods employed, characteristic spike parameters, and the broader implications for sensing and information-processing applications.

**Figure 12:**
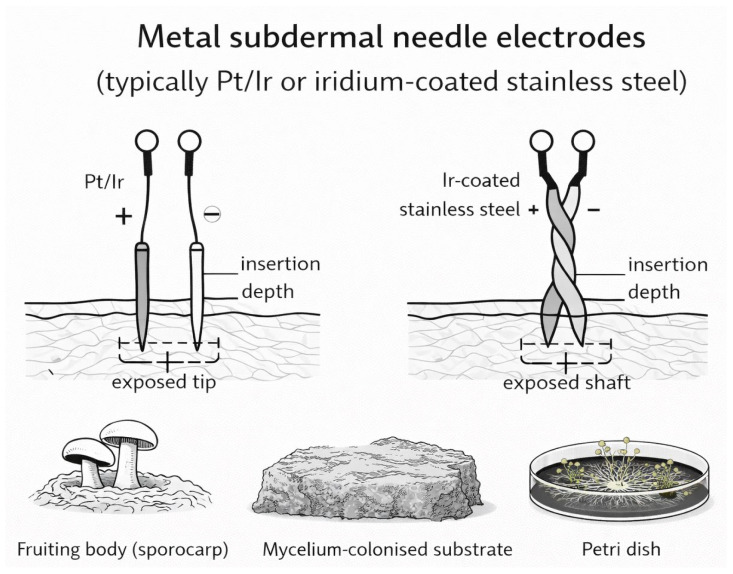
Schematic overview of metal subdermal needle electrodes and fungal substrates used for bioelectrical measurements. Differential and twisted-pair needle electrode configurations (see main text for material specifications) are shown with key features including exposed conductive regions, insertion depth, and inter-electrode spacing. Such electrodes can be applied to fruiting bodies (sporocarps), mycelium-colonised solid substrates, and Petri dishes with surface mycelial growth.

The body of evidence presented across these studies indicates that fungal networks display complex and structured bioelectrical activity, revealing mycelium as a dynamic and responsive biological system rather than a passive substrate. Emerging data suggest that interactions among electrical spikes may support forms of information encoding and transmission analogous to neuronal processes, highlighting the promise of fungal systems for applications in biosensing, analog electronics, and unconventional computing. Despite this progress, significant questions remain. Future investigations must elucidate the underlying biophysical mechanisms, determine how electrical signaling varies across fungal taxa, and distinguish species-specific characteristics from conserved electrophysiological features that may be fundamental across the fungal kingdom. As reported in Table 10, current investigations of fungal electrical activity have predominantly relied on extracellular electrode configurations, reflecting the physical constraints imposed by rigid cell walls and the narrow diameter of hyphae. Among these, metal subdermal needle electrodes (typically Pt/Ir or iridium-coated stainless steel), often arranged in differential or twisted-pair configurations (Figure 12), represent the most widely adopted and practically robust approach [204,205,206,207,208].

When inserted into sporocarps, mycelium-colonized solid substrates, or agar plates with surface mycelial growth, these electrodes enable stable, long-term recordings of network-level electrical dynamics, including spike trains, bursts, and slow potential fluctuations. However, because they capture extracellular field potentials rather than transmembrane voltages, the recorded signals generally exhibit low to medium signal-to-noise ratios, with amplitudes typically ranging from several tens of microvolts to a few millivolts and substantial variability across replicates. As summarized in the literature reviewed by Buffi et al. [203], intracellular microelectrode and patch-clamp approaches provide high signal fidelity under controlled conditions, whereas extracellular techniques—including vibrating microelectrodes, subdermal needle electrodes, and planar microelectrode arrays—are consistently associated with lower signal-to-noise ratios due to field-potential recording, limited electrode–cell coupling, and sensitivity to environmental noise. Subdermal needle electrodes therefore constitute a pragmatic intermediate solution, bypassing the need for direct membrane access while preserving tissue integrity. They are particularly well suited for investigating signal propagation, collective dynamics, and species-specific electrophysiological patterns at the scale of intact mycelial networks or fruiting bodies. At the same time, their limited spatial specificity and intrinsic signal-to-noise constraints render them insufficient for resolving intracellular mechanisms or ion-channel-level processes, underscoring their complementary role rather than a substitute for more invasive or higher-resolution techniques. Future progress in fungal electrophysiology is likely to rely on less invasive and more spatially resolved methodologies capable of circumventing these limitations. Promising directions include femtosecond laser-based nanosurgery to enable localized membrane access, high-impedance extracellular microelectrode arrays adapted for low-frequency signals, and microfluidic platforms that stabilize individual hyphae while minimizing mechanical disturbance. In parallel, optical strategies—such as voltage-sensitive dyes, genetically encoded ion or voltage indicators, and intracellular nanosensors—offer non-contact alternatives to classical electrode-based recordings. Together, these emerging methodologies may allow dynamic mapping of electrical activity across intact mycelial networks while preserving physiological integrity, addressing key limitations of traditional intracellular techniques [204].

### 5.2. Unconventional Computing and Bioelectronic Devices

Electrophysiological investigations have substantially advanced the understanding of fungal communication, demonstrating that mycelial networks generate action potential-like electrical impulses. These findings support the view that fungi function as dynamic, responsive, and communicative systems at the network level rather than passive biological substrates. Their intrinsic electrical activity, coupled with structural heterogeneity and adaptive growth, positions fungal mycelium as a promising platform for unconventional computing and emerging bioelectronics sensing technologies.

Foundational studies—most notably those conducted on the pink oyster mushroom *Pleurotus djamor*—established early conceptual links between fungal electrophysiology, information processing, and computation. These works proposed that electrical spikes propagating through mycelial networks can encode information-like patterns, with the spatially distributed hyphal architecture acting as a parallel processing medium. Experimental evidence supported this hypothesis by showing long-range signal transmission across spatially separated tissues: localized saline stimulation of a single fruiting body induced measurable electrical responses in neighboring, non-stimulated mushrooms. Discrete automata modeling further indicated that the topology of the mycelial network constrains the class of logical operations it can implement, providing early computational evidence for the conceptual feasibility of a ‘fungal computer’ [209].

Subsequent studies translated these theoretical concepts into physical implementations. Boolean logic was realized in living mycelium-based composites of *Pleurotus ostreatus*, where stimulation with sequences of 4-bit input strings produced logical operations including NAND, OR, and AND. These systems exhibited behaviors spanning multiple cellular-automaton complexity classes, including functions consistent with computational universality. However, output variability remained significant, reflecting the intrinsic metabolic dynamics and continuous structural reorganization of the living substrate [210]. Complementary theoretical work using two-dimensional fungal sandpile automata demonstrated computational universality through controlled compartmentalization, extending earlier one-dimensional models describing information flow mediated by hyphal pore dynamics [211,212]. In parallel, simulations of excitation wave propagation in *Aspergillus niger* colonies based on the FitzHugh–Nagumo model further supported the plausibility of Boolean logic encoded in fungal electrical signaling [213].

Beyond purely computational demonstrations, fungi have also been explored as passive or hybrid bioelectronic materials. Mycelium of *P. ostreatus* exhibited capacitance values two- to four-fold higher than uncolonized substrates, spanning from picofarads range to hundreds of microfarads. This behavior was attributed to complex dielectric and pseudocapacitive processes driven by ionic dynamics at hyphal membranes. Nevertheless, these systems remained highly sensitive to environmental conditions and suffered rapid degradation upon dehydration. They also displayed low quality factors and were vulnerable to electrolysis under elevated voltages [214]. Despite these limitations, *P. ostreatus* has continued to serve as a model organism for sensing, communication, and unconventional computing studies.

**Figure 13:**
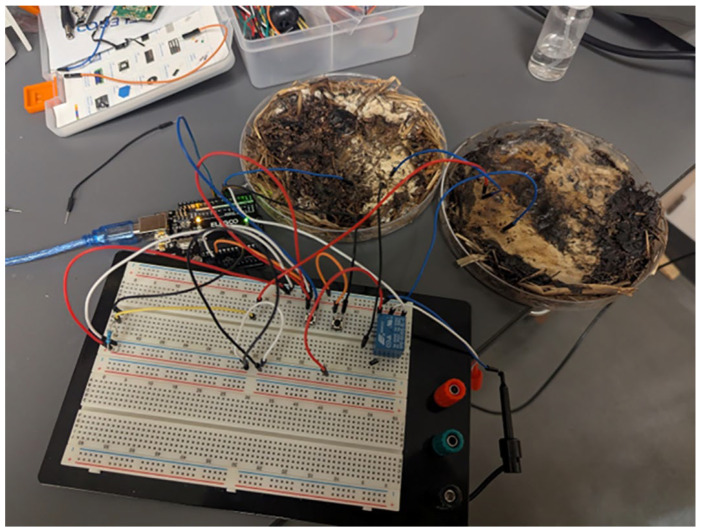
Wired fungal memristor samples based on shiitake mycelium, showing the experimental setup used for bioelectronic characterization. Electrode pairs were inserted into the mycelium substrates and connected to a breadboard-based electronic interface for the implementation and testing of volatile memory behavior in fungal memristive elements. Reproduced from LaRocco et al. [216] under the Creative Commons Attribution License (CC BY 4.0).

This potential was further supported by fungal photosensors constructed from *P. ostreatus* fruiting bodies, which converted changes in illumination into measurable electrical signals. Functionalization with PEDOT:PSS significantly amplified photoresponses, producing current spikes up to eightfold larger than those observed in untreated tissues, while simultaneously highlighting moisture content as a critical determinant of device stability [215]. Fungal materials have also been applied to advanced information-processing architectures. Mycelium of *Lentinula edodes* has been used to fabricate sustainable memristive devices exhibiting pinched hysteresis at 10 Hz and functioning as volatile memory elements up to 6000 Hz (Figure 13), further supporting the feasibility of fungal-based unconventional electronic components [216].

These systems offer low power consumption and straightforward fabrication; however, challenges related to miniaturization and long-term stability remain. Building on these properties, mycelium-based systems have been integrated into physical reservoir computing platforms. Morphologically tunable mycelium chips infused with PEDOT:PSS were shown to transform time-varying inputs into nonlinear, high-dimensional states, enabling tasks such as NARMA-10 prediction. Structural parameters—including branching density and hyphal connectivity—were found to directly influence charge transport and memory capacity [217]. Complementary cellular-automaton modeling demonstrated that mycelium-inspired networks exhibit small-world connectivity patterns known to enhance separability and memory, achieving classification accuracies up to 97.09% on the MNIST dataset [218]. Further advances were achieved through Memristive Oscillating Cellular Automata (MOCA), a low-power hardware architecture inspired by mycelial network dynamics. By integrating memristive elements into an oscillatory grid, MOCA enabled distributed state transitions, nonlinear propagation, and history-dependent behavior, achieving classification accuracies up to 99.3% on emotional-state datasets while operating at approximately 53 µW [219]. Related work employed reconfigurable memristive spiking grids to emulate fungal spike dynamics, generating controllable digital twins for studying interactions between synthetic and living substrates [220].

Collectively, these studies delineate a conceptual and technological bridge between unconventional computing and biosensing. In this framework, fungal mycelial networks are no longer viewed solely as passive sensing matrices but as *computational sensing substrates* capable of simultaneously detecting environmental inputs and performing distributed information processing. While traditional biosensors typically generate raw signals requiring external post-processing, computational biosensors integrate biological recognition and logical operations within the sensing layer itself, producing intrinsically processed, near-decision-ready outputs. In the fungal context, electrophysiological signaling in mycelium provides a natural mechanism for such integration, suggesting a pathway toward biosensors that compute and sense concurrently, thereby reducing system complexity while enhancing autonomy and portability.

A summary of the principal findings and functional capabilities reported across fungal computing systems is provided in Table 11.

Overall, the field is progressing from theoretical demonstrations of computational universality toward functional and bio-integrated electronic components. Nevertheless, key challenges remain, including intrinsic biological variability, sensitivity to environmental fluctuations, and limitations in device stability and miniaturization. Addressing these constraints will be essential to enable the broader adoption of fungal-based unconventional computing as a reliable platform for intelligent biosensing and neuromorphic hardware.

### 5.3. Functional Fungal Living Materials for Wearables, Fungal Skin, and Smart Buildings

**Figure 14:**
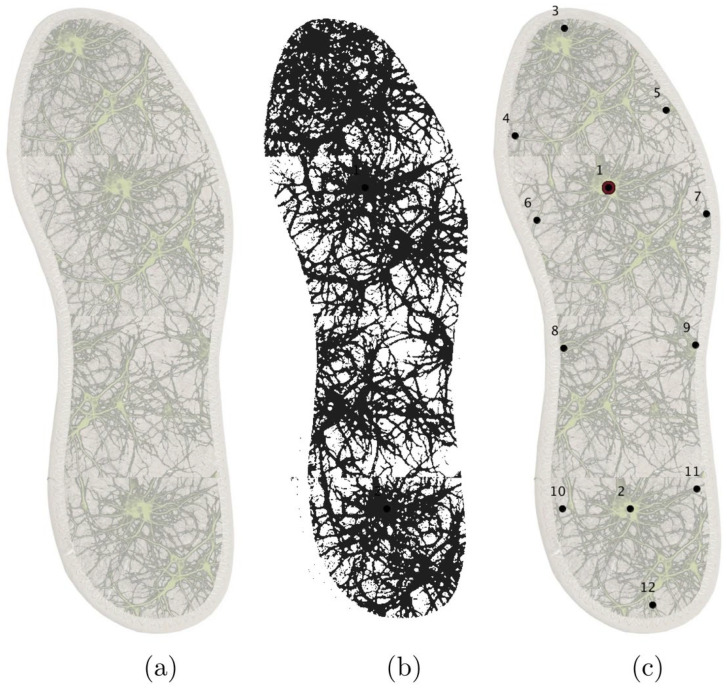
Conceptual framework for responsive fungal insoles for pressure-sensitive biosensing based on living mycelium networks. (**a**) Original image of the fungal colony, showing the spatial organization of the mycelial network. (**b**) Binarized conductive matrix derived from the mycelium image, representing the effective connectivity of the living network and enabling computational modeling of excitation dynamics. (**c**) Spatial configuration of distributed electrodes used to probe signal propagation across the mycelium structure. Reproduced from Nikolaidou et al. [222] under the Creative Commons Attribution License (CC BY 4.0).

Mycelium—long regarded as the hidden structural framework of fungi—has recently emerged as a biologically sophisticated and functional scaffold for engineering living materials with intrinsic sensing capabilities. Owing to their hierarchical porosity, metabolic responsiveness, and endogenous electrical signalling, filamentous fungi enable the development of fungal living materials (FLMs) that function as integrated biosensing interfaces rather than passive structural supports. These systems support stimulus detection, signal transduction, and adaptive response within a single biological material platform. Early experimental evidence for mycelium-based biosensing was provided by Adamatzky et al., who demonstrated that hemp-based textiles colonized by *Pleurotus ostreatus* generate distinct electrical signatures in response to physical, chemical, and mechanical stimuli [220]. Nutrient-rich attractants increased spike frequency and amplitude, ethanol exposure induced rapid localized depolarization, and mechanical stretching produced action-potential-like electrical impulses. These results established mycelium as a responsive sensing layer capable of transducing external stimuli into measurable electrical signals. However, sensor performance was strongly dependent on environmental conditions, with sustained humidity required to prevent desiccation and considerable variability observed in signal propagation and temporal dynamics [221]. Subsequent studies confirmed that mechanically responsive FLMs can be engineered on alternative substrates. Mycelium-infused capillary matting used as pressure-sensitive insoles (Figure 14) exhibited reproducible electrical responses to compressive loads, with spike patterns correlating with both pressure magnitude and spatial distribution, thereby demonstrating the feasibility of FLMs as distributed, body-interfaced sensing systems [222].

Numerical modelling further showed that excitation waves propagated through the mycelial network, enabling discrimination among pressure locations. Despite these capabilities, response times remained slower than those of conventional electronic pressure sensors, and signal sensitivity decreased as hydration levels declined. Fungal living materials have also demonstrated sensitivity to biochemical stimuli. Exposure of *P. ostreatus* mycelium mats to hydrocortisone produced detectable alterations in electrical spiking activity, reflecting metabolic responses to the hormone. These observations indicate that FLMs can function as biologically responsive sensing platforms for chemical and physiological cues. Nevertheless, signal reproducibility was constrained by moisture content, biological ageing, and inter-channel variability, highlighting the need for improved environmental control and interface stabilization [223]. Beyond fully living systems, chemically modified mycelium has been developed as a stable substrate for bioelectronic sensing platforms. In particular, *Ganoderma lucidum*-derived mycelium skins were processed into mechanically robust, thermally stable, and biodegradable foils capable of supporting conductive metal films, laser-patterned circuits, strain sensors, and flexible near-field communication (NFC) devices [224]. These systems preserve key advantages of fungal materials—such as sustainability and structural tunability—while offering improved reproducibility and integration with conventional sensor architectures. A comparative summary of functional performance, shared advantages, and recurring constraints is provided in Table 12.

Overall, these studies demonstrate that fungal living materials can function as biologically active sensing interfaces in which stimulus recognition, signal transduction, and material-level adaptation are intrinsically coupled. While FLMs offer unique advantages, including biodegradability, self-healing capacity, and multimodal responsiveness, their practical deployment as biosensors remains constrained by environmental sensitivity, biological heterogeneity, slow electrical dynamics, and challenges in long-term stability [221,222,223,224]. Addressing these limitations will be essential for translating mycelium-based living materials into reliable, miniaturized, and application-ready biosensing platforms.

**Figure 15:**
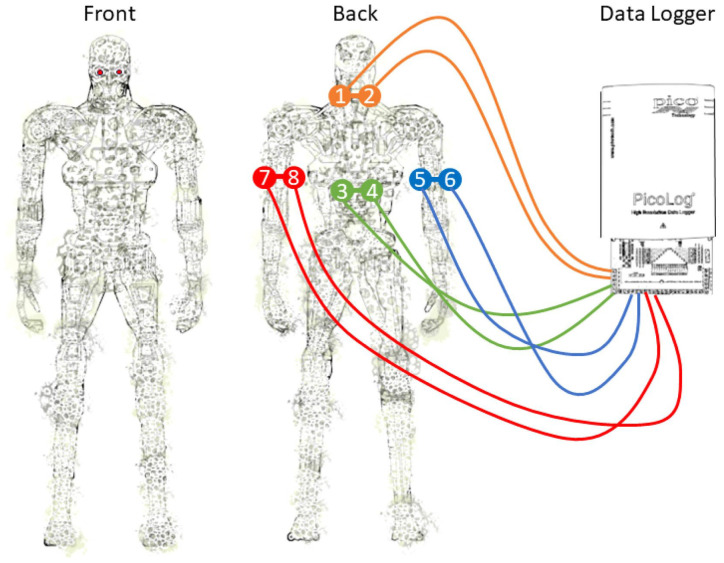
Front and back views of a humanoid model covered with a living mycelium exoskin, showing the schematic placement of electrode pairs and their connection to the data acquisition system. Eight electrodes were inserted on the posterior side of the mycelium-covered model and arranged into four recording channels: neck (channels 1–2), back (channels 3–4), right arm (channels 5–6), and left arm (channels 7–8). The electrode pairs were spaced approximately 1 cm apart. Electrical signals were recorded using a high-resolution PicoLog ADC-24 data logger (Pico Technology, UK). Reproduced from Gandia et al. [227] under the Creative Commons Attribution License (CC BY 4.0).

Fungal skin technologies represent a distinctive class of biohybrid sensing interfaces, in which thin, self-supporting mycelial mats directly transduce external physical and environmental stimuli into measurable electrical signals. Operating at the material level, fungal skins integrate biological signal generation and structural support within a single living system, while relying on external readout architectures for signal acquisition and interpretation. This interface-centered sensing modality positions fungal skins as intrinsically responsive materials rather than conventional, fully integrated biosensor devices. Mechanical, optical, and environmental perturbations are detected through endogenous electrophysiological responses generated by the mycelial network itself and captured via simple electrode readouts, positioning fungal skins as distributed, label-free sensing materials rather than passive substrates. In this respect, fungal skins complement conventional biosensing architectures by providing intrinsic stimulus transduction at the material level, while signal interpretation and system integration remain externally defined. Early experimental studies using skins derived from *Ganoderma resinaceum* and *Ganoderma lucidum* demonstrated that distinct classes of stimuli produce reproducible and stimulus-specific electrical signatures. Mechanical loading and unloading induced high-amplitude transient spikes, whereas optical stimulation resulted in slower, sustained shifts in baseline potential. These differentiated signal patterns indicate that fungal skins can encode stimulus -specific identity through variations in spike amplitude, duration, and temporal structure, fulfilling key requirements for biological signal transduction in sensing applications [225,226]. However, these systems also exhibited notable limitations, including spatial variability between electrode sites, relatively slow response kinetics compared with conventional electronic sensors, and a strict dependence on high humidity to maintain tissue viability and signal stability. Moving beyond planar sensing layers, *Ganoderma sessile* mycelium has been integrated as a reactive biohybrid exoskin on robotic platforms, providing further insight into the sensing behaviour of fungal skins under dynamic conditions. In this configuration (Figure 15), tactile stimuli generated discrete electrical spikes, while illumination induced prolonged potential drifts, confirming that fungal skins retain stimulus discrimination when interfaced with moving structures. The mycelium also exhibited biological self-regeneration and sustained electrophysiological activity over time, highlighting potential advantages for long-term sensing applications [227].

Nevertheless, continuous hydration requirements, susceptibility to desiccation, and uncertainty in mechanical reliability during motion currently constrain practical deployment. A defining functional advantage of fungal skins within sensing architectures is their intrinsic self-healing capability, which directly supports durability and functional recovery following damage. Using *Ganoderma lucidum* pellicles, Elsacker et al. demonstrated that dormant chlamydospores embedded within dried and plasticized mycelium films can regenerate viable tissue after mechanical disruption. This regenerative process restored both structural continuity and surface properties over hours to days, suggesting a unique pathway toward self-repairing sensing materials. However, regeneration efficiency depended strongly on environmental conditions, fungal strain, and thermal history, and uncontrolled regrowth rather than localized repair remains a challenge for precision-engineered systems [228]. Fungal skins have also been explored as biodegradable and flexible substrates for transient biosensing and bioelectronic interfaces. Mycelium-based films support the deposition of conductive inks and simple circuit architectures required for signal readout, while offering mechanical compliance and environmental degradability. These attributes make fungal skins attractive alternatives to petroleum-based polymer substrates in short-lived or environmentally responsive sensing devices. At the same time, maintaining stable signal transmission under mechanical deformation, humidity fluctuations, and repeated handling remains challenging, particularly under conditions that promote renewed mycelial activity [229]. Across reported studies, fungal skins consistently exhibit several sensing-relevant advantages, including intrinsic stimulus responsiveness, label-free signal generation, biodegradability, and self-healing potential. Conversely, common limitations include slow electrical dynamics, dependence on high humidity, biological heterogeneity leading to signal variability, and reduced stability under prolonged mechanical stress. Together, these factors define both the promise and the current technological boundaries of fungal skin-based biohybrid sensing systems. As an emerging and still limited body of work, this area illustrates non-conventional, materials-centered sensing paradigms rather than mature biosensor technologies. A comparative overview of key studies and functional characteristics is provided in Table 13.

Building on earlier work on stimulus-responsive mycelial materials, mycelium-based blocks and composites are increasingly explored as structural substrates that embed sensing-relevant functions, particularly for monitoring mechanical load and moisture in built environments. In this context, sensing functionality is intrinsically material-integrated: environmental or mechanical perturbations modulate the electrophysiological and impedance signatures of colonized composites, which can be captured through simple electrode readouts and interpreted as indicators of internal state changes. This dual functionality—structural support combined with intrinsic signal generation—positions mycelial materials as potential enabling elements for smart building and structural health monitoring concepts, in which construction components simultaneously contribute to load bearing, environmental regulation, and condition monitoring. Adamatzky et al. investigated blocks colonized by *Ganoderma resinaceum* and *Pleurotus djamor* and reported stimulus-specific electrical dynamics associated with mechanical loading, repeated stimulation, and moisture variation. Heavier loads elicited higher-amplitude electrical spiking patterns, while repeated stimulation produced habituation-like changes in activity. Importantly, electrical signalling declined sharply in dried or non-viable tissues, highlighting hydration and physiological state as dominant variables governing response consistency and long-term operability [230]. Complementary work on *Pleurotus ostreatus* composites similarly showed strong moisture-dependent electrical activity, including spike trains during drying and rehydration cycles. Impedance measurements further revealed low-pass filtering behaviour that varied with water content and sample geometry, supporting the view that mycelial composites can provide electrical observables relevant to environmental condition tracking and materials monitoring [231]. Key studies [230,231,232] on building-scale mycelium materials and their functional outcomes are summarized in Table 14.

**Figure 16:**
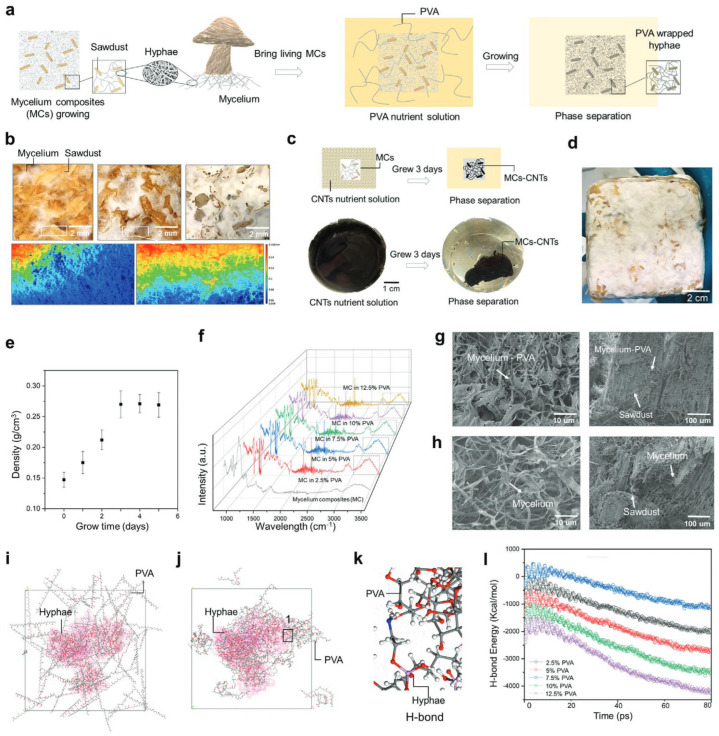
Biofabrication of self-regenerative mycelium-based composites enabled by living growth and phase separation. (**a**) Schematic illustration of growth-driven fabrication: living mycelium composites (MCs) are introduced into a PVA-containing nutrient medium, where growing hyphae induce phase separation and assemble PVA onto the mycelial network to form mycelium–PVA composites (MPCs). (**b**) Macroscopic morphologies of MCs during growth (4, 7, and 12 days) with corresponding 3D optical microscopic images; colors indicate height distribution. (**c**) Phase separation of carbon nanotubes (CNTs) induced by living MCs after 3 days of growth, leading to CNT adsorption onto the mycelium and clarification of the solution. (**d**) Digital photograph of the fabricated MPC. (**e**) Density evolution of MPCs as a function of growth time (g cm^−3^). (**f**) Fourier-transform infrared (FTIR) spectra of MCs cultivated in media with different PVA mass fractions (wavenumber in cm^−1^). (**g**) SEM images of MPC cross-sections showing PVA-wrapped hyphae and the sawdust–mycelium interface. (**h**) SEM images of MC cross-sections for comparison. (**i**) Initial molecular dynamics (MD) model of MCs with 10% PVA. (**j**) Stable MD configuration after dynamic optimization showing PVA adsorption onto hyphae. (**k**) Enlarged view highlighting hydrogen-bond formation between hyphae and PVA. (**l**) Evolution of hydrogen-bond energy during MD simulation. Reproduced from Wang et al. [232] under the Creative Commons Attribution License (CC BY 4.0).

Materials-engineering approaches have begun to address durability and functional integration at larger scales. Entangled composites combining *Ganoderma lucidum* with poly(vinyl alcohol) (PVA) yielded lightweight structures with improved mechanical robustness and regrowth-enabled repair after small cuts, while maintaining compressive performance and low water uptake [232] (Figure 16).

These results indicate that self-regenerative functionality can be coupled with engineering-relevant material properties, although reproducible colonization, network uniformity, and performance stability under fluctuating environmental conditions remain central challenges. Across building-scale implementations, signal variability arising from biological heterogeneity and electrode placement, strong dependence on hydration, and slower response dynamics compared with conventional electronic sensors remain key bottlenecks for translation. A prominent large-scale vision for mycelium-based materials was articulated by the NASA NIAC Myco-architecture project, which framed mycelium composites as engineered living materials for autonomous habitat construction and sustainability-driven building concepts. The approach proposed lightweight payloads consisting of spores and nutrient substrates that could be activated with locally available water to form insulating structures with minimal energy input, while also considering system-level integration of functional layers and embedded monitoring components. Although small-scale prototypes and growth-control strategies were demonstrated, consistent large-scale fabrication, reproducible mechanical properties, and robust integration of embedded functional systems remain unresolved. These findings support the view that mycelium may be most effective as a functional and responsive component within composite assemblies rather than as a sole load-bearing material [233]. In a related effort focused on in-space bio-manufacturing feasibility, a standardized cultivation and fabrication workflow was developed and terrestrially tested for producing modular mycelium-derived tiles within strict mass and volume constraints, representing a practical step toward repeatable biofabrication architectures suitable for spaceflight contexts. However, long-term durability and stability under microgravity, radiation, and other space-relevant stressors still require validation prior to operational deployment [234]. Collectively, these studies indicate that mycelium-based building materials can couple structural roles with electrical and impedance readouts that reflect load and moisture state. Achieving reliable implementation will depend on controlling hydration, reducing biological and geometric variability, and standardizing interfaces for signal acquisition, normalization, and interpretation—requirements that define the current transition from exploratory demonstrations toward functional smart building materials.

## 6. Advancing Fungal Ecology Through Integrated Sensing: Monitoring Fungi and the Environments They Inhabit

As illustrated in the preceding sections, fungi are increasingly recognized not only as platforms for engineered (bio)sensors, but also as key biological actors whose ecological dynamics demand dedicated monitoring strategies. Fungal communities underpin critical ecosystem functions—including nutrient cycling, decomposition, soil aggregation, and plant symbioses—yet their spatial distribution, temporal dynamics, and responses to environmental stress remain incompletely characterized. Large-scale analyses have revealed that fungal conservation is markedly underrepresented in current biodiversity frameworks: most belowground diversity hotspots fall outside protected areas, and global conservation targets continue to rely almost exclusively on aboveground indicators. Because fungal communities are highly sensitive to microclimatic variability and disturbance, continuous and in situ monitoring is essential for assessing both decline and recovery, but remains largely absent from existing conservation strategies [235]. From a sensing perspective, fungal ecology is intrinsically linked to fine-scale gradients in soil moisture, temperature, chemistry, aeration, and resource availability. Environmental sensor networks—originally developed for forest and soil monitoring—therefore provide a critical methodological foundation for studying fungal systems. Distributed IoT architectures capable of continuously measuring temperature, humidity, soil moisture, gas fluxes, and light have demonstrated how abiotic dynamics can be coupled to biological responses in real time, enabling mechanistic interpretation of ecosystem processes [236]. Extending these approaches to fungal ecology offers a pathway to link environmental variability with fungal growth, stress responses, and symbiotic activity. Beyond indirect environmental sensing, fungal bioelectrical activity has emerged as a potential endogenous signal reflecting physiological state and environmental interaction. Electrical signaling in fungi is increasingly discussed not only in technological contexts but also as a plausible mechanism of information exchange within mycelial networks and mycorrhizal systems. However, evidence for functional long-distance signaling in natural ecosystems remains equivocal, in part due to substantial methodological challenges. Field-based electrophysiological recordings are inherently susceptible to electrochemical artefacts—including Donnan potentials at tissue–electrolyte interfaces, polarization effects at metal electrodes, and spurious voltage fluctuations induced by rainfall, ion fluxes in soil water, and mechanical perturbations. Environmental noise from temperature variation, light exposure, and animal activity further complicates signal attribution, making it difficult to unambiguously separate biologically generated electrical activity from abiotic confounders. These limitations are exemplified by recent in situ studies. Fukasawa et al. performed field recordings from *Laccaria bicolor*, *Hebeloma danicum*, and *H. cylindrosporum* sporocarps using subdermal needle electrodes, correlating electrical activity with rainfall, temperature, and localized chemical stimulation. Pronounced voltage fluctuations were consistently observed following precipitation events, occasionally exceeding 100 mV, and directional information flow was inferred between nearby fruit bodies. However, the authors explicitly noted that rain-induced surface charges, ionic throughfall, and soil-mediated electrochemical effects could not be excluded, and recommended controlled follow-up experiments—including artificial rainfall, trenching, and increased replication—to strengthen causal interpretation [237,238]. These studies underscore both the ecological relevance of fungal electrical activity and the difficulty of disentangling endogenous signaling from environmental artefacts under natural conditions. To address these challenges, complementary laboratory-based platforms have been developed to isolate fungal bioelectrical dynamics under controlled conditions while preserving network-level behavior. A notable example is the “mycelium bridge” configuration, in which *Pleurotus ostreatus* forms a self-grown conductive connection between two electrically isolated nodes separated by an air gap. By exploiting the colonizing behavior of hyphae, this setup creates a non-invasive and scalable electrical interface that minimizes direct electrode–tissue perturbation. Impedance spectroscopy and open-circuit potential measurements revealed stable, low-impedance connections (~80–100 kΩ) and reproducible cyclic fluctuations in both impedance and potential with periods of approximately 28–30 h, consistent with intrinsic physiological rhythms likely linked to ion-channel dynamics. Signal transmission efficiency was highest during active growth phases and declined as the mycelium aged or dehydrated, defining an operational lifetime of roughly two weeks [239]. Compared with field-based electrode recordings, the mycelium bridge approach offers improved isolation from soil-mediated artefacts, reduced sensitivity to external electrochemical noise, and clearer attribution of measured signals to fungal physiological processes. At the same time, it remains a laboratory construct that simplifies environmental complexity and does not fully capture the heterogeneity of natural soil systems. Together, these approaches highlight a fundamental trade-off in fungal electrophysiology: field measurements maximize ecological realism but suffer from confounding variables, whereas controlled platforms enhance signal interpretability at the cost of environmental fidelity. When interpreted from a sensing-systems perspective, fungal electrical activity—whether recorded in situ or under controlled conditions—should therefore be viewed not as a direct analogue of neuronal action potentials, but as an integrated physiological readout shaped by hydration, ionic gradients, growth state, and environmental coupling. Importantly, the relevance of these signals extends beyond unconventional computing paradigms. Electrical and impedance signatures in mycelial networks can serve as proxies for moisture availability, mechanical perturbation, nutrient flux, and physiological stress, positioning fungi as intrinsic components of hybrid ecological sensing systems for soil monitoring, precision agriculture, and environmental assessment.

**Figure 17:**
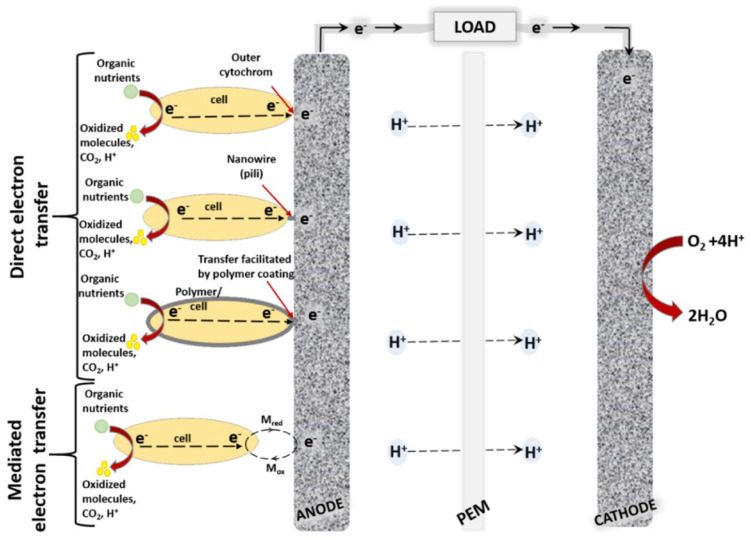
General Scheme of a Microbial Biofuel Cell (MBFC). Schematic illustrating the operation of an MBFC, highlighting the two primary mechanisms of electron transfer from the microbe to the anode: Direct Electron Transfer (DET) and Mediated Electron Transfer (MET). e^−^ denotes electrons; H^+^ denotes protons; Mred and Mox represent the reduced and oxidized forms of the redox mediator, respectively; PEM indicates the proton exchange membrane. Reproduced from Ref. [240].

Taken together, current evidence indicates that advancing fungal ecological sensing will require methodological convergence: combining environmental sensor networks, carefully designed electrophysiological interfaces, and controlled bioelectrical platforms to separate biological signal from artefact. This need directly parallels the constraints identified in Section 5.1 for engineered biosensors, where electrode configuration, signal-to-noise limitations, and interface design were shown to be central challenges. Addressing these shared limitations will be essential for translating fungal bioelectrical phenomena into robust tools for ecological monitoring and data-driven environmental management. Building on this perspective, microbial fuel cells (MFCs) [240,241,242] provide a complementary and more application-oriented framework in which fungal metabolism is directly coupled to electrochemical transduction and quantifiable electrical output (Figure 17).

**Figure 18:**
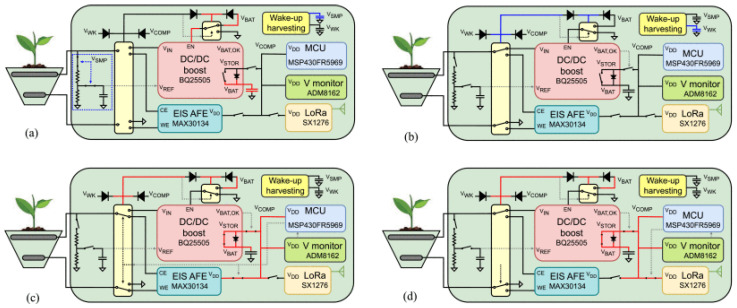
Sensor node block diagram and operational phases of the PMFC-powered EIS system. (**a**) Initial wake-up phase: the wake-up harvesting circuitry powers the OCV sampling block before enabling the input power and sensing path (IPSP). (**b**) Energy harvesting phase: the DC/DC boost converter charges the storage capacitor while the MCU remains in low-power mode. (**c**) EIS measurement phase: upon reaching the threshold voltage, the MCU activates the EIS analog front-end (AFE) and connects the PMFC electrodes to perform impedance measurements. (**d**) Data transmission phase: after EIS completion and further charging, the LoRa transceiver is powered to transmit the acquired data before the system returns to rest mode. Colored areas and arrows indicate the active functional blocks and power-flow paths during each operational phase. Reproduced from Doglioni et al. [243] under the Creative Commons Attribution License (CC BY 4.0).

In contrast to passive electrophysiological recordings, microbial fuel cell (MFC)-based systems actively convert biochemical processes occurring within fungal biomass into measurable current or voltage signals. In doing so, they couple biological activity and signal generation within a single functional architecture. Owing to this integration, MFCs have attracted increasing attention as self-powered platforms for real-time, in situ environmental monitoring. Their portability, low operational cost, and capacity for autonomous operation make them particularly attractive for deployment in remote or spatially distributed sensing contexts. MFC-based systems have been applied to the detection of key water-quality indicators, including biochemical oxygen demand (BOD), chemical oxygen demand (COD), and general toxicity, where changes in microbial metabolic activity are directly reflected in variations in electrical output [240,241]. Within this broader class, fungal fuel cells (FFCs) represent a specialized configuration in which fungal metabolism is directly incorporated into the electrochemical system. Proof-of-concept studies have demonstrated that FFCs can simultaneously generate electrical power and produce electrical signatures sensitive to water quality and toxic stress, highlighting their potential as self-sustaining bioelectrochemical sensing platforms [240,241]. Despite these advantages, several technical limitations currently constrain broader application. Electron transfer between fungal biomass and electrode surfaces is often inefficient, resulting in low power densities, and electrical output may decrease markedly in the presence of toxic compounds. In addition, reliance on expensive electrode materials or complex nanostructured interfaces can hinder scalability and real-world implementation. Addressing these challenges will require simplified reactor designs, the use of more affordable electrode materials, and improved strategies for signal interpretation. In this context, mathematical modelling and data-driven approaches are expected to play an increasingly important role in enhancing sensitivity, robustness, and operational stability [240,241,242]. Related developments in plant microbial fuel cells (PMFCs) further illustrate both the opportunities and unresolved questions associated with fuel-cell-based monitoring systems. Although PMFCs have been employed to track plant-related parameters using electrochemical impedance spectroscopy (EIS), the precise contribution of plant physiological processes to the recorded electrochemical signals remains incompletely understood. Clarifying this relationship is essential for the development of reliable, low-cost tools for plant and ecosystem monitoring at larger spatial scales [243] (Figure 18).

By extension, similar considerations apply to fungal systems. If electrical outputs from fungal fuel cells can be more clearly linked to specific physiological states and environmental drivers, fuel-cell-based architectures may offer a route toward autonomous platforms capable of reporting on fungal activity within soils and aquatic environments.

While electrophysiological recordings and bioelectrochemical systems capture fungal activity at the scale of individual networks or experimental platforms, complementary insights can be obtained by extending observation to the mesoscale and landscape level, where fungal presence and dynamics are inferred indirectly through their impact the physical properties of the subsurface. Mesoscale and landscape-scale geophysical techniques, which measure electrical properties such as resistivity and conductivity, provide a valuable complement to laboratory and bioelectrochemical studies by enabling non-invasive investigation of fungal networks in situ. Electrical geophysical methods—including shallow-depth conductivity meters (CMD-Tiny) and high-frequency ground-penetrating radar (1200 MHz GPR)—have been applied across multiple field sites to identify mycelial networks based on their conductivity contrasts and moisture-retention characteristics. This approach offers a sustainable alternative to destructive sampling and allows repeated monitoring of spatial and temporal changes in belowground fungal systems [244]. Together, these mesoscale observations complement microscale electrical measurements and contribute to a multiscale perspective on fungal network organization and function. Further insight into environmentally responsive fungal dynamics is provided by non-invasive imaging platforms that capture fungal–plant interactions as spatially and temporally resolved readouts of subsurface conditions. Minirhizotron systems enable real-time, high-resolution monitoring of belowground fungal networks and, when combined with automated image acquisition, generate longitudinal datasets that reflect fungal responses to variations in temperature, moisture, and soil structure. Although these platforms are subject to technical limitations—including reduced visibility of fine hyphae, installation-related artifacts, and restricted sampling footprints—they provide a unique, non-destructive window into living fungal systems.

Long-term minirhizotron observations in a warmed peatland ecosystem, for example, revealed that fungal abundance and morphological traits varied systematically with environmental parameters, including prolonged periods of mycorrhizal activity under elevated temperatures [245]. Such findings indicate that fungal network structure and temporal dynamics encode information about surrounding environmental conditions, provided that these variations are captured with sufficient spatial and temporal resolution. Such imaging-derived signals can be interpreted as distributed, in situ sensing readouts of environmental state, rather than purely descriptive ecological observations. The increasing complexity and volume of imaging data have driven the adoption of artificial intelligence and machine-learning approaches to extract quantitative information from heterogeneous soil environments. Deep-learning-based segmentation and classification methods now enable automated identification of fungal structures, reducing reliance on manual scoring and improving reproducibility across datasets. In plant–fungal systems, these tools support high-throughput analysis by transforming imaging outputs into quantitative descriptors linked to functional state rather than purely morphological features [246]. Between 2021 and 2024, several studies demonstrated that machine-learning frameworks can convert mycorrhizal imaging data into standardized and predictive metrics of fungal activity. Convolutional neural networks, region-based segmentation models, and multiple-instance learning approaches enabled structure-specific quantification of arbuscules, hyphae, and vesicles, improving sensitivity and biological interpretability relative to traditional manual assessments [247,248,249]. When coupled with statistical or predictive modelling, these features supported estimation of colonization status and functional responses, shifting analysis from descriptive observation toward inference-driven interpretation. Despite these advances, current workflows remain constrained by the need for expert-annotated training datasets and tightly controlled sample preparation, including consistent staining protocols and illumination conditions. These requirements continue to limit full automation, underscoring that progress in this area will depend not only on algorithmic development but also on standardized acquisition strategies that coherently link biological observation with computational analysis.

**Figure 19:**
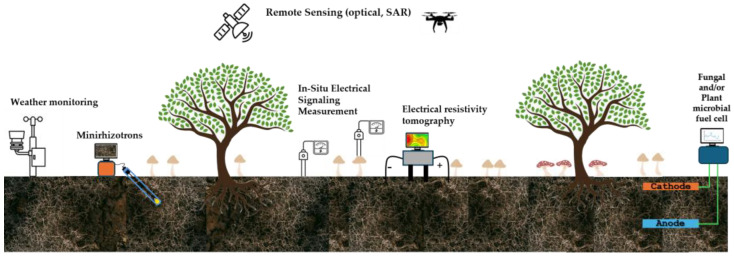
Schematic representation of an integrated, multiscale and multimodal sensing framework at the plant–soil–fungus interface in forest ecosystems, combining remote sensing, meteorological stations, soil probes, and in-habitat fungal biosensors.

Therefore, the future of fungal ecology research—and its translation into sensing, modelling, and biohybrid technological applications—increasingly depends on technological innovation capable of resolving belowground processes across multiple spatial and temporal scales. Advancing this field requires the integration of computational modelling, data-driven analytics, remote-sensing approaches, and in situ measurement systems that together can capture the dynamics of fungal communities within heterogeneous soil environments. Mycorrhizal fungi, in particular, remain challenging to investigate directly due to their cryptic lifestyles, extensive spatial distributions, and tightly coupled interactions with host plants. In this context, remote sensing has become an essential, non-invasive tool for characterizing soil and vegetation properties that both influence and reflect fungal activity. Techniques such as spectral reflectance analysis, thermal infrared imaging, and synthetic aperture radar (SAR) enable landscape-scale assessment of soil moisture, texture, organic matter content, and vegetation status. Because mycelial growth and function both respond to and modify these parameters, remote-sensing observations provide indirect yet ecologically informative insights into the bidirectional coupling between fungi and their environments. When combined with in situ measurements and AI-based analytical frameworks, these approaches enhance the interpretation of soil heterogeneity and temporal variability beyond what localized sampling alone can achieve. Remote-sensing indices further support this integration. The Normalized Difference Vegetation Index (NDVI), widely used as a proxy for vegetation greenness and photosynthetic activity, can indirectly reflect soil biological processes, including fungal dynamics. In a recent study, Sørensen et al. integrated NDVI with machine-learning models to link crop health with fungal microbiome composition, revealing positive associations between higher NDVI values and beneficial fungal genera (e.g., *Tomentella*, *Mortierella*), and correlations between reduced NDVI and pathogenic taxa such as *Fusarium*. These findings demonstrate how remotely sensed vegetation signals can be leveraged to infer underlying fungal community structure and ecological function [250]. Taken together, the current body of research highlights the need for a genuinely multiscale and integrative framework for fungal ecology—one that links molecular and physiological processes to ecosystem-level patterns (Figure 19).

Importantly, this convergence between ecological research and sensing technologies should not be viewed solely as a methodological enhancement. Rather, it reflects a broader conceptual shift in which fungi are recognized not only as components of ecosystems, but also as active, responsive elements embedded within them. From this perspective, fungal mycelia emerge as particularly promising candidates for in situ biosensing. Their pervasive distribution in soil, sensitivity to physicochemical fluctuations, and capacity for bioelectrical signaling position mycelial networks as natural interfaces between environmental change and measurable biological responses. If fungal networks can already function as responsive elements within engineered materials—where living structures contribute to sensing, regulation, and adaptive behavior—then extending this paradigm to natural ecosystems becomes a logical progression. By integrating intrinsic fungal responses with conventional sensor outputs, remote-sensing data, and controlled laboratory measurements, mycelial networks could operate as autonomous, distributed sensing interfaces that couple biological processes directly to technological monitoring systems. Such an approach moves beyond passive observation and toward biologically grounded sensing architectures capable of continuous, spatially distributed ecosystem monitoring. In this way, the fusion of sensing technologies with fungal ecology not only advances fundamental understanding but also lays the foundation for living sensor networks in which fungal mycelium functions as an in-habitat biosensor, enabling early detection of environmental stress, improved soil monitoring, and next-generation strategies for ecosystem assessment [251].

## 7. Fungi and AI: Reciprocal Insights for Ecological, Computational and Sensing Applications

**Figure 20:**
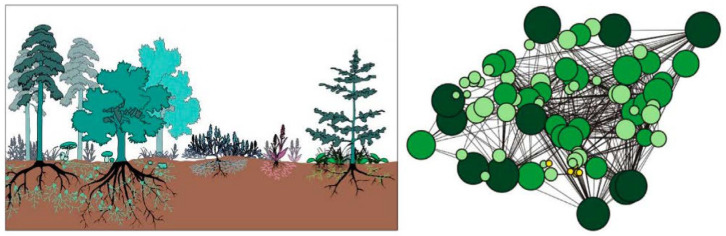
Conceptual representation of a mycorrhizal network (MN) linking plant species of different sizes and functional roles, together with its corresponding topological map. In the left panel, tree size and color schematically reflect differences in plant age and ecological role. In the network visualization (right), nodes represent individual trees and are scaled and colored according to age and ecological role (dark green: mature “mother” trees; light green: younger individuals; yellow: seedlings or small trees). Edges represent Euclidean-distance-based connectivity, with line thickness indicating the degree of connection as determined by the number of distinct ectomycorrhizal genets shared between plant individuals. Reproduced from Ref. [252].

Fungal mycelium operates as a vast, decentralized system that balances survival with efficient resource distribution, representing one of nature’s most effective examples of distributed intelligence. Its functioning relies on local decision-making: each hyphal tip behaves as an autonomous sensing unit that explores the surrounding substrate. When encountering nutrient-rich patches or plant roots, the tip detects local chemical gradients and makes a simple decision—grow, branch, or cease extension. The accumulation of these local actions collectively produces the emergent, complex architecture of the mycelial network. Resource allocation within the network is similarly dynamic. Carbon supplied by host plants is preferentially redirected toward roots that contribute proportionally greater water and mineral resources, maximizing the mutualistic benefit. To maintain efficiency, the mycelium reorganizes continuously: hyphae in nutrient-poor zones are degraded or reabsorbed, while growth intensifies in nutrient-rich areas or toward stressed roots, for example during drought. This capacity for rapid structural adjustment confers resilience and adaptability under fluctuating environmental conditions. Mycorrhizal partnerships further amplify these dynamics by linking fungal networks with multiple plants, creating shared resource highways across the soil [252] (Figure 20).

Understanding these decentralized decision-making processes provides a natural blueprint for artificial intelligence and computational modeling, where local rules and interactions can be leveraged to predict network behavior, optimize resource allocation, and design bio-inspired algorithms for distributed systems [253].

Drawing inspiration from the decentralized organization and resource-optimization strategies of fungal networks, mycelial and mycorrhizal systems have informed a novel class of nature-inspired computational approaches. Most notably, metaheuristic algorithms—a family of high-level optimization techniques designed to efficiently address complex problems—mirror the organizational logic of fungal networks. Fungal strategies for spatial exploration, dynamic allocation, and feedback-driven adaptation parallel the iterative search and solution-refinement mechanisms central to metaheuristic optimization. These algorithms are widely used in artificial intelligence and machine learning, supporting tasks such as clustering, feature selection, hyperparameter tuning, and neural network training [254]. Unlike traditional optimization methods that depend on linear approximations or gradient information, metaheuristic algorithms employ adaptive, derivative-free search processes to navigate large and complex solution spaces. They are effective at escaping local optima and identifying globally superior solutions. Their strengths include flexibility, problem-independence, conceptual simplicity, and the use of stochasticity, which enhances exploration in high-dimensional or uncertain environments. Many metaheuristics are also self-adaptive and can be hybridized with complementary techniques, further improving performance [255]. Their robustness makes metaheuristics particularly suitable for environments characterized by noise and uncertainty, supporting reliable performance in real-world applications.

**Figure 21:**
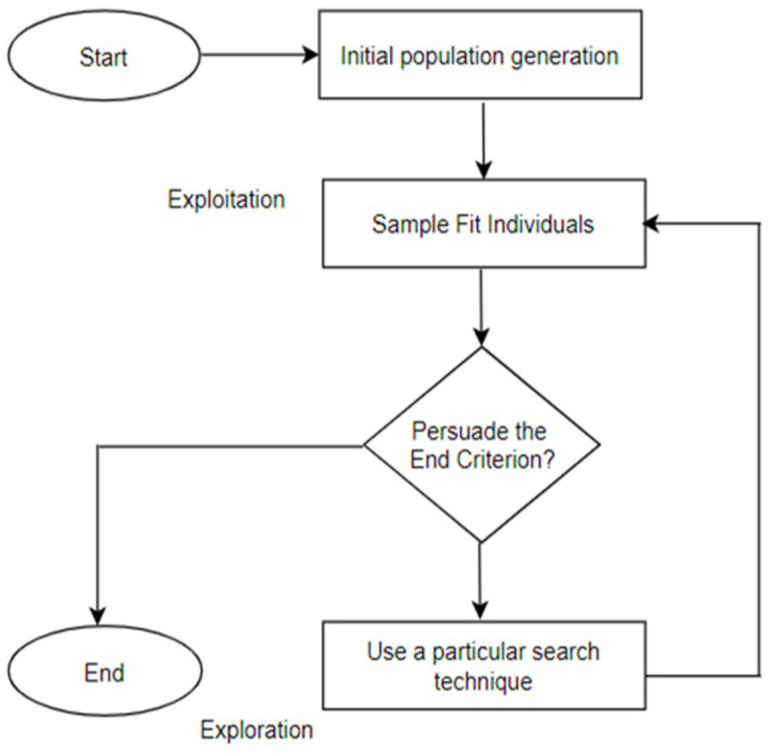
Schematic representation of a generic metaheuristic optimization workflow, illustrating the iterative logic governing the balance and switching between exploration and exploitation phases. The algorithm alternates between sampling high-fitness candidate solutions and applying search strategies to explore new regions of the solution space until a termination criterion is satisfied. Reproduced from Ref. [255].

Historically, many metaheuristic algorithms were designed to emulate natural systems shaped by evolutionary pressures. Their defining characteristics (Figure 21) include:

- Exploration, which allows the algorithm to survey a broad range of potential solutions;
- Exploitation, which focuses the search around promising candidates to refine and improve solution quality.

Fungal-inspired metaheuristic optimization algorithms have emerged as a promising class of nature-inspired computational techniques that model decentralized, adaptive, and resilient behavioural strategies observed across the fungal kingdom. These methods draw on ecological survival mechanisms such as resource exchange in mycorrhizal networks, hyphal foraging, chemotropic growth, and spore-mediated dispersal. By translating these biological principles into computational operators, fungal-inspired metaheuristics provide an effective balance between global exploration and local exploitation, enabling robust performance in complex and uncertain optimization landscapes. A central family within this domain is grounded in the biology of mycorrhizal symbiosis. The Mycorrhiza Optimization Algorithm (MOA) (Figure 13) and its derivatives—namely the Continuous Mycorrhiza Optimization Algorithm (CMOA) and the Discrete Mycorrhiza Optimization Algorithm (DMOA)—simulate cooperative, competitive, and defensive interactions between fungi and host plants within shared mycorrhizal networks. These models incorporate ecological dynamics formulated through Lotka–Volterra equations, enabling adaptive resource allocation in response to changes in the fitness landscape. CMOA, introduced by Valdez et al., extended the original framework into continuous numerical domains, strengthening local exploitation through refined update rules and adaptive transfer coefficients while maintaining stability across iterations. DMOA adapted the same ecological principles to discrete-variable spaces, enabling effective handling of scheduling, combinatorial optimization, and other non-continuous problem types. Together, these complementary algorithms indicate that mycorrhizal-inspired models can be tailored to address both continuous and discrete optimization challenges with notable accuracy and robustness [254,256,257,258,259,260]. A related method, the Mycorrhiza Tree Optimization Algorithm (MTOA), integrated tree growth dynamics with mycorrhizal cooperation rules and was reported to improve performance on benchmark suites by combining ecological operators that promoted both solution diversity and convergence efficiency [261]. Comparative analyses suggested complementary strengths among mycorrhiza-based approaches: CMOA tended to deliver faster convergence and higher precision in continuous search domains due to efficient exploitation mechanisms, whereas DMOA showed greater robustness in discrete spaces by preserving solution diversity and reducing premature stagnation. These findings support the view that hybrid frameworks combining both variants could leverage their respective advantages in complex real-world optimization problems [262]. Beyond mycorrhizal symbiosis, several algorithms have drawn inspiration from fungal growth mechanics and reproductive strategies. The Fungal Growth Optimizer (FGO) modeled hyphal tip extension, branching, and chemotropic directional growth to guide stochastic search processes, and its performance was reported as statistically significant and robust across diverse multimodal benchmark landscapes [261]. The Bioluminescent Fungi Optimization Algorithm (BFOA) exploited spore dispersal mediated by bioluminescent attraction, forming a dual-agent search system that strengthened global exploration and was applied to engineering design problems [262]. The Fungi Kingdom Expansion (FKE) Algorithm abstracted hyphal expansion dynamics via mobile and immobile biomass models, integrating chaotic sine-map exploration with directional, fitness-driven exploitation; it was reported to perform strongly in antenna optimization tasks, including a 100% success rate across multiple scenarios [263]. Collectively, these methods translate fungal strategies—including rapid spore dispersal, chemotropic navigation, and adaptive network expansion—into computational operators that balance search diversity and convergence. Table 15 summarizes the reported algorithms and their key characteristics.

Common strengths across fungal-inspired metaheuristics include adaptability, maintenance of population diversity, resilience against premature convergence, and robust performance in high-dimensional or noisy environments. These advantages reflect core properties of fungal systems—decentralization, redundancy, and distributed resource management—which naturally support a balance between cooperative information sharing and competitive foraging. Many fungal-inspired algorithms also incorporate problem-relevant ecological behaviours—such as nutrient redistribution, hyphal competition, and symbiotic exchange—that enrich the operator set for navigating complex optimization landscapes. Despite these strengths, fungal-inspired algorithms share limitations. Their performance often depends on careful parameter tuning, and the biological realism embedded in their design—while conceptually advantageous—can introduce computational overhead relative to simpler metaheuristics. Complex multi-operator update schemes, particularly those incorporating chaotic dynamics or multi-agent interactions (e.g., FKE and BFOA), may further increase computational cost. As with population-based heuristics generally, these methods do not guarantee reaching the global optimum, and inadequate parameterization can lead to stagnation or inefficient exploration. Moreover, many fungal-inspired algorithms still require broader benchmarking across diverse problem classes and deeper theoretical analysis to clarify stability, scalability, and convergence properties. Even with these challenges, the success of current approaches positions the fungal kingdom as a rich source of inspiration for next-generation optimization algorithms, highlighting principles of distributed, resilient, and adaptive computational intelligence. As research on fungal behaviour and ecological interactions advances, the operator-level mapping between biological mechanisms and algorithmic design is expected to become more precise, expanding opportunities for more rigorous and application-relevant metaheuristic frameworks. In this context, data-driven and artificial intelligence-based methods play a key role in formalizing ecological complexity into computationally tractable representations, enabling both algorithmic abstraction and functional interpretation of fungal responses. The interplay between artificial intelligence—an accelerating technological frontier—and the fungal kingdom, which evolved over more than a billion years yet remains comparatively undercharacterized, is shaping an increasingly integrative research paradigm. This convergence is progressively shifting fungal science from predominantly descriptive observation toward quantitative inference, prediction, and application-oriented workflows. In the context of living and in-habitat sensing systems, such a transition is particularly relevant, as fungal responses emerge from the integration of multiple environmental cues rather than from isolated analyte recognition. Artificial intelligence therefore provides a critical analytical layer for decoding complex, state-dependent fungal behaviour across ecological, biochemical, and sensing-related domains. Machine-learning approaches have been successfully applied to infer fungal lifestyle traits from high-dimensional genomic features, providing early demonstrations of AI-enabled functional classification. For example, K-Nearest Neighbour (KNN) models trained on hundreds of Sordariomycetes genomes identified lifestyle-associated protein signatures—such as secretome components and effectors—allowing discrimination between plant pathogens and endophytes with high accuracy [264]. While originally developed for ecological and phytopathological applications, these approaches illustrate the broader potential of AI to extract latent functional states from complex fungal datasets, a capability directly relevant to biological sensing systems based on state inference rather than single-target detection. As discussed in Section 6, deep learning and machine-learning tools have also improved the segmentation, classification, and prediction of mycorrhizal colonization patterns, addressing key limitations of manual scoring. By enabling automated quantification of both fungal structures and host root traits, these methods support higher-throughput and more reproducible assessment of fungal–plant interactions and facilitate a transition from descriptive imaging toward inference-driven interpretation of fungal functional state [246,247,248,249]. Such developments are particularly relevant for living sensing architectures, where signal interpretation depends on understanding how biological structure, activity, and environment jointly shape measurable outputs. Beyond laboratory-controlled settings, AI-driven workflows have been employed to analyse fungal behaviour under heterogeneous and dynamic environmental conditions. Studies on edible species such as *Boletus edulis* and *B. reticulatus* demonstrated that machine-learning models can identify the environmental variables most strongly influencing fungal growth across chestnut orchards of different ages, providing predictive insight into mycelial behaviour and ecological responses [265]. Similarly, AI-based analyses applied to *Morchella importuna* revealed how soil chemistry and climatic parameters shape ascocarp development under field conditions, highlighting both the potential and the current limitations of data-driven inference of fungal functional states in real-world environments [266]. Although constrained by sample size, geographic scope, and environmental variability, these studies illustrate how AI enables the decoding of distributed fungal responses to complex environmental stimuli—an essential requirement for in-habitat biosensing systems operating outside controlled laboratory conditions. In parallel, AI-driven databases and modelling tools are accelerating fungal bioprospecting by enabling rapid identification and prioritization of bioactive compounds. For genera such as *Ganoderma*, machine-learning approaches have shortened discovery timelines and improved selection accuracy for compounds relevant to functional foods, nutraceuticals, and therapeutics [267]. While not directly biosensor-oriented, these workflows further exemplify AI-assisted interpretation of complex fungal biochemical outputs and reinforce the role of data-driven methods in navigating the functional diversity of fungal systems. Within biosensing contexts, artificial intelligence plays an increasingly direct role in both the characterization of fungal-derived biorecognition elements and the interpretation of the complex signals they generate. As discussed in Section 3.2.2, machine-learning-guided analysis of lectin–glycan interactions enabled elucidation of binding rules for multiple fungal lectins, including *Aleuria aurantia* and *Aspergillus oryzae* lectins. By integrating curated glycan descriptors with computationally derived motifs, these models resolved subtle determinants of specificity and inhibition, improving selectivity and interpretability in lectin-based sensing platforms [173]. Complementary examples include AI-assisted structural modelling of fungal lectins lacking experimentally resolved three-dimensional structures, such as those from *Ganoderma applanatum*, where machine-learning predictions informed electrode immobilization strategies and enhanced analytical performance [174]. Similarly, artificial neural networks (ANNs) have been applied to fungal laccase-based biosensors to capture nonlinear relationships between electrochemical signals and analyte concentration, achieving high predictive accuracy and robust real-time monitoring of phenolic pollutants [268]. Taken together, these studies illustrate a reciprocal and reinforcing relationship between fungal systems and artificial intelligence. The complexity, adaptability, and context-dependent behaviour of fungal networks provide rich, biologically grounded signal sources that challenge conventional sensing paradigms, while AI supplies the analytical and inferential capacity required to decode these signals across ecological, biochemical, and bioelectrical dimensions. This convergence establishes a framework in which ecological understanding, data-driven inference, and biosensing are not separate domains but interdependent components of emerging living and biohybrid fungal-based sensing architectures.

## 8. Safety, Biocompatibility, and Regulatory Considerations

Fungal-based biosensing platforms provide clear advantages in terms of sustainability, functional diversity, and environmental compatibility. Nevertheless, their responsible development and deployment require careful consideration of biosafety, biocompatibility, and regulatory aspects, particularly when fungal enzymes, crude extracts, or living systems are employed. These dimensions should be addressed within a broader safe-by-design and risk-management framework integrating biological safety, device performance, and lifecycle considerations from the earliest development stages.

### 8.1. Biosafety and Allergenicity

Several filamentous fungi commonly investigated for biosensing, including species of *Ganoderma*, *Trametes*, and *Aspergillus*, release airborne spores and proteinaceous components that can act as allergens. Sensitization to fungal allergens has been reported in a relevant fraction of the population and is associated with respiratory hypersensitivity, allergic rhinitis, and asthma, while occupational and outdoor exposure studies further show that sensitization to airborne fungal spores (e.g., *Alternaria*, *Cladosporium*, *Aspergillus*) correlates with increased risk and severity of asthma and rhinitis in exposed populations [269,270]. In fungal-based biosensing, the allergenic risk is highly configuration-dependent. Living cultures and sporulating mycelia represent the highest potential for airborne allergen exposure, whereas non-viable mycelial materials, once fully inactivated and cleaned of loosely bound proteins, tend to show low immunogenicity and good local tissue tolerance, as documented for mycelium-based wound dressings derived from edible species [271]. A related but distinct concern arises from purified fungal enzymes, which behave as high-molecular-weight occupational allergens. Experience from the detergent, baking, and industrial biotechnology sectors indicates that inhalation of airborne enzyme dusts can lead to IgE-mediated sensitization and occupational asthma if exposure is not adequately controlled; however, risk assessments for enzyme-containing products also show that encapsulation, liquid formulations, and appropriate engineering controls are effective in keeping airborne levels below sensitization thresholds [272]. Purified or crude enzyme preparations therefore represent a relevant allergenic hazard primarily during handling as powders or aerosols. Most biosensing configurations rely on immobilized enzymes, inactivated biomass, or encapsulated materials, and under normal use the allergenic risk for end-users is expected to be substantially lower than that associated with direct exposure to airborne spores or uncontained enzyme powders. Nevertheless, potential exposure during fungal cultivation, processing, or device fabrication should not be underestimated. Standard laboratory containment, controlled growth conditions designed to minimize sporulation, and appropriate personal protective equipment represent baseline mitigation measures, complemented by liquid or granulated enzyme formulations, physical encapsulation to prevent dust formation, and local exhaust ventilation [272]. For applications involving prolonged human or environmental exposure, additional design strategies such as hermetic encapsulation of fungal components, the use of non-sensitizing barrier layers compliant with ISO 10993-1 biocompatibility requirements, enzyme purification to remove extraneous protein fractions, and post-processing inactivation of residual viable material are advisable [273].

### 8.2. Crude Extracts and Contamination Risks

As discussed in Section 3.1.3, crude fungal extracts may provide cost-effective and functionally rich sensing elements, but they can also contain undesired components, including mycotoxins and other secondary metabolites. Mycotoxins constitute a broad class of toxic fungal metabolites with well-documented adverse effects on human and animal health, including immunotoxic and carcinogenic outcomes [274,275]. Mitigation strategies include selective purification, toxin screening, strain selection, and cultivation regimes designed to suppress secondary metabolite production. When crude extracts are employed, their use should preferably be limited to contained, non-human-facing applications unless rigorous purification and validation protocols are implemented.

### 8.3. Biocompatibility and Environmental Deployment

For biosensors intended for environmental monitoring or in situ deployment, biocompatibility extends beyond human safety to encompass ecological compatibility. Encapsulation of fungal components, the use of biodegradable and inert matrices, and the avoidance of uncontrolled release of viable fungal material are key design considerations. Non-viable mycelial materials derived from edible species have demonstrated good biocompatibility and low immunogenicity in skin-contact applications, supporting their use as safe structural components in biosensing devices [271]. Living mycelial systems, by contrast, require strict control of growth, containment, and lifecycle management to prevent unintended ecological interactions or environmental persistence.

### 8.4. Regulatory and Translational Considerations

The regulatory landscape for fungal-based biosensing devices remains heterogeneous and strongly application-dependent. Enzyme-based sensors employing purified or immobilized biomolecules generally fit within existing frameworks for biochemical sensing devices, whereas platforms incorporating living fungi or minimally processed extracts are likely to encounter additional regulatory scrutiny, particularly when intended for clinical or diagnostic use [276]. Broader ethical, legal, and regulatory challenges associated with biosensor development—including safety validation, device classification, and responsible deployment—have also been addressed in the context of emerging biosensing technologies. Early consideration of regulatory pathways, standardized reporting of safety-related parameters (e.g., allergenic potential, mycotoxin screening, viability status, and environmental behavior), and alignment with existing biosafety, environmental, and device guidelines (including MDR/IVDR, ISO 14971, ISO 10993-1, and, where relevant, Directive 2001/18/EC) will be crucial for successful translation beyond the laboratory [273,276,277,278,279,280]. Within the European Union, fungal-based biosensors intended for medical or in vitro diagnostic applications fall under Regulation (EU) 2017/745 (MDR) and/or Regulation (EU) 2017/746 (IVDR) [278,279] and are expected to comply with biocompatibility and risk-management requirements as part of an appropriate quality management system, including ISO 10993-1 and ISO 14971 [273,279]. Recent analyses of MDR/IVDR emphasize increased requirements for clinical evidence, more stringent scrutiny of biomaterial-based devices, and the central role of risk management and lifecycle-long post-market surveillance in determining translational success [276].

Taken together, safety, biocompatibility, and regulatory considerations should be regarded not as peripheral constraints but as integral design parameters. Addressing these aspects early in the development process—through safe-by-design strategies, systematic risk management, and proactive regulatory alignment—will support the reliability, societal acceptance, and long-term impact of fungal-based biosensing technologies.

## 9. Conclusions

This review has shown that filamentous fungi can drive transformative advances in (bio)sensing across multiple domains, encompassing secretome-derived biomolecules, mycogenic nanomaterial synthesis, unconventional computation based on mycelial networks, fungal living materials, and emerging ecological monitoring technologies. Together, these research directions highlight fungi as uniquely versatile biological platforms that operate across molecular, material, and ecosystem scales. At the same time, despite substantial progress, each domain faces fundamental scientific and technological challenges that must be addressed before fungal systems can be fully integrated into next-generation sensing technologies. At the biochemical scale, fungal enzymes, binding proteins, and other secretome components have demonstrated exceptional versatility, enabling sensitive detection of phenolics, pesticides, hormones, pathogens, and membrane lipids. However, limitations related to stability, batch-to-batch variability, and cross-reactivity remain significant barriers to widespread deployment. Future progress will require the convergence of multi-omics discovery, AI-assisted protein engineering, fusion-protein design, and advanced immobilization chemistries to achieve robust, selective, and portable biosensing interfaces. Myconanosynthesis has likewise reshaped the biosensing landscape by enabling the sustainable production of diverse nanostructures, including metallic and metal oxide nanoparticles, carbon quantum dots, nanominerals, and nanozymes. These biogenic materials exhibit distinctive catalytic, optical, and electronic properties while offering clear advantages in sustainability and biocompatibility. Nevertheless, their broader application remains constrained by incomplete understanding of biosynthetic mechanisms, limited reproducibility, and challenges in scale-up. Addressing these issues will require systematic mapping of fungal biosynthetic pathways through integrated multi-omics approaches, development of scalable bioreactor platforms, and rigorous benchmarking of fungal nanomaterials against chemically synthesized counterparts in device-level applications. At macroscopic scales, mycelium-based composites and fungal living materials represent one of the most rapidly advancing areas of fungal biosensing research. Mycelium functions simultaneously as a structural and functional scaffold, exhibiting self-healing behavior and stimulus-responsive electrical activity. These properties have enabled early prototypes of wearable sensing systems, adaptive building materials, and biohybrid robotic interfaces. Yet mycelium remains a biologically dynamic and environmentally sensitive material, strongly affected by humidity, desiccation, contamination, and physiological drift. Enhancing long-term stability, reproducibility, and controllable functionality will require innovations such as hybrid protective coatings, standardized growth and post-processing protocols, and improved integration with electronic components. Within this context, fungal electrophysiology has revealed structured electrical impulses propagating through mycelial networks, opening new avenues for unconventional computation and neuromorphic design. Experimental demonstrations of Boolean logic, reservoir computing, and memristive behavior suggest that mycelial networks can function as living substrates for information processing. However, the biological significance of fungal electrical spikes remains incompletely understood, and their slow, environmentally sensitive dynamics pose substantial engineering challenges. Progress in this area will depend on more accurate biophysical models, higher-resolution and less invasive recording technologies, and hybrid bioelectronic architectures capable of stabilizing, amplifying, and interpreting fungal electrical activity. Fungal ecology stands to benefit equally from advances in sensing and monitoring technologies. Many of the emerging tools used to study ecosystems—electrophysiological probes, soil sensors, IoT-based weather stations, minirhizotron imaging, geophysical methods, and remote sensing—are inherently sensoristic. Applied to fungal systems, these approaches have begun to uncover new dimensions of mycorrhizal interactions, fungal communication, and belowground dynamics. Nonetheless, in situ electrophysiological measurements remain noisy, spatially constrained, and difficult to scale. Bridging the gap between laboratory precision and ecological complexity will require integrated, multiscale platforms that combine electrophysiology, imaging, remote sensing, and high-resolution environmental metadata. Such frameworks have the potential to transform our understanding of subterranean fungal activity and to enable the deployment of mycelial networks themselves as in-habitat biosensors embedded within natural ecosystems. In this perspective, the same mycelial networks that underpin decomposition, nutrient cycling, and symbiosis may also operate as autonomous, responsive interfaces capable of detecting, interpreting, and responding to environmental change. Artificial intelligence emerges as a unifying force across these domains. Machine-learning approaches already support prediction of fungal lifestyles, identification of key ecological drivers, interpretation of complex molecular interactions, and optimization of biosensing performance. In parallel, fungal systems have inspired new classes of metaheuristic and bioinspired algorithms modeled on hyphal foraging, mycorrhizal cooperation, and distributed resource allocation, illustrating a reciprocal relationship in which fungi both benefit from and inform advances in artificial intelligence. Taken together, the opportunities outlined in this review call for deeply integrative research strategies. Advances in fungal genomics, ecology, electrophysiology, and nanofabrication must converge with materials science, computational modeling, and systems engineering. To clarify the path from conceptual demonstrations to reliable and responsible technologies, the following paragraphs synthesize the key cross-cutting challenges that currently constrain fungal-based sensing across scales. Despite substantial progress, the translation of fungal-based (bio)sensing concepts into robust, deployable technologies remains constrained by several recurring challenges that span molecular, material, and system levels. A central issue across fungal enzymes, mycogenic nanomaterials, and living mycelial interfaces is reproducibility, as biological variability associated with strain selection, growth conditions, and physiological state can strongly influence sensing performance. Future work will likely benefit from more systematic reporting of biological provenance and experimental conditions, enabling clearer comparison across studies. Stability and signal drift represent additional limiting factors. Enzyme- and secretome-based sensors remain sensitive to denaturation, leaching, and mediator degradation, while mycelium-based materials exhibit intrinsic physiological dynamics and environmental sensitivity to humidity, desiccation, and contamination. Long-term operation, storage stability, and performance under repeated cycling remain insufficiently characterized in many reports, particularly under realistic matrices. At the materials and device level, scale-up and integration pose nontrivial challenges. Although mycogenic nanomaterials offer clear advantages in sustainability and functional diversity, their properties may vary with production scale, and correlations between biosynthetic conditions and device-level performance remain incompletely understood. Similarly, reliable electrical interfacing, encapsulation, and calibration strategies are critical for reducing device-to-device variability and enabling practical deployment. From an application perspective, selectivity and robustness in complex environments remain key hurdles. Many fungal-based sensors are validated under controlled laboratory conditions, whereas real-world samples introduce interferents, biofouling, and fluctuating physicochemical parameters. Addressing these effects will require validation against reference methods, comparative benchmarking with established sensor technologies, and testing in representative field conditions. Finally, safety, biocompatibility, and regulatory considerations merit greater attention, particularly for approaches involving living fungi, spores, or crude extracts. Allergenicity, potential toxin production, and contamination risks underscore the importance of containment strategies, post-processing or inactivation steps, and alignment with existing regulatory frameworks for environmental or human-adjacent sensing applications. Viewed together, these challenges do not diminish the promise of fungal-based sensing; rather, they help delineate the contexts in which fungi may offer distinctive advantages over conventional platforms—such as selective biorecognition, sustainable material synthesis, and adaptive, living interfaces—while also clarifying the steps required to progress from proof-of-concept demonstrations toward reliable and responsible real-world applications. As one of the oldest, most diverse, and most widely distributed kingdoms of life, fungi represent an extraordinary and forward-looking foundation for future (bio)sensing technologies—ranging from molecular recognition to living, adaptive sensing systems that extend beyond current technological paradigms. A visual overview of representative fungal species discussed in this review is provided in the Supplementary Materials (Figures S1–S9). These images are intended not only to illustrate the remarkable diversity and intrinsic beauty of these organisms, but also to underscore the respect they merit. As essential components of global ecosystems, fungi must be protected to safeguard the planet’s future. At the same time, these images invite broader reflection: the solutions to our current and future scientific challenges may already lie before our eyes—or just beneath our feet—as we walk quietly in the woods.

## Figures and Tables

**Table 1:** Classes of Fungal Enzymes.

Enzyme Class	General Characteristics	Examples/Applications
Oxidoreductases (EC 1)	Catalyze oxidation/reduction reactions by transferring electrons. Play a role in vital biological processes like the tricarboxylic acid cycle, glycolysis, and oxidative phosphorylation. Involved in fungal pathogenicity and protection against host defense mechanisms.	Dehydrogenases, oxygenases (like laccases), and peroxidases (like lignin peroxidase and manganese peroxidase).
Transferases (EC 2)	Catalyze the transfer or exchange of specific groups, such as an amino group, between compounds. Crucial for creating essential amino acids for protein synthesis.	Glutathione transferase (helps pathogenic fungi tolerate stress) and fructosyltransferase (converts sucrose into fructooligosaccharides).
Hydrolases (EC 3)	The most extensively studied and commercially marketed group of enzymes, which catalyze the hydrolysis of their substrates by adding water.	Proteases, amylases, lipases, and cellulases. Fungal cellulases are vital for degrading cellulosic agricultural waste.
Lyases (EC 4)	Catalyze addition or elimination reactions, often resulting in new compounds with a cyclic structure or new double bonds.	Pectin lyase (important for fungal pathogenicity in plants) and alginate lyase (degrades alginate).
Isomerases (EC 5)	Catalyze the rearrangement of a substrate’s structure by interchanging a specific group within the same compound.	Glucose/xylose isomerase, used to produce high-fructose corn syrup and biofuel.
Ligases (EC 6)	Catalyze the joining of two compounds by forming new bonds. Most are intracellular and modify cellular nucleic acid content.	Ubiquitin ligases (involved in marking proteins for degradation, activation, or relocation).
Translocases (EC 7)	Catalyze the movement of a substance across a membrane.	Adenine nucleotide translocase (ANT), which moves ADP/ATP across the mitochondrial membrane

**Table 2:** Electrochemical laccase-based biosensors.

Target Analyte Class	Specific Analyte(s)	Laccase Fungal Source	Electrode/ Sensitive Material	Detection Principle	Analytical Performance	Real Sample Application	Reference
Hormone	17β-estradiol	*Agaricus* *bisporus*	Glassy Carbon Electrode + L-lysine + Citric acid-functionalized graphene	Cyclic Voltammetry, Differential Pulse Voltammetry, Electrochemical Impedance Spectroscopy	Linear range: 4 × 10^−13^–5.7 × 10^−11^ M; LOD: 1.3 × 10^−13^ M.	Human urine samples	[90]
Neurotransmitter	Dopamine	*Agaricus* *bisporus*	Gold interdigitated electrodes on ceramic substrate	Conductometric	Linear range: 23 µM–1 mM; LOD: 7.8 µM; sensitivity: 11.7 µS mM^−1^	Pharmaceutical solutions, biological samples	[89]
Neurotransmitters	Dopamine	*Xylaria* sp.	Sodium trimetaphosphate–crosslinked chitosan/graphite incorporating laccase.	Cyclic Voltammetry, Square-Wave Voltammetry	Linear range: 0.17–492.83 µmol L^−1^; LOD: 0.17 µmol L^−1^; sensitivity: 0.30 µmol L^−1^	Synthetic body fluids; biomedical and environmental relevance	[85]
Neurotransmitters	Dopamine	*Aspergillus* *oryzae*	Laccase/ImS3–14–halloysite nanotubes in carbon paste electrode	Square-Wave Voltammetry	Linear range: 0.99–67.8 µmol L^−1^; LOD: 0.252 µmol L^−1^; RSD: 5.3%; Recovery: 97.6–108.7%	Pharmaceutical formulations	[88]
Neurotransmitters	Dopamine, L-Epinephrine	*Trametes* *pubescens*	Gold-modified glassy carbon electrode with cystamine monolayer and immobilized laccase	Amperometric	Dopamine: 0–0.12 mM; LOD 3.74 × 10^−8^ M; Sensitivity 0.178 A·L·mol^−1^·cm^−2^ L-Epinephrine: 0–0.19 mM; LOD 5.41 × 10^−8^ M; Sensitivity 0.123 A·L·mol^−1^·cm^−2^	Pharmaceutical injection solutions	[82]
Neurotransmitters	Dopamine	*Pycnoporus sanguineus* CS43 (LacI/LacII) and commercial *Trametes versicolor* laccase (TvL)	Carbon paper electrode modified with MoS_2_ nanoribbons; laccase was immobilized in Nafion/tributylammonium bromide film	Amperometric	Linear range: 1.33–13.32 µM (LacII); 4.00–19.96 µM (TvL); LOD: 0.67 (LacII), 2.67 µM (TvL); sensitivity: 15.36–17.87 nA µM^−1^ cm^−2^; Response < 3 s; RSD < 5%; 90% stability (10 days, 4 °C)	Sintetic urine	[83]
Phenolic compound	Catechol	*Trametes zonatus*	Laccase/NPs/ graphite electrode (CuCo, NiPtPd, PdHCF, AgHCF, PtHCF, PtCeHCF, AuHCF, AuCo)	Amperometric	Linear range: 0.5–50 µM; LOD: 0.16 µM; Sensitivity: 4523 A·M^−1^·m^−2^; Stability: 95% (10 days, 4 °C)	Wastewater and green tea extract	[87]
Phenolic compound	Hydroquinone	*Botryosphaeria rhodina* MAMB-05	CBPE/AuNPs/BOT/LCE	Square-Wave Voltammetry	Linear Range 2.00–56.5 µM LOD 0.474 µM Sensitivity 0.069 µA µM^−1^ Stability < 5% (25 days)	Dermatological cream; human urine; river water	[79]
Phenolic compound	Cathecol	*Trametes* *versicolor*	AuNPs–MoS_2_–Laccase/Nafion/Glassy carbon electrode	Differential Pulse Voltammetry	Linear Range: 2–2000 µM; LOD: 2 µM; Sensitivity: 0.0163 µA µM^−1^	Model wastewater	[75]
Phenolic compound	ABTS (model), catechol, hydroquinone, aminophenols (2-, 3-), dihydroxybenzaldehydes (2,4-; 2,5-; 3,4-), 2,6-dimethoxyphenol, syringaldazine	*Coriolus hirsuta* (also known as. *Trametes hirsuta*) laccase; both native (ChL) and aminated laccase (ChLa)	COOH-MWCNT/SPCE	Amperometric	ABTS: linear range 0.002–0.061 µM; sensitivity 831 nA µM^−1^. Other phenolic substrates: linear ranges in the sub-µM range (≈0.001–0.10 µM); LOD and sensitivity not systematically reported.	No real matrices were tested	[84]
Phenolic compound	Caffeic acid	*Trametes* *versicolor*	Polypyrrole–laccase–AuNPs film on screen-printed carbon electrode	Amperometric	Linear range: 1–250 µM; LOD: 0.83 µM; Sensitivity: 0.883 µA µM^−1^; Precision: <6.6%; Response time: ~15 min.	Propolis (ethanolic extracts)	[86]
Phenolic compound	p-Coumaric acid	*Trametes* *versicolor*	Laccase on cobalt phthalocyanine-modified carbon nanofiber screen-printed electrode	Amperometric	Linear Range: 0.009–100 µM. LOD: 0.003 µM. Sensitivity: 8.46 µA·µM^−1^·cm^−2^.	Phytoproducts	[81]
Phenolic compound	Bisphenol A	*Monilinia* *fructicola*	Polyaniline-modified platinum electrodes	Amperometric	Linear range: 0.03–0.25 mM; LOD: 1.9 µM; Sensitivity: 649 A·M^−1^·cm^−2^	Model solutions	[91]
Phenolic compound	BPA	*Trametes* *versicolor*	Multi-walled carbon nanotubes with chitosan and an ionic liquid	Amperometric	Linear range: 0.5 µM–12 mM; LOD: 8.4 nM; Sensitivity: 6.6 × 10^−2^ µA mM^−1^; Reproducibility: <6%; Stability: 87% (1 month, 4 °C)	River water	[80]
Cationic surfactants	Cetyltrimethyl- ammonium Bromide	*Aspergillus* sp.	Laccase/ZnO embedded in a polypyrrole–polyaniline film on a glassy carbon electrode	Differential Pulse Voltammetry	Linear ranges: 0.5–100/200–500/700–1900 µM; LOD: 0.0116 µM; Sensitivity: 0.935 µA·µM^−1^·cm^−2^	Tap water and sewage wastewater	[77]
Phenolic compound	Catechol	*Trametes* *versicolor*	Fe_3_O_4_–gold composite electrode functionalized with mercapto-undecanoic acid and laccase, assembled under magnetic field.	Amperometric	Linear range: 0.1–250 µM; LOD: 0.015 µM (S/N = 3); Sensitivity: 108.3 µA·mM^−1^·cm^−2^; Reproducibility: RSD 2.9%; Stability: 93% signal retained after 25 days; Response time: <4 s	Industrial wastewater	[78]
Aromatic N-nitrosamine	Dephostatin	*Trametes* *versicolor*	Zirconium dioxide–β-cyclodextrin–polyaniline composite on screen-printed carbon electrode.	Amperometric	Linear range: 0.05–100 nM; LOD/LOQ: 0.029/0.098 nM; Sensitivity: 234 µA·cm^−2^·µM^−1^; RSD: ≤2.5%; Response: 15 s.	Malted drinks and milk powder	[76]

**Table 3:** Photothermal and optical laccase-based biosensors.

Target Analyte Class	Specific Analyte(s)		Fungal Laccase Source	Sensitive Material	Detection Principle	Analytical Performances	Real Sample Application	Reference
Phenolic compound		Hydroquinone	*Trametes* sp. LS-10C	Nitrogen-doped carbon nanonets–laccase composite	Amperometric measurement under illumination	Linear range: 1–1000 µM (under illumination); LOD: 0.14 µM (light), 7.26 µM (dark); Response time: ~5 s; Selectivity: hydroquinone over BPA, chlorophenols, resorcinol; Interference tolerance: metal ions (Cr^3+^, Pb^2+^, Mg^2+^, Zn^2+^, Cu^2+^) and common organic solvents; Stability: 68.4% activity after 18 days; ≤3.5% signal loss after 50 CV cycles (pH 4.0–6.0); Optimal pH: 5.0.	Tap water and river water	[96]
Neurotransmitter		Dopamine	*Trametes* sp. LS-10C	Fe_3_O_4_@chitosan–gold–laccase composite	Amperometric enhanced by photothermal effect.	Linear range: 1–1000 µM (light) LOD: 0.79 µM (light); 4.45 µM (dark) Response time: ~0.2 s Optimal pH: 4.5 Selectivity: high vs. uric acid, ascorbic acid, glucose Interference tolerance: Cu^2+^, Zn^2+^, Mn^2+^, Pb^2+^, Ca^2+^, Mg^2+^; ethanol, methanol, acetone, DMSO, DMF Stability: −7.0% (oxidation), −3.6% (reduction) after 50 cycles	Synthetic urine	[97]
		Dopamine	*Trametes* *versicolor*	Functionalized carbon dots with immobilized laccase on tapered optical fiber	Fluorescence	Solution (phosphate-buffered saline, 10 mM, pH 7.4): Linear range: 0–0.4 µM; LOD: 41.2 nM; R^2^ = 0.995; high selectivity (no interference from common biomolecules or ions); stability: >95% fluorescence retained after 1 month. Optical fiber-based configuration: Linear range: 0–0.4 µM (tested up to 10 µM); LOD: 46.4 nM; R^2^ = 0.994; improved signal stability due to tapered fiber geometry and immobilization. Quantum yield: Carbon dots ≈14.8%; amino-functionalized carbon dots ≈12.3%; laccase-functionalized carbon dot bioprobe ≈10.2%	Human serum and cerebrospinal fluid	[98]
		Epinephrine (adrenaline)	*Trametes* *versicolor*	Laccase–Cu_3_(PO_4_)_2_· 3H_2_O hybrid microflowers	Colorimetric	Linear range: 0.4–400 µg mL^−1^ LOD: 0.1 µg mL^−1^ Repeatability: RSD 2.6% (*n* = 10, 1 µg mL^−1^) Relative activity: 112.5% vs. free laccase Stability: 96.6% retained after 30 days (4 °C) Reusability: 64.4% after 5 cycles Selectivity: no interference from common biomolecules	Human blood serum and urine	[99]
Laccase inhibitors		Cysteine, malic acid, fumaric acid	*Trametes* *versicolor*	Hollow microlayers with laccase immobilized on poly(acrylic acid)-modified magnetic nanocomposites	Fluorescence change induced by pH-responsive laccase release	Cysteine: LR 0.05–100 µmol L^−1^; LOD 0.01 µmol L^−1^; RSD 3.1–3.9% Malic acid: LR 0.02–100 µmol L^−1^; LOD 0.005 µmol L^−1^; RSD 2.8–5.1% Fumaric acid: LR 0.1–100 µmol L^−1^; LOD 0.02 µmol L^−1^; RSD 2.5–4.1%	Fruit juices (apple, pear, and grape)	[100]

**Table 4:** Fungal crude extract-based biosensors.

Target Analyte Class	Specific Analyte(s)	Fungal Source of Crude Extract	Sensitive Material	Detection Principle	Analytical Performances	Real Sample Application	Reference
Phenolic compounds	Tyrosine; phenol; catechol; caffeic acid; chlorogenic acid; L-DOPA	*Agaricus* *bisporus*	Crude enzyme extract	Colorimetric	LOD: 10^−6^–10^−5^ M; linear range: ≤10^−3^ M; long-term stability	Food supplements, synthetic serum, treated wastewaters	[114]
	Catechol, resorcinol, *p*-nitrophenol, 4-chlorophenol	*Marasmiellus* *colocasiae* (CCIBT 3388 strain)	Graphite–oil paste electrode	Differential Pulse Voltammetry	LOD: 0.17 µM; linear range: 50–300 µM; ~6× signal enhancement	Drinking water	[115]
	Catechol; gallic acid; caffeic acid	*Trametes* *pubescens*	Polypyrrole– enzyme composite	Amperometric	LOD: 1.8–5.0 µM Linear range: ≤70 µM Sensitivity: ≤37.5 µA·mM^−1^·cm^−2^	Fruit wines (blueberry, blackberry, and pomegranate)	[116]
Neurotransmitter precursor	L-DOPA	*Clitocybe* *nebularis*	Carbon paste electrode	Amperometric	LOD 0.76 µM; linear range 2.5–100 µM; Precision 2.7%	Synthetic serum and pharmaceutical formulations (commercial L-DOPA tablets)	[117]
Phenolic phytomarkers	Catechin and gallic acid	*Marasmiellus* *colocasiae* (CCIBT 3388 strain)	Carbon paste electrode	Differential pulse voltammetry	LOD 0.12–0.14 µM; RSD ≤ 8.4%	Green tea and kombucha beverages (*Camellia sinensis*)	[118]
Gas-phase analytes	Water vapor, ethanol vapor, and acetone vapor	*Ganoderma* *Lucidum* (strain 5.1)	Mycelium extract thin film on surface and acoustic plate wave device	Acousto- electronic	Response 140–150 s; stability ≥ 60 days	Not tested	[119]
	No gas detected (screening study)	*Ganoderma* *lucidum*	Mycelium extract thin film on metal/aluminum nitride/metal/diamond acoustic resonator	Acousto- electronic	Resonance ~2.75–3.0 GHz; Q-factor up to ~10^4^; thin films showed best Q and reproducibility (Δf quantified)	Not tested	[120]
Endocrine disruptor	Bisphenol A (BPA)	*Pleurotus* *ostreatus*	AuNP–ionic liquid composite	Amperometric	LOD 0.03 µM; linear range 0.1–100 µM; response ~6 s	Bottled water; milk; beverages	[121]
Mycotoxins	Aflatoxin M1	*Agaricus* *bisporus*	Carbon nanotube–graphene oxide–gold nanoparticle composite	Amperometric	LOD 10^−12^ M; linear range 10^−11^–10^−6^ M	Milk and dairy products	[122]

**Table 5:** Fungal HFBs-based biosensors.

Target Analyte Class	Specific Analyte(s)	HFBs	Detection Principle	Analytical Performances	Real Sample Application	Reference
Proteins/enzymes	Thrombin	HGFI from *Grifola frondosa*	Fluorescence	Linear range 1.07 aM–0.01 mM; LOD 0.2 aM; R^2^ 0.998; response < 10 min; high selectivity.	Serum	[131]
Herbicide	Glyphosate	Ccg2 from *Neurospora crassa*	Colorimetric (inhibition assay)	Linear range 0.05–1.0 µM; LOD 50 nM (8.45 ng mL^−1^)	None reported (proof-of-concept on laboratory solutions)	[132]
Herbicide	Glyphosate	Ccg2 from *Neurospora crassa*	Reflection Interference Contrast Microscopy	Linear range: 0.01 pM–10 nM; LOD: ~100 pM; response: ≤15 min; high selectivity; pentaglycine linker improves performance	No real matrices tested; validated in aqueous model systems	[133]
Volatile Organic Compounds (VOCs)	Methanol, Ethanol, Acetone, Tetrahydrofuran, Hexane	HFBI from *Trichoderma* *reesei*	Mass Loading	Response time: 16–18 s (ethanol); 9–13 s (hexane) Recovery time: ~30 s (ethanol); ~20 s (hexane) Sensitivity enhancement: ~8× (ethanol)	None; pure VOCs in controlled gas chambers	[134]
Phenolic compounds	L-DOPA, Caffeic acid	Vmh2 from *Pleurotus ostreatus*	Absorbance	L-DOPA (buffer): Linear range 5–1000 µM; LOD: ~3 µM L-DOPA (plasma): Linear range 10–1000 µM	Human plasma and Beverages (ACE juice, tea infusion)	[135]
Phenolic compounds/ neurotransmitters	Catechol and dopamine	Vmh2 from *Pleurotus ostreatus*	Amperometric	Catechol: 20–1000 µM; LOD 20 µM; Sensitivity 0.27 mA·M^−1^·cm^−2^ Dopamine: 20–250 µM; LOD 20 µM; Sensitivity 16.4 µA·M^−1^·cm^−2^	No real samples; validated in phosphate/citrate buffer (pH 5)	[136]
Phenolic compounds/neurotransmitters	Catechol and dopamine	Vmh2 from *Pleurotus* *ostreatus*	Amperometric	Catechol: 2–30 pM and 0.1–800 µM; LOD: 2 pM; Sensitivity: 2.36 × 10^4^/0.28 mA·L·mmol^−1^·cm^−2^	No real samples; validated in phosphate/citrate buffer (pH 5)	[137]
Metalloid	Arsenic (As(III), As(V))	Vmh2 from *Pleurotus ostreatus*	Square-wave voltammetry	Activity retained: up to 2.5 mU mg^−1^; surface loading: 4.3–6.4 pmol cm^−2^; K_As(III): 650–1200 L mol^−1^; stability: >15 days; reusability: 3 cycles	Tested in aqueous model systems only.	[138]
Heavy metal	Mercury (Hg^2+^)	Vmh2 from *Pleurotus* *ostreatus*	Fluorescence	Linear range: 1 nM–1 mM (log R^2^ > 0.99); LOD: 0.3–0.4 nM; selectivity: Hg^2+^ (Cu^2+^ interference mitigated)	Tap water and sea water	[139]
Marine neurotoxins	Saxitoxin (STX) and Domoic Acid (DA)	Vmh2 from *Pleurotus ostreatus*	Electrochemical and optical immunosensing (competitive binding assay)	DA: Linear range 0–2.5 ng mL^−1^; LOD 0.35 ng mL^−1^ (electrochemical); ~25% activity retained after 21 days (4 °C). STX: Electrochemical: 0–300 pg mL^−1^, LOD 52 pg mL^−1^ (R^2^ = 0.9845); Optical: 0–100 pg mL^−1^, LOD 1.7 pg mL^−1^ (R^2^ = 0.9879); ~40% functionality retained after 21 days (4 °C); ~100% immobilization efficiency on MBs.	No real environmental or food matrices tested	[140]
Bacterial cells	*Escherichia coli* and *Staphylococcus epidermidis*	Vmh2 from *Pleurotus ostreatus*	Colorimetric	Linear range 10^1^–10^5^ CFU mL^−1^ (*E. coli*, *S. epidermidis*, mixed samples); LOD 10 CFU mL^−1^ (*E. coli*), 48 CFU mL^−1^ (*S. epidermidis*), ~27 CFU mL^−1^ (ML-assisted); response time 15 min; recovery 80–110% (tap water, seawater, artificial saliva); ML accuracy 97 ± 1% (MAE ≈ 0.02, RMSE ≈ 0.04); reproducible over five replicates.	Tap water, sea water, and artificial saliva	[141]

**Table 6:** Fungal EPS-based biosensors.

Target Analyte Class	Specific Analyte(s)	EPS and Fungal Source	Sensitive Material	Detection Principle	Analytical Performance	Real Sample Application	Reference
Phenolic compound	Hydro- quinone	Botryosphaeran (*Botryosphaeria* *rhodina*)	Gold nanoparticles–laccase–EPS composite electrode	Square Wave Voltammetry	Linear range 2.0–56.5 µM; LOD 0.47 µM	Dermatological cream; human urine; river water	[79]
Food spoilage markers	TVB-N/ammonia; pH	Pullulan (*Aureobasidium* *pullulans*)	β-lactoglobulin–pullulan film incorporating anthocyanins	Colorimetric	Rapid color change (10–30 min); ΔE correlates with TVB-N; qualitative/ semiquantitative	Barramundi fish	[163]
Organic pollutant	4-nitrophenol	Lentinan (*Lentinus edodes*)	Lentinan-stabilized palladium nanozyme	UV–Vis	90% reduction in 21 min; k_app = 69.4 s^−1^ mM^−1^	Not tested	[164]
Carbohydrate	Glucose	Lentinan (*Lentinus edodes*)	Platinum nanoclusters immobilized on lentinan	Colorimetric	Linear range 5–1000 µM; LOD 1.79 µM	Human serum; urine	[165]
Amino acid	L-cysteine	Lentinan (*Lentinus edodes*)	Palladium–platinum dendritic nanoparticles immobilized on lentinan	Colorimetric	Linear range 0–200 µM; LOD 3.10 µM	Milk	[166]
Neurotransmitter/drug	Dopamine; spironolactone	Botryosphaeran (*Botryosphaeria* *rhodina*)	Laccase/EPS–multiwalled carbon nanotube–glassy carbon electrode	Square Wave Voltammetry	Dopamine: LOD 0.127 µM; response ≈ 2 s. Spironolactone: proof-of-concept detection; LOD/linear range not reported.	Pharmaceuticals; synthetic biofluids	[168]
Phenolic compound	2,6-dimethoxyphenol	Botryosphaeran (*Botryosphaeria* *rhodina*)	Zinc oxide quantum dots/laccase–EPS composite on glassy carbon electrode	Square Wave Voltammetry	Linear range 10–400 nM; LOD 9 nM	Food and environmental samples	[169]
Phenolic compounds	Dopamine (DOP); paracetamol (PAR)	Carboxymethyl- botryosphaeran	Carbon black/EPS composite on glassy carbon electrode	Differential Pulse Voltammetry	LOD 0.013 µM (DOP); 0.11 µM (PAR)	Pharmaceuticals; synthetic biofluids	[170]
Pharmaceutical compound	Desloratadine	Carboxymethyl- botryosphaeran	Multiwalled carbon nanotube/EPS composite on glassy carbon electrode	Linear Sweep Voltammetry	Linear range 1.49–32.9 µM; LOD 0.88 µM	Tablets; oral solutions; rat serum	[171]
Phenolic compounds (flavonoids)	Quercetin	Carboxymethyl- botryosphaeran	Laccase/EPS–carbon black paste	Square Wave Voltammetry	Linear range 5 × 10^−8^–5 × 10^−7^ M; LOD 2.6 × 10^−8^ M	Beverages; urine; pharmaceuticals	[172]

**Table 7:** Fungal lectin- and aegerolysin-based biosensors.

Target Analyte Class	Specific Analyte(s)	Fungal Lectin- and Aegerolysin Source	Sensitive Material	Detection Principle	Analytical Performance	Real Sample Application	Reference
Carbohydrates (disaccharides)	Lactose	*Agaricus bisporus* lectin (from crude mushroom extract)	*Agaricus bisporus* lectin immobilized on poly(methylene blue)-modified fluorine-doped tin oxide photoelectrode	Photoelectrochemical	Linear range: 0.001–300 µM; LOD: 0.001 µM; Sensitivity: 3.05 µA µM^−1^ cm^−2^; Response time: 10 s; R^2^: 0.998; Stability: >95% (15 days); Reproducibility: RSD < 3% (*n* = 10); Selectivity: no interference from glucose, maltose, sucrose.	Milk (cow, goat, and infant formula)	[175]
Carbohydrates (monosaccharides)	Glucose	*Ganoderma applanatum* lectin (purified)	*Ganoderma* *applanatum* lectin immobilized on thermally activated Prussian blue-modified glassy carbon electrode	Square-wave voltammetry and electrochemical impedance spectroscopy	Linear range: 0.08–85 nM; LOD: 10.2 pM; LOQ: 34.6 pM; Sensitivity: 0.012 µA µM^−1^ cm^−2^; R^2^: 0.993 (Hill fit); Stability: 93.5% (20 cycles); Precision: RSD < 4.5%; Reproducibility: RSD 6.3%; Selectivity: minor interference from fructose (7.3%) and sucrose (13.2%)	Pharmaceutical glucose formulations	[176]
Membrane lipid components	Phosphatidic acid, cardiolipin, sphingomyelin, cholesterol–sphingolipid complexes	Recombinant aegerolysin/MACPF pairs: *P. ostreatus* (OlyA6/PlyB), *L. nuda* (NudA/NudB), *H. irregulare* (HetA/HetB), *M. mucida* (MucA/MucB), *T. versicolor* (VerA/VerB, Δ37)	Aegerolysin/MACPF complexes	Spectrophotometric	Specificity: PA/CL (pH 6.0); sphingolipid-selective membranes. Hemolysis (1 µM): OlyA6/PlyB ≈ 0.7 min; NudA/NudB ≈ 3.0 min. pH effect: reduced activity at pH 7.0–8.0.	Sf9 insect cells	[177]

**Table 8:** Myconanosynthesis for biosensoristics and optoelectronics.

Nanomaterial	Nanomaterial Characteristics	Fungal Source	Target Analyte Class	Specific Analyte(s)	Detection Principle	Analytical Performance	Application	Reference
Gold nanoparticles (AuNPs)	Spherical 9–93 nm, SPR 525–550 nm, stable (−1.9 to −29.9 mV zeta potential)	*Botrytis cinerea*, *Trichoderma atroviride*, *Trichoderma asperellum*, *Alternaria* sp., *Ganoderma sessile*	Raman-active organic dye	Methylene blue	Surface-enhanced Raman spectroscopy (SERS)	Enhancement factors 6.9–35.5 depending on fungal species	SERS substrates for trace molecule detection and biosensing	[179]
Silver/silver oxide nanoparticles (Ag/Ag_2_O NPs)	Protein-capped Ag/Ag_2_O NPs (5–10 nm), face-centred cubic, water-stable	*Fusarium* *oxysporum*	Carbohydrate	D-glucose	Cyclic voltammetry	Linear response over 25–125 µM glucose with R^2^ = 0.995; high reproducibility and stability	Enzyme-free glucose sensing; methylene blue degradation; antimicrobial activity	[180]
Core/shell silver nanoparticles (F-AgNPs)	Spherical Ag/Ag_2_O NPs (5–10 nm), face-centred cubic structure, protein-capped, water-stable	*Agaricus* *bisporus*	N.a. *	N.a. *	N.a. *	CdS conductivity enhanced (289 → 172 Ω) with preserved optical transparency (>70%).	CdS conductive coating with potential applicability in optoelectronic and biohybrid sensing interfaces enabled by melanin semiconducting behavior	[181]
Laccase Nanoparticles (LacNPs)	Spherical (~152 nm TEM; 191 nm hydrodynamic, PDI 8.6%), stable and non-aggregated (ζ = −38 mV); preserved protein secondary structure confirmed by FTIR (amide I/II bands).	*Agaricus* *bisporus* (commercial enzyme)	Phenolic compounds	Guaiacol (model phenolic substrate); total phenolics	Amperometric	Linear ranges: 0.1–600 µM; LOD: 0.3 µM; response time: 3 s; recovery: 92–98%; precision ≤ 3.4%; stability: 150 days	Determination of total phenolic content in tea leaves, alcoholic beverages, and pharmaceutical samples; environmental and food-quality monitoring	[182]
Laccase nanoparticles (Lac-NPs)	Spherical nanoparticles (~150–170 nm), ζ-potential −38 mV; stable, non-aggregating; protein structure preserved; cysteine-functionalized.	*Ganoderma lucidum* MDU-7	Neurotransmitters (catecholamines)	Dopamine, adrenaline, noradrenaline	Amperometric	Linear range: 0.1–800 µM; LOD: 0.12 µM; sensitivity: 2320 µA mM^−1^ cm^−2^; R^2^ = 0.999; recovery: 94–99%; precision: 1.6% (intra-day), 3.8% (inter-day); stability: 210 days	Determination of catecholamines in pharmaceutical formulations; potential for clinical and environmental monitoring	[183]
Gadolinium-doped zinc sulfide quantum dots (ZnS:Gd)	Spherical, monodispersed ZnS nanocrystals (10–18 nm), hexagonal phase, protein-capped, with enhanced fluorescence efficiency	*Aspergillus flavus* (endophytic fungus isolated from *Nothapodytes foetida*)	Heavy metals	Pb^2+^, Cd^2+^, Hg^2+^, Cu^2+^, Ni^2+^	Fluorescence	Qualitative metal-ion sensing via fluorescence enhancement (Pb^2+^/Cd^2+^) and quenching (Hg^2+^/Cu^2+^/Ni^2+^) at 100 µM	Fluorescence-based heavy metal ion detection in water; potential for environmental and luminescent sensors	[184]
Ruthenium oxide quantum dots (RuO_2_ QDs)	Nearly spherical, monodispersed (1–5 nm; ~3 nm); band gap 2.7 eV; fluorescence emission at 475 nm; fungal protein capping (FTIR amide I/II); low crystallinity (XRD).	*Fusarium oxysporum* (endophytic fungus)	Reactive oxygen species	Hydrogen peroxide (H_2_O_2_)	Colorimetric	Linear range: 10^−2^–10^−6^ M; LOD: 0.39 µM (9:1 RuO_2_ QDs:H_2_O_2_); assay time: 30 min; R = 0.99; reproducible across tested ratios	Reagent-free colorimetric detection of H_2_O_2_ in aqueous and spiked human plasma samples; applicable to diagnostic and environmental monitoring	[185]

* Not available.

**Table 9:** Myconanosynthesis of CQDs.

CQDs	CQDs Characteristics	Fungal Source	Target Analyte Class	Specific Analyte(s)	Detection Principle	Analytical Performances	Applications	Reference
Nitrogen, phosphorus co-doped carbon dots (Gl N,P-CDs) and undoped Gl CDs	Spherical carbon dots (≈2–3 nm) with excitation-dependent fluorescence; quantum yield 3.54% (Gl CDs) and 11.41% (Gl N,P-CDs); water-stable, N/P surface functionalization confirmed by XPS and FTIR.	*Ganoderma* *lucidum* (spore powder)	Nitroaromatic pollutants	2,4-dinitrophenol (2,4-DNP), 4-nitrophenol (4-NP)	Fluorescence quenching via inner filter effect	Linear range (µM): Gl CDs, 0–37.5 (2,4-DNP), 0–50 (4-NP); Gl N,P-CDs, 0–30 (both). LOD (nM): 89.77 (2,4-DNP), 100.27 (4-NP) for Gl CDs; 73.03 (2,4-DNP), 68.09 (4-NP) for Gl N,P-CDs.	Nitrophenol detection in water/soil; multicolor cellular and in vivo imaging.	[186]
Carbon quantum dots (CQDs)	Spherical CDs (3–8 nm); blue fluorescence (Ex 360 nm/Em 440 nm); QY 11.5%; −16.92 mV; –OH/–COOH/–NH_2_ surface.	*Volvariella* *volvacea*	Heavy metal ions	Fe^3+^, Pb^2+^	Fluorescence quenching	Linear range 1–100 µM; LOD 16 nM (Fe^3+^) and 12 nM (Pb^2+^); response ≤ 2 min; high selectivity; stable fluorescence under varying conditions	Detection of Fe^3+^ and Pb^2+^ in real water samples (tap, drinking, groundwater)	[187]
Carbon quantum dots	Spherical (5–10 nm); blue fluorescence (λ_ex 360 nm/λ_em 450 nm); excitation-dependent emission; hydrophilic –OH/–COOH/–NH_2_ surface; photostable; well-dispersible	*Pleurotus* *ostreatus*	Heavy metal ions	Pb^2+^ and Cr^6+^	Fluorescence quenching	Linear ranges: 10–1000 µM (Pb^2+^), 10–1000 µM (Cr^6+^). LOD: 1.24 µM (Pb^2+^), 2.34 µM (Cr^6+^).	Fluorescent detection of Pb^2+^ and Cr^6+^ in aqueous samples; antibacterial activity against *E. coli* and *S. aureus*; anticancer effects in MCF-7 cells	[188]
Carbon quantum dots (CQDs)	Blue-emissive CDs (~6 nm); quasi-spherical; excitation-dependent emission; high photostability; –OH/–NH_2_/–COOH surface enabling metal coordination	*Lentinus* *polychrous* Lèv	Heavy metal ions	Fe^3+^	Fluorescence turn-off sensing via inner filter effect (IFE) with dynamic and static quenching.	Linear range 0–2.0 mM (solution) and 0.2–1.0 mM (paper strip); LOD 16 µM; high selectivity for Fe^3+^; stable under UV/visible light and tolerant to NaCl and PBS.	Environmental monitoring of Fe^3+^ in water; portable paper-based fluorescence sensor	[189]
Carbon quantum dots (CQDs)	Blue-emissive CQDs (~4.6 nm); spherical, monodisperse; excitation-dependent PL; –OH/–COOH/–NH_2_ surface; high aqueous stability; QY ~4.8%	*Poria cocos* (alkali-soluble *Poria cocos* polysaccharide)	Heavy metals	Cr(VI), Cr(VI)	Fluorescence on–off sensing via inner filter effect (IFE) and static quenching	Linear range 1–100 µM; LOD 0.25 µM; high selectivity; stable across pH 1–13; good salt tolerance	Quantification of Cr(VI) in real water samples (tisanes, rainwater, river water)	[190]
Carbon Quantum Dots integrated with Ag nanoparticles (C-dots-AgNPs)	Hydrothermal synthesis from *Pleurotus* spp.; spherical, fluorescent; –OH/–COOH/C=O/–NH_2_-rich surface; enables in situ AgNP formation; size 6–8 nm; ζ-potential −65 mV; absorption at 269 and 449 nm	*Pleurotus* spp.	Polycyclic aromatic hydrocarbons (PAHs)	Anthracene and naphthalene	Cyclic voltammetry and square-wave voltammetry	Anthracene: 250 nM–1.15 mM, LOD 112 nM; naphthalene: 500 nM–842 µM, LOD 383 nM; simultaneous detection via well-separated oxidation peaks	Detection of PAHs in environmental samples (marine soil, seawater, crude oil, reused cooking oil)	[191]

**Table 10:** Studies on the bioelectrical properties of fungi.

Fungal Species	Recording Method	Spike Characteristics	Key Findings	Reference
*Pleurotus djamor*	Extracellular electrical potential recording via subdermal needle electrodes (stalk–cap); differential acquisition at 1 sample/s over multi-day monitoring	Two spontaneous spike types: high-frequency spikes (~0.88 mV, ~115 s, ~2.6 min period) and low-frequency spikes (~1.3 mV, ~143 s, ~14 min period); spikes occur in trains; evoked spikes up to ~6 mV	Fruiting bodies generate spontaneous action-potential-like spikes and distinct oscillatory modes; stimulus-induced responses propagate across clusters, indicating coordinated internal electrical signaling	[204]
	Extracellular electrical activity recorded via paired iridium-coated stainless-steel needle electrodes (1–2 cm spacing) in mycelium-colonized substrate; acquisition with 24-bit ADC at 1 sample/s	Action-potential-like spikes (0.5–6 mV); typical duration ~402 s; high- and low-frequency spike trains; refractory period ≥ 60 s; propagation over ~2 cm	A dedicated spike-detection algorithm distinguishes true spikes from noise; electrical activity exhibits measurable complexity, indicating coordinated electrical signaling in mycelium	[205]
*Ganoderma resinaceum*	Extracellular differential recordings via paired iridium-coated stainless-steel needle electrodes (1–2 cm spacing) in antler-like sporocarps; acquisition at 1 sample/s using a 24-bit ADC data logger.	Spike amplitudes mainly 0.1–0.4 mV (most <4 mV); spike widths typically 300–500 s; multiple spike types observed (single, compound, trains, oscillatory, long bursts); rare multi-hour bursts with ~70 spikes.	Electrical spiking in *G. resinaceum* shows species-specific temporal patterns distinct from *Pleurotus djamor*; spike widths correspond to a propagation speed of ~0.028 mm/s, comparable to fast calcium waves, indicating physiological electrical signaling	[206]
*Omphalotus nidiformis*, *Flammulina velutipes*, *Schizophyllum commune*, *Cordyceps militaris*	Extracellular differential recordings via iridium-coated stainless-steel needle electrodes inserted into colonized substrates or sporocarps; sampling at 1 Hz over multi-day periods using a 24-bit ADC (ADC-24)	Species-specific spike durations (1–21 h) and amplitudes (0.03–2.1 mV); mean inter-spike intervals ~0.5 h (*S. commune*) to ~2 h (*C. militaris*); spikes form trains with low-/high-frequency modes; occasional synchronized spiking across neighboring sporocarps	Electrical spiking patterns show structured temporal organization; spike-train word-length distributions resemble those of human languages, and state-transition analyses indicate non-random, species-specific spiking repertoires, with *S. commune* exhibiting the highest complexity.	[207]
*Ganoderma lucidum*	Extracellular electrical potential recordings in mycelium-bound composite blocks via Pt/Ir needle electrodes; ±5 V square-wave stimulation (100 Hz–10 kHz) applied through colonized substrate; signals sampled at 50 kHz	Transmission of frequency-modulated electrical signals across mycelium; irregular, sawtooth-like output waveforms with harmonics; recoverable frequencies detected in most samples (up to 100% in low–mid ranges); signals often non-stationary	Mycelium propagates external electrical signals across connected blocks with partial recovery of input frequency; Granger and NARX analyses indicate input–output dependence and approximate transfer functions, supporting feasibility of fungal-based analogue signal processing	[208]

**Table 11:** Studies on the use of fungi in unconventional computing and bioelectronic devices.

Fungal Species	Functional Electronic Components	Computational Principle	Findings and Limitations	Reference
*Pleurotus* *ostreatus*	Logic circuits implemented in living mycelium-bound composites	In materio computation via nonlinear electrical signal transformation, enabling Boolean function extraction from voltage spike responses	Mycelium composites implemented 470 of 3136 Boolean functions, including NAND, OR, AND, and rules across Wolfram classes I–IV; however, ongoing growth and structural reconfiguration limited repeatability, with improved stability after functionalization or drying	[210]
	Capacitors (intrinsic and voltage-dependent pseudocapacitance); charge-storage elements; hybrid organic electronic components	Computation via capacitive charge storage and release, exploiting voltage- and frequency-dependent pseudocapacitance and ionic–protonic conduction in hyphal networks	Mycelium exhibited pico- to microfarad-scale (pseudo)capacitance with non-ideal, diffusion-limited impedance behavior; however, electrical responses were strongly moisture-dependent, with drying and high voltages causing signal loss and potential hyphal damage, limiting use for stable energy storage	[214]
	Photosensor (PEDOT:PSS-functionalized fruiting body); memfractive element (combined memristive–memcapacitive behavior); organic hybrid photodetector	Computation via light-triggered current modulation, exploiting memfractive I–V behavior and hybrid ionic–electronic conduction enhanced by PEDOT:PSS	Unmodified mycelium and fruiting bodies showed no rapid electrical response to light despite memfractive behavior; PEDOT:PSS functionalization enabled strong, immediate light-synchronized current spikes, but moisture-dependent signal degradation limited stability and long-range conductivity	[215]
*Lentinula edodes* (Shiitake)	Memristors (volatile and non-volatile); capacitive, memfractive, and resistive components from dehydrated–rehydrated mycelium; mycelium-based RAM elements operating in the kHz range	Computation via memristive switching with pinched hysteresis loops, exploiting volatile memory from asymmetric resistance states and frequency-dependent retention	Mycelium composites exhibited near-ideal low-frequency memristive behavior with volatile memory retained up to ~5.85 kHz and stimulus-dependent capacitive, memfractive, and memristive responses; however, large sample variability, bulk device geometry, reduced high-frequency stability, and unoptimized growth conditions limited performance and reproducibility	[216]
Aerial mycelium from Ecovative’s proprietary *core foam strain* (filamentous Basidiomycete; exact species undisclosed).	PEDOT:PSS-infused mycelium sheets; nonlinear resistive–capacitive elements; physical reservoirs for analog signal transformation	Physical reservoir computing exploiting morphology-dependent nonlinear conduction and fading-memory dynamics of mycelium.	Mycelium reservoirs showed nonlinear I–V behavior, time-dependent responses with strong autocorrelation, and short-term memory sufficient for NARMA-10 prediction (NRMSE ≈ 0.98); however, moisture-induced signal drift, biological variability, and modest computational performance relative to electronic reservoirs limited reliability.	[217]
Not applicable (synthetic mycelium-inspired architecture; no biological fungus used)	Memristive oscillating cellular automata (MOCA) grid; SiNx-based MIS RRAM devices (1T1R configuration); reconfigurable oscillatory network emulating mycelial connectivity	Reservoir computing via nonlinear oscillatory cell dynamics and memristive, state-dependent connectivity, with mycelium-like morphological evolution encoded as RRAM-based small-world networks	The MOCA reservoir exhibited small-world topology (path length ≈ 1.175; clustering ≈ 0.756) and stable SiNx-based MIS RRAM switching with high endurance (~1400 cycles), enabling efficient temporal-to-high-dimensional state transformation; however, its synthetic (non-biological) architecture and the need for further large-scale optimization limited biological relevance and scalability	[219]

**Table 12:** Overview of functional fungal living materials.

Fungal Species	Fungal Living Material	Functional Outcomes	Limitation and Challenges	Reference
*Pleurotus ostreatus*	Hemp fabric colonized by actively growing mycelium; thin mycelium–textile composite	Stimulus-specific electrical responses to chemical and mechanical inputs; discrimination of stimuli via spike amplitude, frequency, and temporal dynamics; distributed sensory matrix for wearable bioelectronics	Strong moisture dependence and rapid desiccation-induced signal loss; performance degradation outside controlled humidity; spatial heterogeneity of electrical, mechanical, and chemical responses; risk of electrolysis and hyphal damage at high voltages; limited long-term durability and environmental robustness.	[221]
	Mycelium-colonized capillary matting; molded into full-size insoles	Mechanoresponsive electrical spiking under applied load; discrimination of pressure distributions (uniform, heel-, toe-loaded); excitation patterns suitable for pressure mapping	Low spike frequency limiting real-time gait analysis; moisture dependence; signal variability; contamination risk; substrate mechanical properties affecting stability	[222]
	Hemp shavings; nonwoven hemp fiber mats; mycelium-colonized	Steroid-induced modulation of mycelial electrical spiking; systematic changes in spike complexity and internal structure; hormone-responsive biosensing capability	Strong sensitivity to moisture and substrate ageing, leading to increased noise and inter-channel variability; subtle CT-detected structural changes requiring advanced analysis; unresolved dose–response relationships and limited specificity to hydrocortisone	[223]
*Ganoderma lucidum*	Premature mycelium skin; thin interconnected hyphal mat; chemically treated (alkaline/acidic) chitin–chitosan network	Enhanced mechanical strength and modulus; reduced surface roughness enabling metal film deposition; thermal stability up to 250 °C; high biodegradability; compatibility with flexible electronics (copper circuits, strain sensors, microstructured features, NFC tags) with durable conductivity under repeated bending	Intrinsic hygroscopicity affecting electrical behavior; need for chemical post-processing for surface uniformity; dissolution in strong acids; variability in mechanical properties of untreated material; current-induced thermal constraints with shellac coatings	[224]

**Table 13:** Fungal skin-based biohybrid sensing interfaces and functional characteristics.

Fungal Species	Fungal Skin	Functional Outcomes	Limitation and Challenges	Reference
*Ganoderma* *resinaceum*	Thin, flexible mycelial skin produced by static liquid culture; homogeneous ~1.5 mm living sheet; polyurethane-supported	Endogenous and stimulus-specific electrical activity; discrimination of mechanical and optical stimuli via distinct spiking signatures; coordinated multi-electrode responses enabling multimodal sensory integration.	High humidity requirement for viability; slow tactile response times with high variability; partial non-responsiveness across electrode pairs; long saturation and relaxation times for optical stimuli; sensitivity to electrode placement; unresolved long-term stability and scalability under dynamic environments	[225]
*Ganoderma* *sessile*	Living fungal skin grown directly on a cyborg-model surface; continuous mycelial coating on agar-primed substrate.	Cohesive, self-regenerating biofilm with fast and slow electrical spiking; stimulus-dependent responses including illumination-induced potential drift and tactile-evoked spikes; reactive bioelectronic interface capability	High humidity requirement for viability and electrical activity; strong dependence of signal amplitude and patterns on electrode placement and local hyphal structure; uncertain long-term stability under continuous mechanical movement and environmental fluctuations	[227]
*Ganoderma lucidum* (strain GL-M9726)	Pure mycelium skin produced by liquid-state fermentation; homogeneous leather-like pellicle of aerial and floating hyphae; enriched with thick-walled chlamydospores.	Dormancy-enabled viability via chlamydospores; robust self-healing after activation with restoration of mechanical integrity; post-healing shift toward increased hydrophobicity.	Material fragility and thickness variability requiring optimization; non-localized regrowth from widespread chlamydospore germination; reduced viability with high glycerol content and drying above 40 °C; environmental sensitivity, contamination risk, and unwanted regrowth; uncertain long-term stability under coatings, washing, and mechanical stress	[228]

**Table 14:** Studies on mycelium-based building materials and composites.

Fungal Species	Mycelium-Based Building Materials and Composites	Functional Outcomes	Limitation and Challenges	Reference
*Ganoderma* *resinaceum*	Large structural mycelium composites grown on hemp–soy substrate; block elements (20 × 20 × 10 cm)	Distinct electrical responses to mechanical loading and unloading; ON/OFF states discriminated by spike amplitude and duration; habituation under repeated loading and increased baseline spiking under sustained load	Spatial variability of electrical responses across electrodes; requirement for continuous moisture to sustain electrophysiological activity; habituation-induced signal attenuation under repeated stimuli; loss of electrical responsiveness upon desiccation	[230]
*Pleurotus ostreatus*, *Hericium erinaceus*	Mycelium-bound composites grown on rye and millet substrates; fresh or partially dried blocks; exposed or partially enclosed mycelium surfaces	Moisture-dependent electrical activity enabling humidity sensing; spontaneous spiking during dehydration and water-triggered high-amplitude responses; depth-dependent activity patterns supporting multilayer sensing in composite panels	Strong moisture dependence requiring controlled hydration; variability from heterogeneous commercial substrates; sensitivity to electrode placement and spacing; loss of electrical activity upon full dehydration; batch-to-batch variability and colonization-depth-dependent signal strength	[231]
*Ganoderma lucidum*	Living mycelium–polymer entangled composites formed by mycelium-induced phase separation; mycelium–PVA composites (MPCs) and CNT-assembled composites	High mechanical performance and toughness; robust self-healing with recovery of structural integrity; low water absorption and long-term regenerative capacity; enhanced load distribution via mycelium–polymer interfacial reinforcement	Strong dependence on cultivation conditions and active metabolism requiring environmental control; growth-stage-dependent variability in phase separation and network entanglement; scalability limited by growth non-uniformity; long-term stability dependent on biological activity and moisture management.	[232]

**Table 15:** Summary of Fungal-Inspired Metaheuristic Algorithms.

Algorithm	Fungal-Based Inspiration	Features	Limitation and Challenges	Reference
Discrete Mycorrhiza Optimization Algorithm (DMOA)	Mycorrhizal symbiosis with plant roots (resource exchange, defense signaling, competitive colonization)	Stochastic metaheuristic based on discrete Lotka–Volterra dynamics; dual plant–fungus populations; cooperative, competitive, and predatory interaction modes; random mode switching for enhanced exploration	Inferior performance to MTOA in most statistical comparisons; sensitivity to parameter tuning; occasional stagnation requiring diversification or restarts; validation limited to mathematical benchmarks without demonstrated real-world applications	[252]
Continuous Mycorrhiza Optimization Algorithm (CMOA)	Mycorrhizal network behavior enabling cooperative, competitive, and defense interactions in symbiotic resource-sharing systems	Continuous Lotka–Volterra modeling of plant–fungus population dynamics; integrated defense, competition, and cooperation operators; balanced exploration–exploitation behavior	High computational cost from ODE-based integration; sensitivity to parameter settings and initial conditions; validation limited to mathematical benchmarks without demonstrated real-world applications	[256]
	Plant–mycorrhizal ecological interactions, including defense, cooperative resource exchange, and competitive colonization, abstracted as population-interaction dynamics.	Dual interacting plant–fungus populations; stochastic predator–prey, cooperative, and competitive operators derived from Lotka–Volterra dynamics; probabilistic operator switching to maintain diversity and avoid local minima	Performance constrained by the No-Free-Lunch theorem; validation limited to a subset of benchmark functions; convergence dependent on parameter settings and population renewal; robustness in high-dimensional and real-world problems not yet established	[257]
Discrete Mycorrhiza Optimization Algorithm (DMOA)	Plant–fungal mycorrhizal symbiosis modeled as predator–prey, cooperative, and competitive population dynamics	Discrete Lotka–Volterra updating of interacting plant–fungus populations; alternating defense, cooperation, and competition operators; balanced exploration–exploitation dynamics	Slower and less precise than the continuous CMOA variant; strong dependence on parameter tuning and initial conditions; validation limited to mathematical benchmarks without real-world applications	[258]
Mycorrhized Tree Optimization Algorithm (MTOA)	Tree–mycorrhizal symbiosis involving defense signaling, cooperative nutrient exchange, and competitive colonization.	Discrete Lotka–Volterra modeling of tree–fungus populations; alternating defense, cooperation, and competition modes; balanced exploration–exploitation dynamics	Validation limited to mathematical benchmarks; lack of demonstrated real-world applications; increased computational cost and parameter sensitivity due to nonlinear differential equation solving	[259]
Fungal Growth Optimizer (FGO)	Hyphal tip extension, lateral branching, and spore germination driving fungal foraging and adaptive expansion.	Population-based optimizer with growth-, branching-, and spore-inspired operators controlling exploration and exploitation	Requires parameter tuning for stability; stochastic operators increase variance; computational cost scales with problem size	[261]
Bioluminescent Fungi Optimization Algorithm (BFOA)	Spore dispersal in bioluminescent fungi via insect attraction to fungal light.	Dual-agent system (fungi and insects); fitness-driven movement strategies; adaptive control of exploration and exploitation	Multiple fixed parameters require tuning; exploratory phase increases computational cost; performance sensitive to the fungi–insect ratio	[262]
The Fungi Kingdom Expansion (FKE) Algorithm.	Expansion behavior of filamentous fungi via hyphal extension, cytoplasmic flow toward favorable conditions, and stochastic spore germination under resource scarcity	Chaotic local search modeling immobile biomass expansion; deterministic movement toward locally optimal hyphal tips for mobile biomass; random spore-inspired redistribution of poorly performing solutions.	Increased memory demand from multi-hypha local search; need for careful tuning of expansion, environmental, and population parameters; validation limited to single-objective problems, with extension to multi-objective and higher-dimensional tasks required	[263]

## Data Availability

No new data were created or analyzed in this study. Data sharing is not applicable to this article.

## Supplementary Materials

The following supporting information can be downloaded at: https://www.mdpi.com/article/10.3390/bios16020131/s1, Figure S1: *Trametes ochracea*—extracellular redox-active fungal system. Photograph courtesy of Enzo Ferri; Figure S2: *Agaricus bisporus*—copper-enzyme-rich fungal system. Photograph courtesy of Antonio Lavagno; Figure S3: *Trametes versicolor*—redox-active and lipid-binding fungal system. Photograph courtesy of Antonio Lavagno; Figure S4: *Mucidula mucida*—lipid-binding fungal system. Photograph courtesy of Antonio Lavagno; Figure S5: *Lepista nuda*—lipid-binding fungal system. Photograph courtesy of Antonio Lavagno; Figure S6: *Armillaria bulbosa*—early model for fungal bioelectrical studies. Photograph courtesy of Amedeo Schipani; Figure S7: *Schizophyllum commune*—biomimetic and information-processing fungal system. Photograph courtesy of Enzo Ferri; Figure S8: *Pleurotus ostreatus*—multifunctional living fungal system. Photograph courtesy of Antonio Lavagno; Figure S9: *Ganoderma lucidum*—multifunctional biohybrid fungal system. Photograph courtesy of Antonio Lavagno.
