# Supplementary material for: Fungal Frontiers in (Bio)sensing
Source: Biosensors (Basel). 2026 Feb 22;16(2):131. doi: 10.3390/bios16020131 (PMC12938827; doi:10.3390/bios16020131)
Supplement: Supplementary file 1 [file biosensors-16-00131-s001.zip › biosensors-4148500-supplementary.pdf]

Review

Fungal Frontiers in (Bio)sensing

Gerardo Grasso [1,2,*]

[1] Istituto per lo Studio dei Materiali Nanostrutturati, Sede Roma-Sapienza, Consiglio Nazionale delle Ricerche, P.le Aldo Moro 5, 00185 Rome, Italy
[2] Nuova Micologia – Associazione di Studi Micologici – NPO, Viale dello Scalo S. Lorenzo, 16, 00185 Rome (RM), Italy
* Correspondence: gerardo.grasso@cnr.it

Supplementary Materials -Visual overview of representative fungal species

The following figures provide a visual overview of representative fungal species discussed in this review. Captions are intentionally concise and highlight conceptual traits relevant to biosensing, while the images are intended to convey the remarkable diversity and intrinsic value of these organisms and remain conceptually linked to the biosensing-related themes developed in the main text.

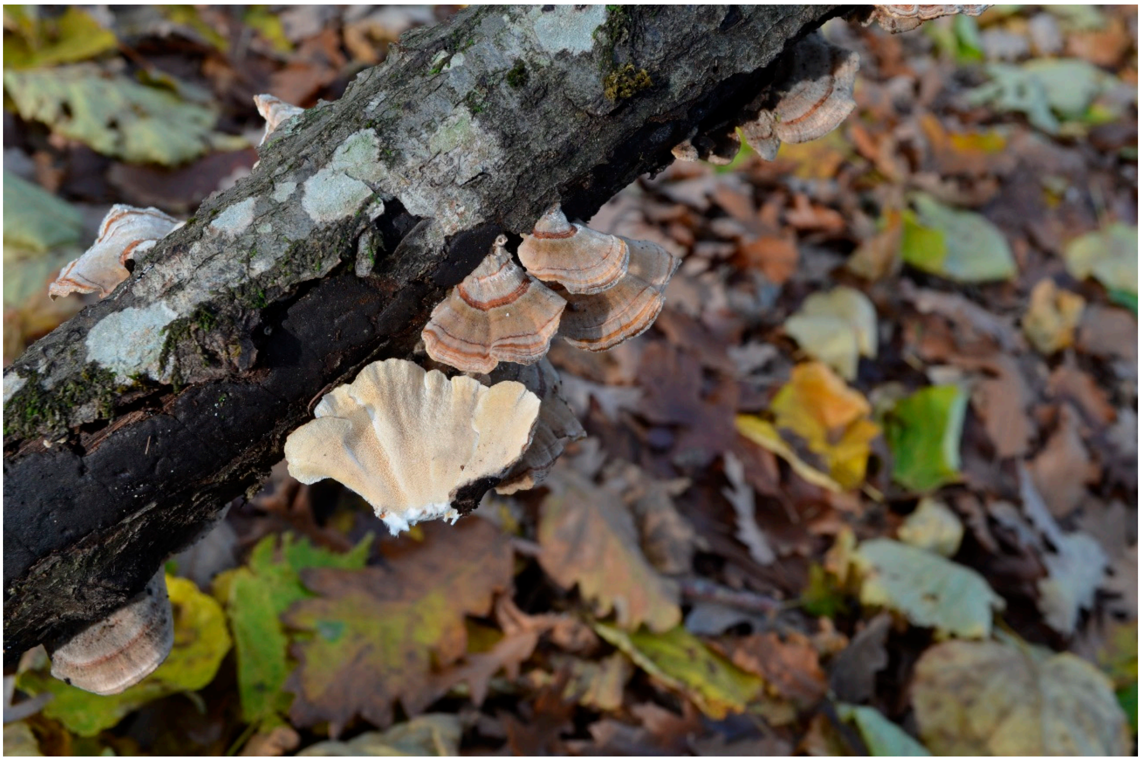

Figure S1. *Trametes ochracea* — extracellular redox-active fungal system. Photograph courtesy of Enzo Ferri.

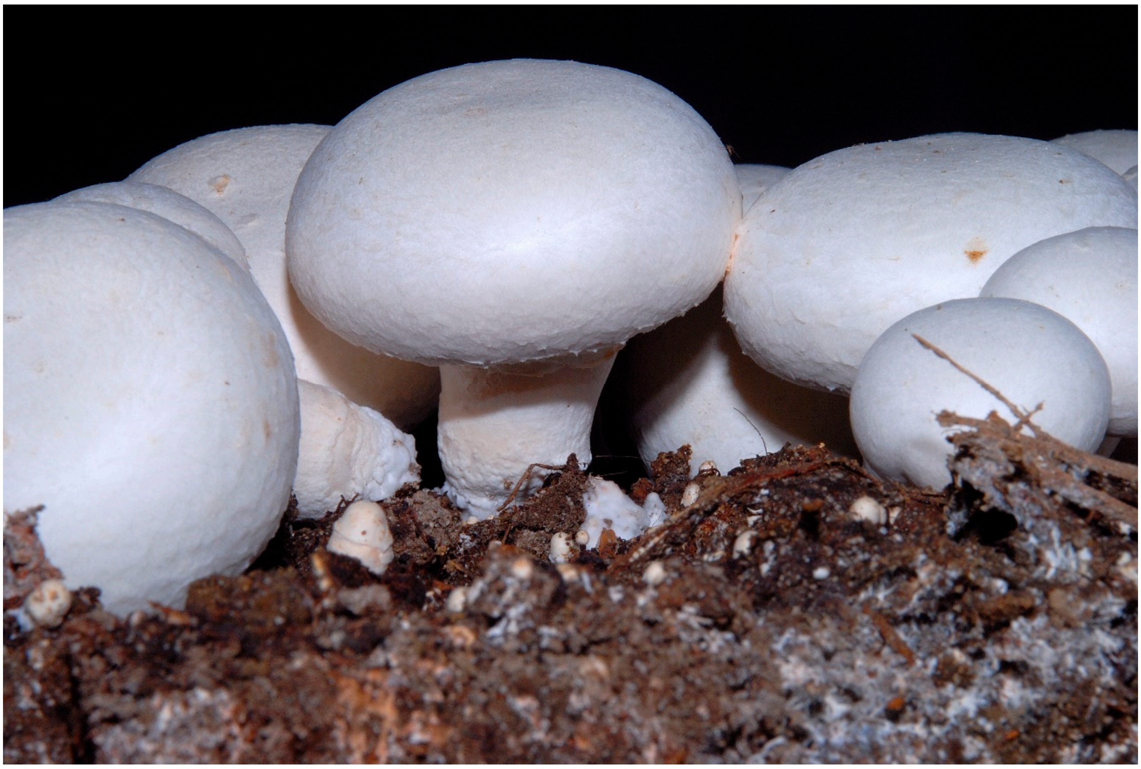

Figure S2. *Agaricus bisporus* — copper-enzyme-rich fungal system. Photograph courtesy of Antonio Lavagno.

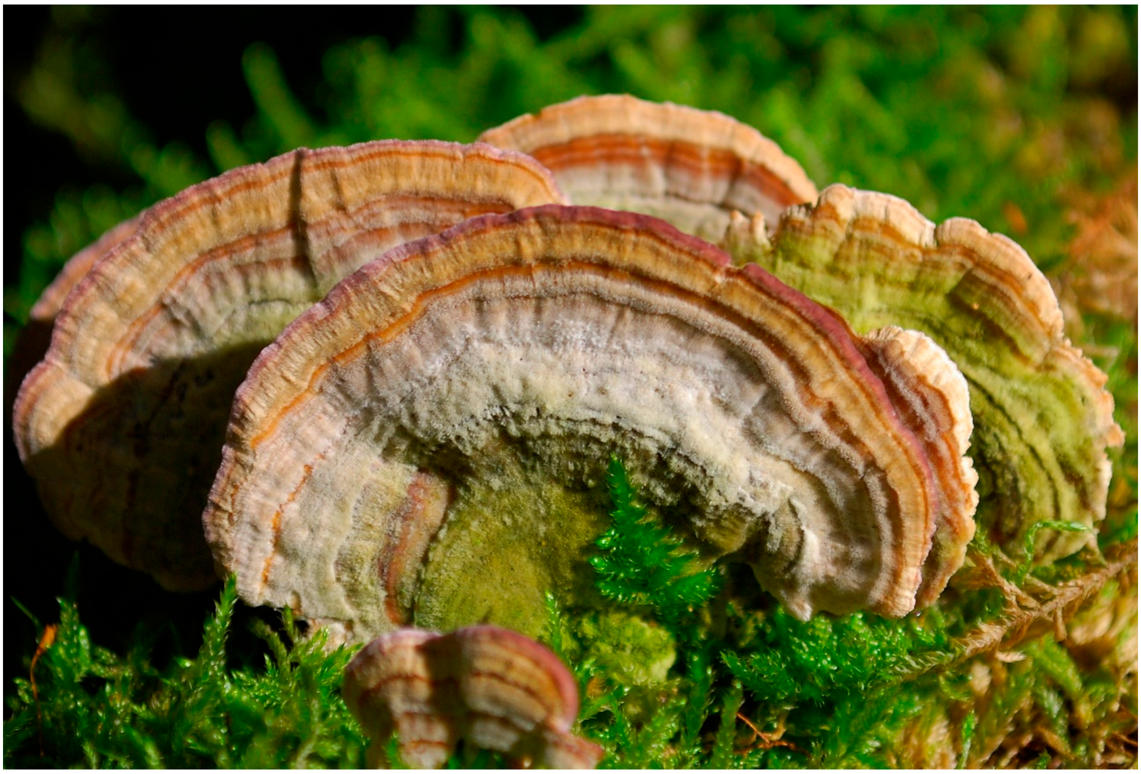

Figure S3. *Trametes versicolor* — redox-active and lipid-binding fungal system. Photograph courtesy of Antonio Lavagno.

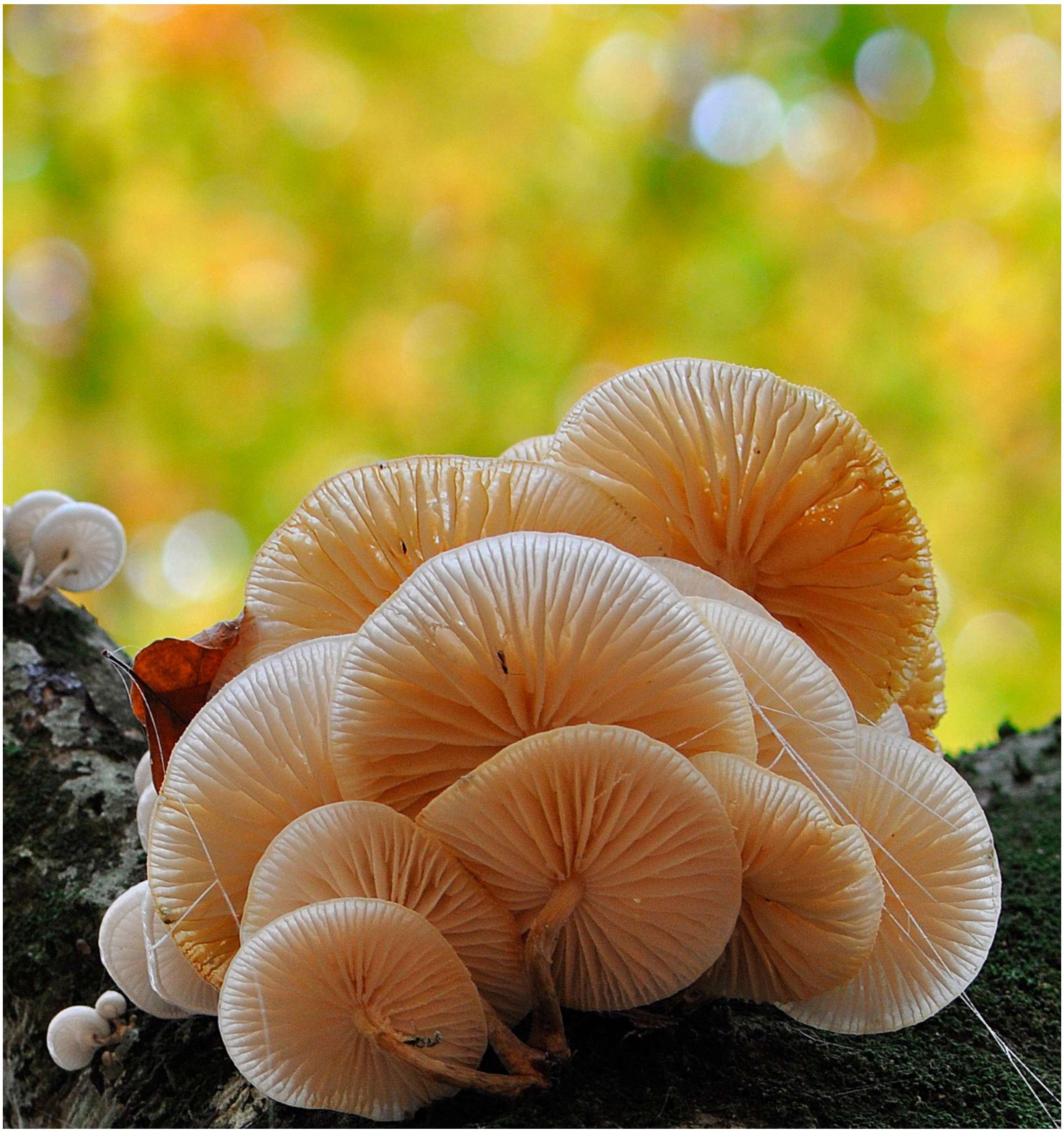

Figure S4. *Mucidula mucida* — lipid-binding fungal system. Photograph courtesy of Antonio Lavagno.

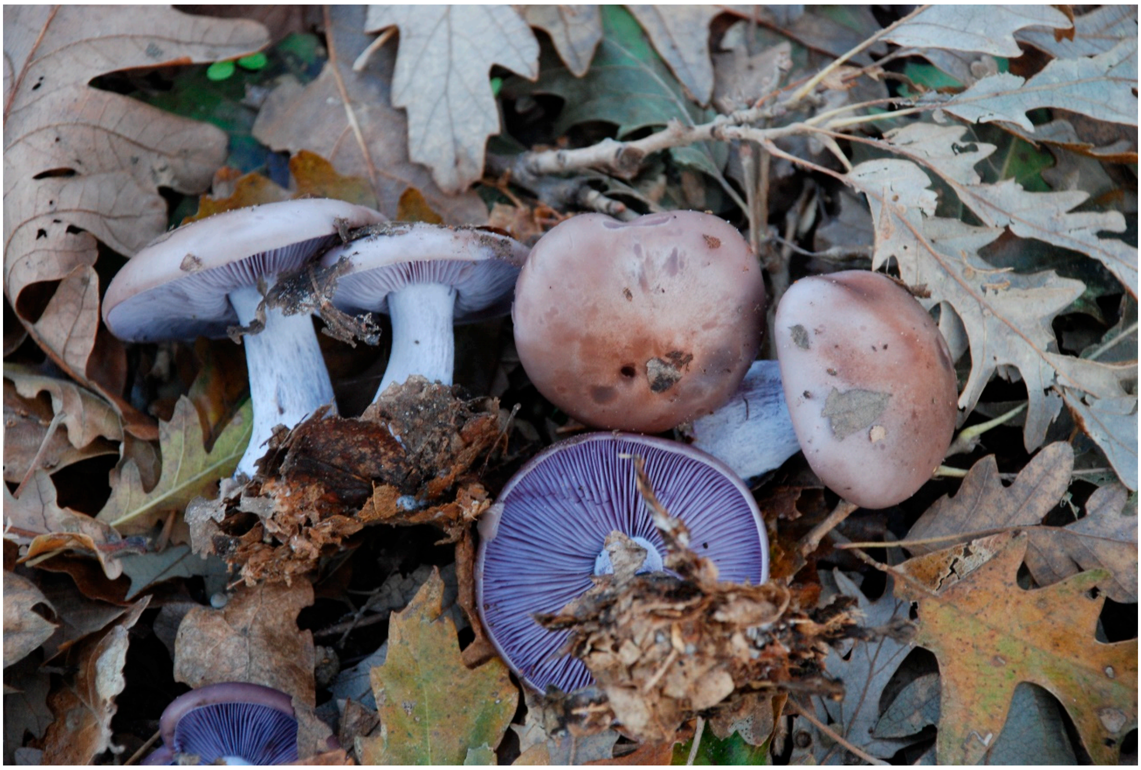

Figure S5. *Lepista nuda* — lipid-binding fungal system. Photograph courtesy of Antonio Lavagno.

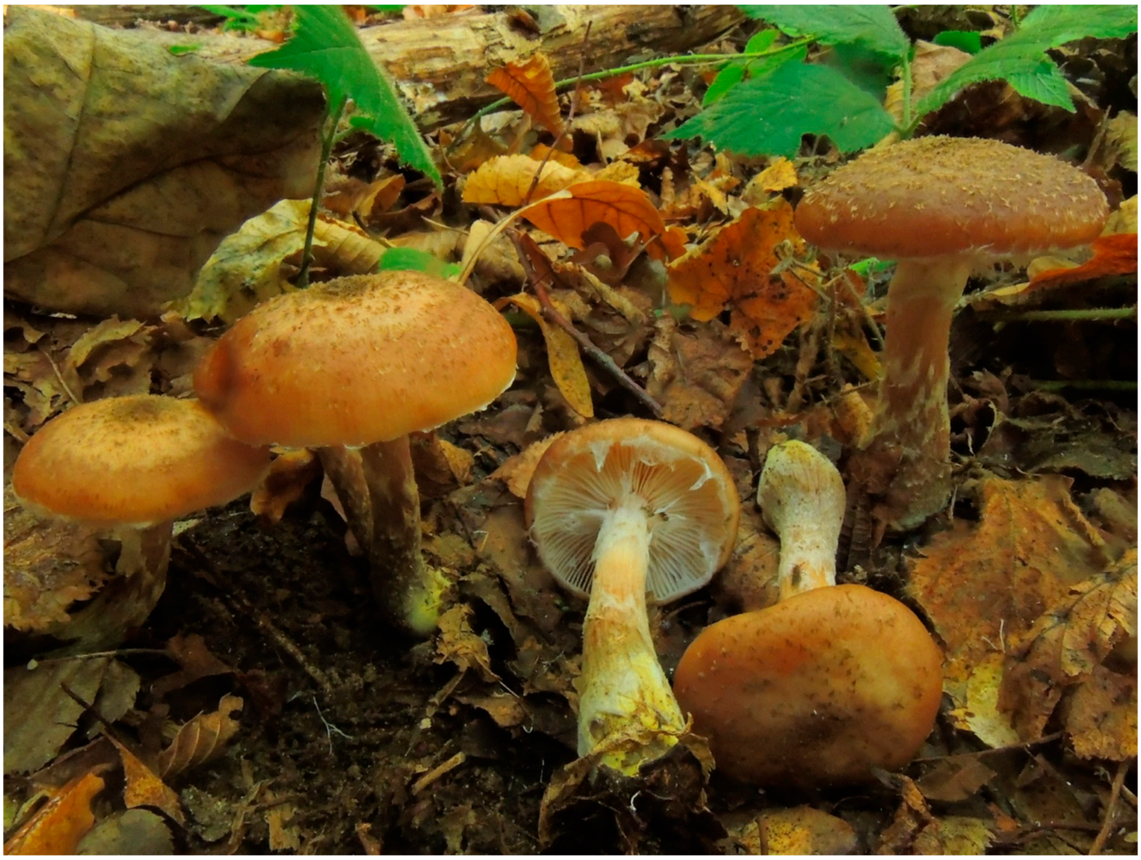

Figure S6. *Armillaria bulbosa* — early model for fungal bioelectrical studies. Photograph courtesy of Amedeo Schipani.

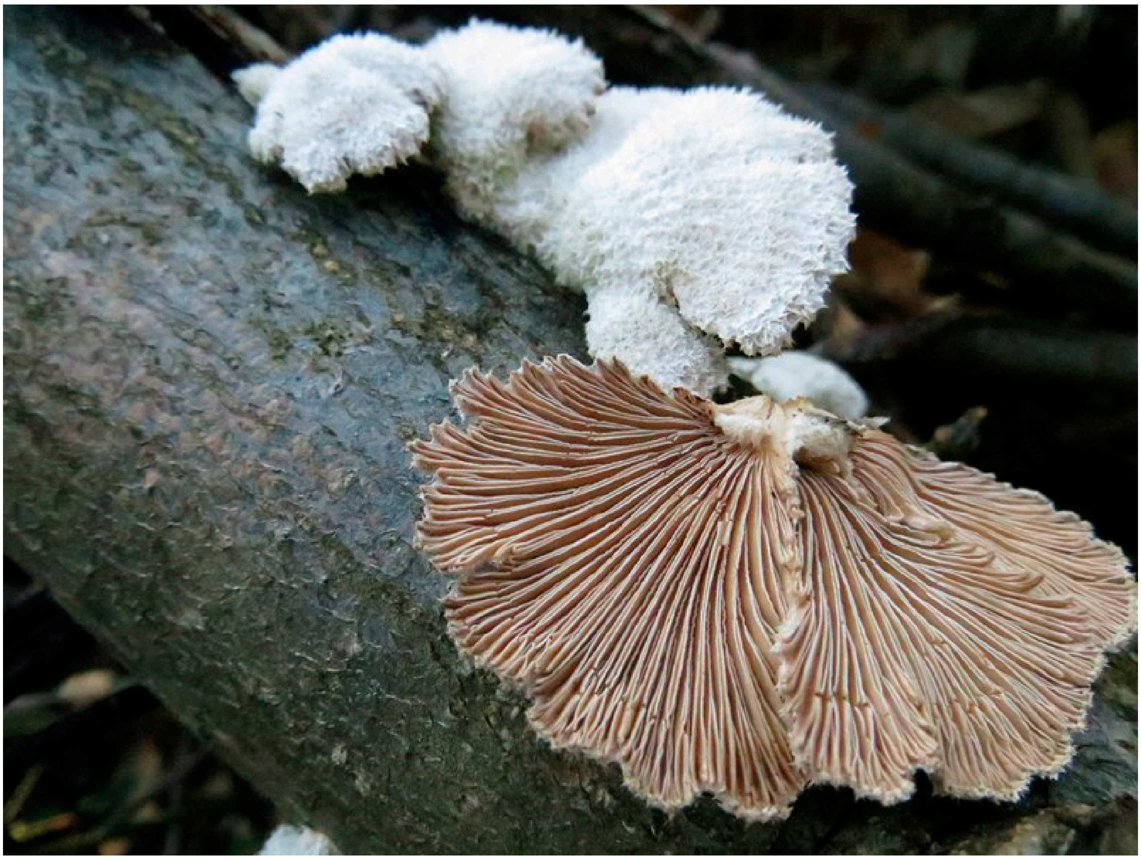

Figure S7. *Schizophyllum commune* — biomimetic and information-processing fungal system. Photograph courtesy of Enzo Ferri.

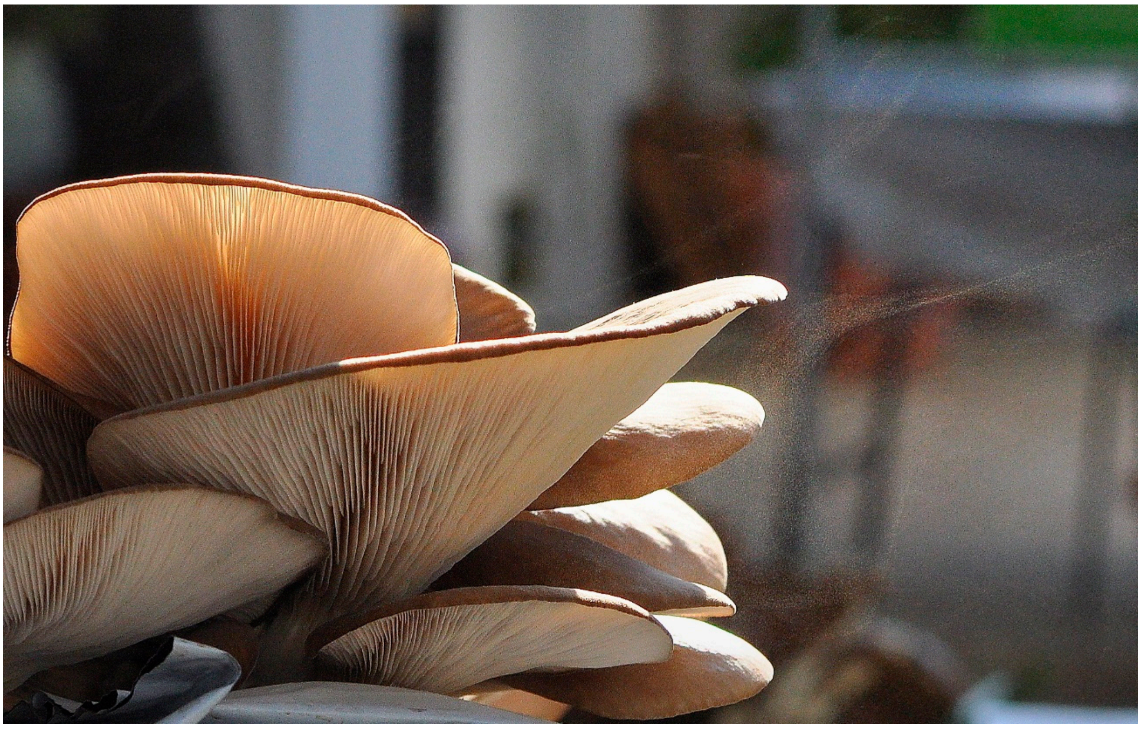

Figure S8. *Pleurotus ostreatus* — multifunctional living fungal system. Photograph courtesy of Antonio Lavagno.

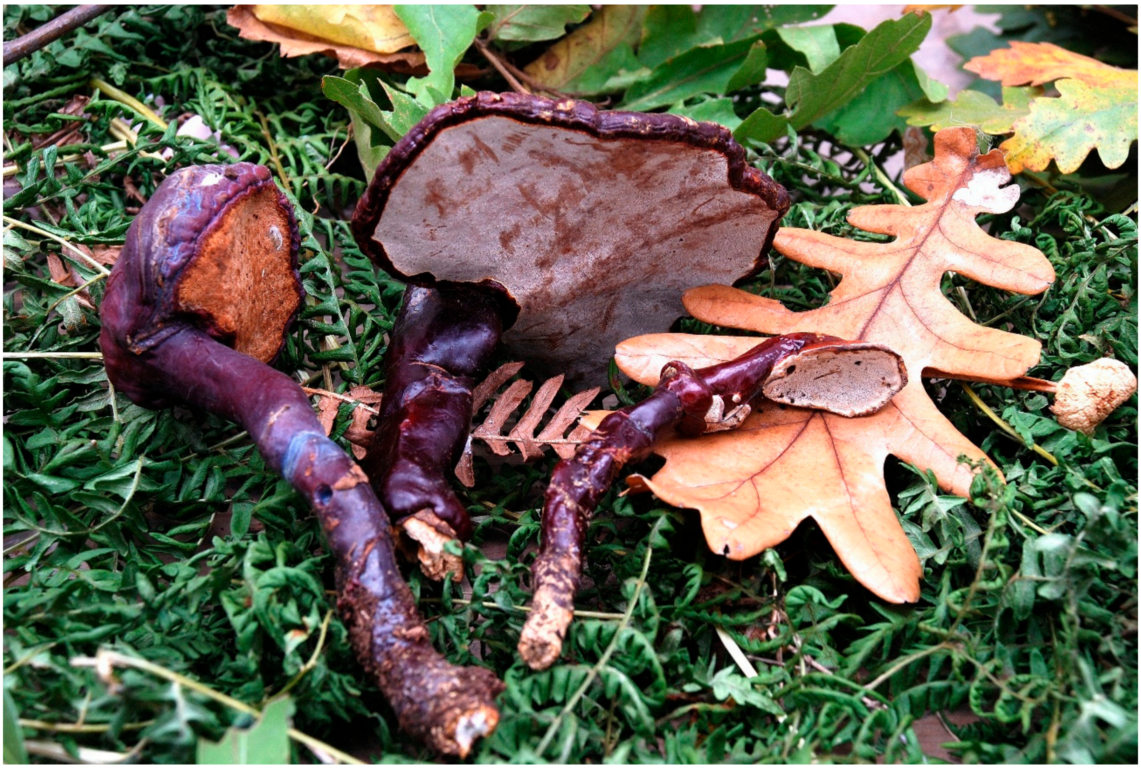

Figure S9. *Ganoderma lucidum* — multifunctional biohybrid fungal system. Photograph courtesy of Antonio Lavagno.
